# New Insights into the Organization, Recombination, Expression and Functional Mechanism of Low Molecular Weight Glutenin Subunit Genes in Bread Wheat

**DOI:** 10.1371/journal.pone.0013548

**Published:** 2010-10-21

**Authors:** Lingli Dong, Xiaofei Zhang, Dongcheng Liu, Huajie Fan, Jiazhu Sun, Zhongjuan Zhang, Huanju Qin, Bin Li, Shanting Hao, Zhensheng Li, Daowen Wang, Aimin Zhang, Hong-Qing Ling

**Affiliations:** State Key Laboratory of Plant Cell and Chromosome Engineering, National Center for Plant Gene Research, Institute of Genetics and Developmental Biology, Chinese Academy of Sciences, Beijing, China; Cairo University, Egypt

## Abstract

The bread-making quality of wheat is strongly influenced by multiple low molecular weight glutenin subunit (LMW-GS) proteins expressed in the seeds. However, the organization, recombination and expression of LMW-GS genes and their functional mechanism in bread-making are not well understood. Here we report a systematic molecular analysis of LMW-GS genes located at the orthologous *Glu-3* loci (*Glu-A3*, *B3* and *D3*) of bread wheat using complementary approaches (genome wide characterization of gene members, expression profiling, proteomic analysis). Fourteen unique LMW-GS genes were identified for Xiaoyan 54 (with superior bread-making quality). Molecular mapping and recombination analyses revealed that the three *Glu-3* loci of Xiaoyan 54 harbored dissimilar numbers of LMW-GS genes and covered different genetic distances. The number of expressed LMW-GS in the seeds was higher in Xiaoyan 54 than in Jing 411 (with relatively poor bread-making quality). This correlated with the finding of higher numbers of active LMW-GS genes at the *A3* and *D3* loci in Xiaoyan 54. Association analysis using recombinant inbred lines suggested that positive interactions, conferred by genetic combinations of the *Glu-3* locus alleles with more numerous active LMW-GS genes, were generally important for the recombinant progenies to attain high Zeleny sedimentation value (ZSV), an important indicator of bread-making quality. A higher number of active LMW-GS genes tended to lead to a more elevated ZSV, although this tendency was influenced by genetic background. This work provides substantial new insights into the genomic organization and expression of LMW-GS genes, and molecular genetic evidence suggesting that these genes contribute quantitatively to bread-making quality in hexaploid wheat. Our analysis also indicates that selection for high numbers of active LMW-GS genes can be used for improvement of bread-making quality in wheat breeding.

## Introduction

Bread wheat (*Triticum aestivum* L., AABBDD, 2n = 6x  = 42) provides basic staple food for a large proportion of the world population. A wide range of daily food products, such as bread, cakes, noodles and pastas, is prepared using dough that is generated by mixing wheat flour with water. The viscoelastic properties of dough, which are mainly affected by the strength and quantity of gluten, determine its suitability for being processed into different types of food products [1]. Through many decades of genetic and breeding studies, researchers have found that the prolamin seed storage proteins play important roles in determining gluten strength and quantity (and hence the viscoelastic properties of dough and end use qualities) of commercial wheat varieties [2]. The prolamins have been classified into two groups (gliadins and glutenins) according to their solubility in aqueous/alcohol solutions [3]. The gliadins are monomeric proteins, and contribute mainly to dough extensibility [4]–[6]. By contrast, the glutenins are polymeric proteins, and play a major role in dough elasticity [1]. The glutenins are further divided into high molecular weight subunits (HMW-GS) and low molecular weight subunits (LMW-GS) based on differences in molecular mass [7], [8]. In bread wheat, the genes encoding HMW-GS proteins are located at the orthologous loci *Glu-A1*, *Glu-B1* and *Glu-D1*, each containing two paralogous genes encoding one x- and one y-type subunit [1]. Through genetic, breeding and transgenic studies, it has been found that the expression of certain subunits (i.e., 1Dx5, 1Ax1, 1Bx7^OE^) is correlated with superior bread-making quality [9]–[12]. Specific selections of these subunits in conventional and molecular breeding programs have contributed to the genetic improvement of bread-making quality in worldwide wheat production [7], [13]–[15].

Compared to HMW-GS proteins, substantially more LMW-GSs are expressed in the grains of bread wheat. The primary structure of a typical LMW-GS is generally composed of a signal peptide (removed from mature protein), a relatively short N-terminal domain, a central repetitive domain, and a C-terminal domain. Based on the first amino acid residue in the mature protein, LMW-GSs have been grouped into three types, LMW-m, LMW-s and LMW-i, which have methionine, serine and isoleucine at the beginning of their mature subunit proteins, respectively [16]–[18]. The three types of subunits also differ significantly in other important aspects. The molecular mass of LMW-m subunits (30∼40 kD) is generally smaller than that of LMW-s proteins (35∼45 kD). The primary structure of LMW-i subunits differ from that of LMW-s and LMW-m proteins in lacking the short N-terminal domain [19]. Central to the structure and function of typical LMW-GSs is the presence of a number of conserved cysteine residues in their proteins, but the relative positions of these cysteine residues may vary among different types of subunits [20], [21].

The multiplicity of LMW-GSs and the difficulty in distinguishing LMW-GSs from gliadins in SDS-PAGE have complicated efforts to understand the physical and genetic organization of the chromosomal loci containing LMW-GS genes. It is now generally accepted that the great majority of typical LMW-GSs is encoded by the genes situated at the orthologous *Glu-3* loci (*Glu-A3*, *Glu-B3*, *Glu-D3*) on the short arms of group 1 chromosomes (1AS, 1BS and 1DS) [21], [22]. *Glu-A3*, *Glu-B3* and *Glu-D3* are closely linked with the *Gli-A1*, *Gli-B1* and *Gli-D1* loci, respectively, which encode gliadins [22], [23]–[25]. Through sequencing bacterial artificial chromosome (BAC) clones derived from the short arms of 1A and 1B of the tetraploid wheat cultivar (cv.) Langdon, Gao *et al.* [26] provided the first insight into the physical relationships of *Glu-3* and *Gli-1* genes. In a 265 kb BAC contig derived from the short arm of 1AS, there were five gliadin and gliadin-like genes and two typical LMW-GS genes, and in a 140 kb genomic DNA fragment derived from 1BS, there were eight gliadin genes and one typical LMW-GS gene [26]. The gliadin genes appeared to be closely spaced, whereas the two LMW-GS genes in the sequenced 1AS region were separated from each other by about 100 kb [26]. While studying the *Glu-3* locus of *Triticum monococcum*, which is diploid and possesses a genome homoeologous to the A genome of common wheat, Wicker *et al.* [27] also found a large intergenic distance between two neighboring LMW-GS genes (approximately 150 kb). Moreover, non-prolamin genes (such as *Pm3* analog, *LrK10*) and non-coding genetic markers (i.e., *SFR159*, *WHS179*) occurred in close proximities to LMW-GS genes [26]–[29]. Together, these observations indicate that the *Glu-A3* and *B3* loci may be highly complex and their size may be quite large. To date, no sequencing data have been reported for any BAC clones containing genomic DNA from the *Glu-D3* locus of wheat (or related *Triticeae* species). In addition, the numbers of BAC clones sequenced in the reported studies are generally small (less than three [26], [27]), making it difficult to examine the detailed organizational characteristics of LMW-GS genes at the *Glu-3* loci. The genetic organization of LMW-GS genes at the othologous *Glu-3* loci and their recombination characteristics are also not well understood. Although many investigators have placed *Glu-3* markers in linkage maps of group 1 chromosomes constructed using specific genetic populations [30]-[38], generally no information is available on the number and molecular structure of the LMW-GS genes represented by such *Glu-3* markers. One study mapped three LMW-GS genes on the short arm of chromosome 1D (1DS) of *Aegilops tauschii*, the D genome donor of hexaploid wheat [39], [40], and suggested the occurrence of high recombination in the chromosomal region harboring *Glu-D3* loci [28]. However, no detailed information is currently available on the recombination characteristics of LMW-GS genes at the *Glu-A3* or *B3* loci.

Because of the importance of LMW-GS genes in bread-making quality (see below), considerable effort has been devoted to investigate the numbers and characteristics of the LMW-GS genes in different bread wheat varieties. For example, Ikeda *et al.* [41] found 12 LMW-GS genes (three at *Glu-A3*, two at *Glu-B3*, and seven at *Glu-D3*) in cv. Norin 61 by analyzing PCR products amplified with oligonucleotide primers specific for this group of genes. By analyzing selected BAC clones, Huang and Cloutier [42] identified 12 active and seven inactive LMW-GS genes in cv. Glenlea. Among the 12 active members, nine were assigned to *Glu-D3* (GenBank accessions EU189090-EU189098), two to *Glu-B3* (EU189088-EU189089), and one to *Glu-A3* (EU189087). Consistent with the previous findings [26], [27], a large intergenic distance (about 81 kb) was found between any two neighboring LMW-GS genes in Glenlea. Both studies revealed that *Glu-D3* contained more LMW-GS genes than *Glu-A3* or *Glu-B3*. In line with these findings, seven distinct genes encoding LMW-GS proteins were found in an *Aegilops tauschii* accession [43]. Concomitant to the attempts to find the total number of LMW-GS genes in particular genotypes, another line of research was to identify distinct LMW-GS genes and their haplotypes in large groups of wheat varieties. Using this approach, a total of six active *Glu-D3* LMW-GS genes with 12 haplotypes were isolated [44], [45]. The six *Glu-D3* LMW-GS genes encode five m- and one s-type subunits. Four active *Glu-B3* LMW-GS genes with 17 haplotypes were reported [46]. The four *Glu-B3* LMW-GS genes specify one m- and three s-type subunits. One early study identified seven active *Glu-A3* LMW-GS alleles (all coding for i-type subunits) [47], but it is still unclear as to how many distinct LMW-GS genes were originally involved. Collectively, the above data indicate that each *Glu-3* locus may contain multiple LMW-GS genes with rich allelic variations, but do not provide a complete elucidation of the LMW-GS genes at individual *Glu-3* loci in particular wheat genotypes, and do not establish specific correspondence between LMW-GS genes and their protein products accumulated in the seeds.

The function of LMW-GS proteins in controlling end use qualities of wheat grains has been studied in both tetraploid and hexaploid varieties. Pioneering investigations in durum wheat identified a superior LMW-GS allele (*LMW-2*) conferring improved pasta-making quality [48], [49]. The functional superiority of *LMW-2* was mainly associated with the expression of more LMW-GS species from its locus than the contrasting allele [18], [21], [50], [51]. In bread wheat, multiple protein alleles were discovered for each of the three *Glu-3* loci based on their differences in electrophoretic mobility in SDS-PAGE [52], and these alleles differed in their effects on certain aspects of bread-making quality [53]–[55]. However, it is still not clear if the particular allele *per se*, or the locus in which the examined allele is located, is responsible for observed functional differences. More recent breeding, quantitative genetic, and modeling studies generally found positive contributions of *Glu-3* loci to parameters related to dough strength, extensibility and bread-making quality [13], [30], [32]–[36], [38], [56], [57]. But the molecular genetic mechanisms underlying the functional differences of orthologous or allelic *Glu-3* loci are still not well investigated.

From the information presented above, the main objectives of this work were to gain new information on the organization and recombination characteristics of LMW-GS genes at *Glu-3* loci, and on the molecular genetic mechanism underlying their function in bread-making quality. To achieve these goals, complementary approaches (gene identification through sequencing selected BAC clones and PCR amplification, transcript profiling, and proteomic analysis) were used to study the LMW-GS genes and their expression in the seeds of bread wheat cultivars Xiaoyan 54 and Jing 411, with superior and poor bread-making qualities, respectively [58]–[60]. The relative genetic positions and recombination characteristics of LMW-GS genes at orthologous *Glu-3* loci were studied in Xiaoyan 54. The differential contributions of allelic *Glu-3* loci to Zeleny sedimentation value (an indicator of gluten strength and bread volume) were investigated by association analysis using recombinant inbred lines (RILs) developed from a cross between Xiaoyan 54 and Jing 411.

## Results

### Identification and analysis of BAC clones containing LMW-GS genes

A BAC library of Xiaoyan 54 was constructed and subjected to several rounds of PCR screening using primers (Table S1) specific for LMW-GS genes, resulting in the identification of 27 positive BAC clones (Table 1). Pulse field gel electrophoresis (PFGE) analysis revealed that the insert size of these clones ranged from 25 to 140 kb. Southern hybridization on BAC DNA digested with *Not*I showed that the majority of the analyzed clones yielded one hybridizing band, except for clone D1862-8-2 that showed two positive bands (Figure S1). Similar results were obtained when the hybridization was performed with DNA samples digested with two restriction enzymes (*Not*I, *Sal*I), although in this case two hybridizing bands were observed for BAC clone B57-6-5 (Figure S1). Collectively, the data indicated that there may be one to two LMW-GS genes in each of the selected BAC clones. This finding is consistent with previous observations on LMW-GS genes in the BAC clones derived from *Ae. tauschii* [43], the tetraploid wheat cv. Langdon [26], and the bread wheat cv. Glenlea [42].

**Table 1:** The 14 LMW-GS genes of Xiaoyan 54 isolated by BAC sequencing and PCR amplification, and the main characteristics of subunits deduced from active LMW-GS gene members.

Gene	BAC clone a	Chromosomallocation	Subunit(type/aa)	Repeat unit	Deduced mass(Da)
*A3-1*	**A1056-11-5** (30)	1AS	m/304	13	34277.8
*A3-2*	**A708-12-2** (113), **A1380-8-2** (116)		i/358	20	41248.3
*A3-3*	**A1154-1-1** (43), A1154-1-2 (35)		– c	–	–
*A3-4*	NI b		i/390	23	45054.7
*B3-1*	**B229-8-**7 (125), B498-5-8 (65),B1354-3-6 (80), B1354-4-8 (80), B1777-3-8 (60)	1BS	m/350	19	39828.4
*B3-2*	**B57-6-5** (80)		s/392	26	44529.7
*B3-3*	**B57-6-5** (80)		– d	–	–
*D3-1*	**D78-6-8** (64), **D357-11-6** (83),D570-9-3 (75)	1DS	m/365	21	41689.2
*D3-2*	**D1862-8-4** (62)		m/304	15	34609.6
*D3-3*	**D1862-8-4** (62)		s/354	21	40024.3
*D3-4*	D43-2-5 (25), **D510-5-7** (80),D528-3-6 (50), D603-1-8 (85),D722-12-7 (45), D769-8-7 (65),D774-5-8 (100), D1083-4-6 (30)		m/298	13	33853.8
*D3-5*	**D479-7-6** (53), D948-5-8 (140)		– e	–	–
*D3-6*	**D1126-1-3** (106)		m/350	18	39805.2
*D3-7*	**D1220-5-2** (70)		m/299	13	33655.2

a The BAC clones in bold were selected for DNA sequencing analysis. The insert sizes (kb) are in brackets.

b *A3-4* was isolated by PCR amplification, and assigned to 1AS through genetic mapping.

c An i-type subunit pseudogene caused by the presence of a premature stop codon in the coding region.

d A s-type subunit pseudogene due to transposon insertion in the coding region.

e A m-type subunit pseudogene due to the presence of frame shift mutation in the coding region.

The LMW-GS gene sequences in the positive BACs were amplified by high fidelity PCR with the same primers used in BAC library screening. The resultant PCR products were cloned and sequenced. Based on similarity analysis of the nucleotide sequences, 12 different LMW-GS gene sequences, representing 12 unique LMW-GS genes, were identified from 27 BACs. The suggested names of the 12 genes are given in Table 1. The 27 BAC clones were then assigned to individual group 1 chromosomes (1A, 1B, and 1D) by PCR mapping using genomic DNA samples extracted from the nulli-tetrasomic (NT) lines of group 1 chromosomes of Chinese Spring (CS) and the markers derived from LMW-GS genes or BAC end sequence information (Table S2). The numbers of BAC clones assigned to 1A, 1B and 1D chromosomes were five, six, and 16, respectively (Table 1).

Thirteen BAC clones, likely representing the maximum coverage of *Glu-A3* (four clones), *Glu-B3* (two clones), and *Glu-D3* (seven clones) loci (Table 1), were chosen for DNA sequencing analysis. Twelve BAC clones were completely sequenced. The insert in B57-6-5 was only partially sequenced due to the presence of too many repeat sequences. Examination of BAC sequence data verified the nucleotide sequences of 12 LMW-GS genes (*A3-1*, *A3-2*, *A3-3*, *B3-1*, *B3-2*, *D3-1*, *D3-2*, *D3-3*, *D3-4*, *D3-5, D3-6* and *D3-7,* Table 1) determined initially using PCR amplified fragments (Accession numbers FJ755302, FJ755303, FJ755304, FJ755306, FJ755309, FJ755310, FJ755311, FJ755312, FJ75513, FJ755314, FJ755315, FJ755316 ). In addition, another LMW-GS gene sequence (*B3-3*, accession number FJ755307) with its coding region interrupted by transposon insertion was found in B57-6-5.

### Analysis of LMW-GS genes and their associations with known markers and genes

**Figure 1:**
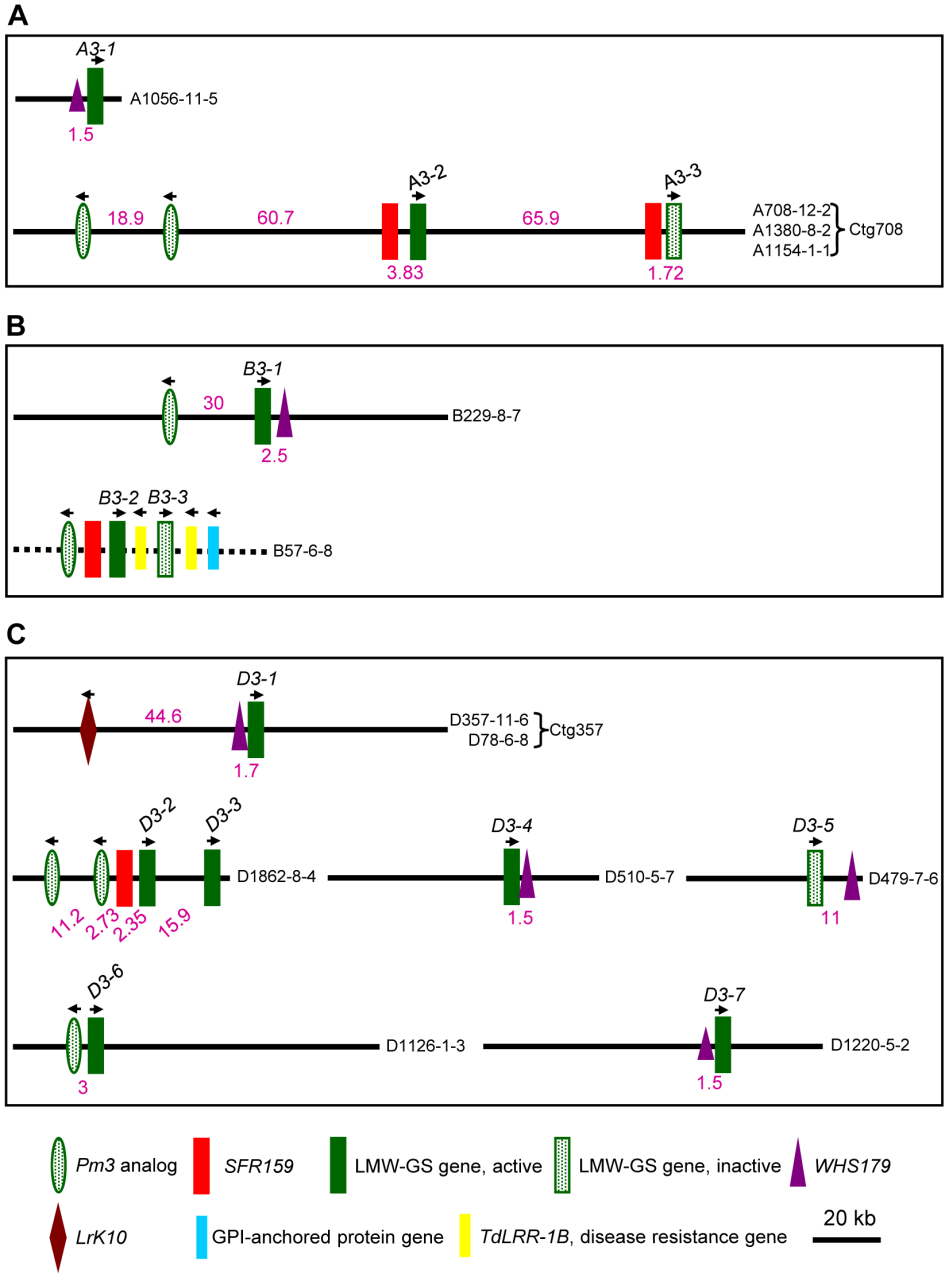
The organizations of LMW-GS genes of Xiaoyan 54 in 13 representative BAC clones. Diagrams illustrating the organizations of the LMW-GS genes (including both active and inactive members) of Xiaoyan 54 in 13 representative BAC clones, in relation to the markers (*SFR159* and *WHS179*) and genes (*LrK10*, *Pm3* analog, *TdLRR-1B* disease resistance gene, and the gene encoding GPI-anchored protein) found in the vicinities of LMW-GS genes in previous studies. Sequence gaps exist in the BAC clone B57-6-5 (drawn as a dashed line). The remaining 12 BAC clones were fully sequenced. If available, the physical distances (kb) between two neighboring genes or between a marker and its adjacent gene are given (in purple). The arrows indicate the predicted transcriptional directions of the genes. (A) The organization patterns of three *Glu-A3* LMW-GS gene members. A *WHS179* marker was present in BAC clone A1056-11-5, and its physical distance to *A3-1* was 1.5 kb. *A3-2* and *A3-3* occur in the BAC contig Ctg708 formed by three BAC clones (A708-12-2, A1380-8-2, A1154-1-1). (B) The organization patterns of three *Glu-B3* LMW-GS gene members (*B3-1*, *B3-2*, *B3-3*). The physical distances between neighboring genes or the adjacent gene and marker were not determined in B57-6-5 because of the presence of sequence gaps. (C) Organization patterns of seven *Glu-D3* LMW-GS gene members (*D3-1*, *D3-2*, *D3-3*, *D3-4*, *D3-5*, *D3-6*, *D3-7*). *D3-1* was found in the contig Ctg357 composed of two BAC clones (D357-11-6, D78-6-8).

Sequence analysis revealed that A708-12-2, A1380-8-2 and A1154-1-1 from chromosome 1A were overlapping BACs, which formed a contig (Ctg708, Figure 1A) of ∼210 kb and contained *A3-2* and *A3-3* (Figure 1A). The distance between *A3-2* and *A3-3* was 67.6 kb. By contrast, BAC clone A1056-11-5, containing *A3-1* (Figure 1A), was a singleton. Within Ctg708, *A3-2* and *A3-3* were each closely associated with marker *SFR159* (less than 4 kb between marker and gene). There were two *Pm3* disease resistance gene analogs upstream of *A3-2* and *A3-3* (Figure 1A). *WHS179* was present in A1056-11-5, but not found in Ctg708 (Figure 1A). Conceptual translation showed that *A3-1* and *A3-2* encoded m- and i-type subunits, respectively (Table 1). *A3-3* represented an i-type subunit pseudogene because its coding region was disrupted by a premature stop codon (Table 1). The close relationship of *SFR159* and the LMW-GS gene in Ctg708 resembled that at the *Glu-A^m^3* locus of *T. monococcum* [27]. However, there were three LMW-GS genes at *Glu-A^m^3* (*TmGlu-A3-1, 2*, *3*), compared to the two LMW-GS genes in Ctg708. To test if there was another LMW-GS gene located adjacent to Ctg708, a genomic PCR experiment was conducted using primers specific for i-type LMW-GS gene sequences (Table S1). After cloning and sequencing the amplified fragments, a third i-type LMW-GS gene sequence, sharing more than 88% nucleotide identity to *A3-2* and *A3-3*, was identified. This newly identified LMW-GS gene (*A3-4*, accession number FJ755305) contained an intact ORF, giving rise to an i-type subunit upon conceptual translation (Table 1).

For the two 1B BAC clones (Table 1), B229-8-7 (containing *B3-1*) was 124 kb in length, and contained one *WHS179* marker but not *SFR159* (Figure 1B). Although not fully assembled, B57-6-5 (containing *B3-2* and *B3-3*) carried several non prolamin genes, including one *Pm3* analog, two similar *TdLRR-1B* sequences [26], one ORF for a putative GPI-anchored protein, and one *SFR159* marker (Figure 1B). The co-presence of LMW-GS gene, *Pm3* analog, *TdLRR-1B*, *SFR159* and GPI-anchored protein ORF in B57-6-5 resembled the structural organization in BAC clone BAC419P13 derived from the *Glu-B3* locus of durum wheat [26], except that B57-6-5 possessed two LMW-GS genes and two *TdLRR-1B* sequences. Based on this similarity, the gene and marker order in B57-6-5 was tentatively arranged as shown in Figure 1B. *B3-1* and *B3-2* encoded m- and s-type subunits, respectively, whereas *B3-3* represented a s-type subunit pseudogene due to transposon insertion in its coding region (Table 1).

For the seven 1D clones subjected to DNA sequencing analysis (Table 1), D78-6-8 and D357-11-6 formed a contig (Ctg357), which was about 128.7 kb and contained *D3-1* (Figure 1C). *D3-2* and *D3-3* were found in D1862-8-4 with a distance of 15.9 kb, whereas *D3-4*, *D3-5*, *D3-6* and *D3-7* were in D510-5-7, D479-7-6, D1126-1-3 and D1220-5-2, respectively (Figure 1C). *WHS179* was found in Ctg357, D510-5-7, D479-7-6 and D1220-5-2, whereas *SFR159* was present only in D1862-8-4 (Figure 1C). *Pm3* analogs were detected in D1862-8-4 and D1126-1-3 (Figure 1C). Among the six *Glu-D3* LMW-GS genes with intact ORF, five (*D3-1*, *D3-2*, *D3-4*, *D3-6*, *D3-7*) encoded m-type subunits, one (*D3-3*) specified a s-type subunit, and the remaining one (*D3-5*) was a m-type subunit pseudogene because of the presence of a premature stop codon in the coding region (Table 1).

### Genetic mapping and recombination of LMW-GS genes at *Glu-3* loci

A mapping strategy, utilizing the 182 RILs from the Xiaoyan 54× Jing 411 cross, was undertaken to map the 14 LMW-GS genes at *Glu-3* loci in Xiaoyan 54. This was facilitated by the development of specific and polymorphic markers for *A3-1*, *A3-2*/*A3-3*/*A3-4*, *B3-1*, *B3-2*/*B3-3*, *D3-1*, *D3-2*/*D3-3*, *D3-4*, *D3-5*, *D3-6* or *D3-7* (Table S2). A preliminary investigation showed that *A3-2, A3-3* and *A3-4* were inherited as a single genetic cluster in the 182 RILs (data not shown). A polymorphic marker developed from the *A3-2* sequence was therefore used for mapping the location of this cluster. A similar situation occurred for *B3-2*/*B3-3* and *D3-2*/*D3-3*. Consequently, the markers derived from the *B3-2* and *D3-2* sequences were used for investigating the locations of the *B3-2*/*B3-3* and *D3-2*/*D3-3* clusters, respectively. To facilitate the comparisons of our mapping data with those published previously, the positional relationships between the LMW-GS genes isolated in this work and the microsatellite markers previously mapped to the 1AS, 1BS or 1DS chromosome arms were also investigated. A total of 23 markers (seven on 1AS, 11 on 1BS, and five on 1DS), exhibiting polymorphisms between Xiaoyan 54 and Jing 411, was mapped along with the different LMW-GS genes (see below).

**Figure 2:**
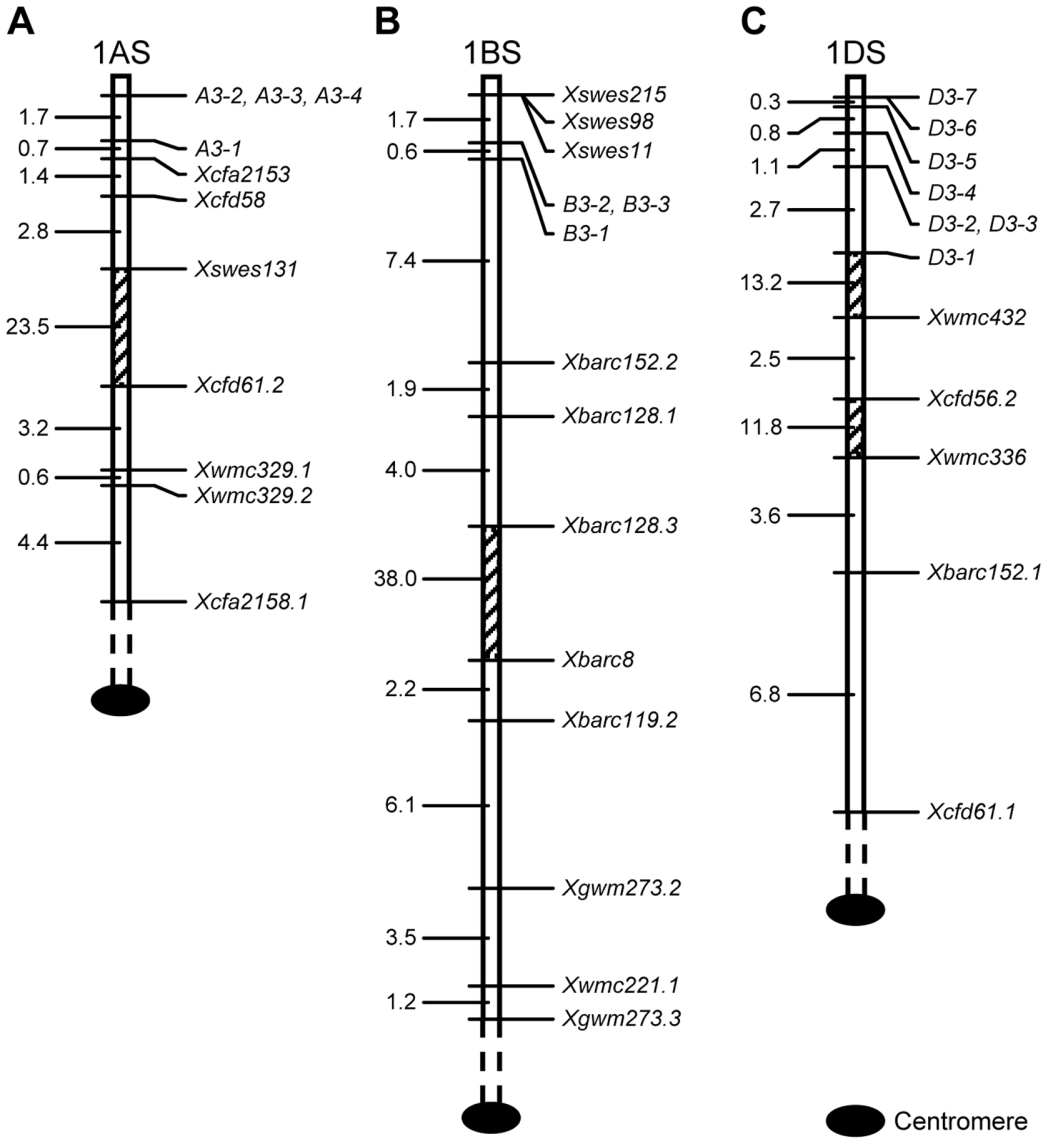
Relative genetic positions of the LMW-GS genes of Xiaoyan 54 in homoeologous group 1 chromosomes. The names of the LMW-GS genes and previously published markers are on the right side, whilst the genetic distance values (cM) between neighboring genes (gene and marker, or markers) are on the left. The hatched areas in the three maps, representing chromosomal regions with large map distances (>10 cM), are not drawn in scale. (A) The map positions of four *Glu-A3* LMW-GS genes in relation to seven published markers on 1AS. (B) The map positions of three *Glu-B3* LMW-GS genes in relation to 11 published markers on 1BS. (C) The map positions of seven *Glu-D3* LMW-GS genes in relation to five published markers on 1DS.

Among the four LMW-GS genes at *Glu-A3*, *A3-1* was proximal, whereas *A3-2*/*A3-3*/*A3-4* was distal, to the centromere (Figure 2A). Recombination occurred between *A3-1* and the *A3-2*/*A3-3*/*A3-4* cluster, with an estimated genetic distance of 1.7 cM (Figure 2A). Seven previously reported microsatellite markers were proximal to *A3-1* (Figure 2A). The cumulative genetic distance from *A3-2*/*A3-3*/*A3-4* to *Xcfa2158.1* was 38.3 cM.

Among the three LMW-GS genes at *Glu-B3*, *B3-1* was more proximal to the centromere than the *B3-2*/*B3-3* cluster (Figure 2B). The estimated genetic distance between *B3-1* and *B3-2*/*B3-3* was 0.6 cM. Three previously described *Xswes* markers were more distal to the centromere than *B3-2*/*B3-3* (Figure 2B). Another set of eight published microsatellite markers were assigned to locations proximal to *B3-1* (Figure 2B). The total genetic distance covered from *B3-2*/*B3-3* to *Xgwm273.3* was 64.9 cM (Figure 2B).

For the seven LMW-GS genes at *Glu-D3*, *D3-1* was proximal to other six members. No recombination was detected between *D3-6* and *D3-7* (Figure 2C). By contrast, recombinations occurred between *D3-1* and *D3-2*/*D3-3*, *D3-2*/*D3-3* and *D3-4*, *D3-4* and *D3-5*, or *D3-5* and *D3-6*, with the calculated genetic distances ranging from 0.3 to 2.7 cM (Figure 2C). In total, the seven LMW-GS genes at *Glu-D3* covered 4.9 cM. Five previously reported microsatellite markers were mapped to locations proximal to *D3-1*, and covering a total genetic distance of 42.8 cM extending from *D3-6*/*D3-7* to *Xcfd61.1* (Figure 2C).

A comparison of the data displayed in Figures 1 and 2 reveal that the *WHS179* marker is present in all three genes (*A3-1*, *B3-1*, *D3-1*) located closest to the centromere. This is consistent with the previous finding showing that, on the short arm of group 1 chromosomes, *WHS179* is proximal to *SFR159* [29]. The *D3-4* and *D3-5* genes, which were accompanied by *WHS179* but not *LrK10* (Figure 1C), were mapped to a location more distal than *D3-1* which is associated with both *WHS179* and *LrK10* (Figure 2C). This agrees with the observation made by Spielmeyer *et al.* [28], indicating that there are at least two *WHS179* markers on the short arm of chromosome 1D in *Ae. tauschii*, with the one proximal to the centromere being associated with *LrK10* [28].

### Primary structure analysis and comparisons with published LMW-GS genes

Our results showed that the LMW-GS gene family in Xiaoyan 54 was composed of at least 14 distinct members. Except for *A3-3*, *B3-3* and *D3-5*, 11 members possessed intact ORF, coding for two i-type, seven m-type, and two s-type subunits, respectively (Table 1). The molecular mass of the deduced subunits varied from 33,655.2 to 45,054.7 Da (Table 1). Amino acid sequence comparisons showed that the primary structure of the deduced subunits generally resembled that described previously for typical i-, m- or s-type LMW-GS proteins (Figure S2). All 11 deduced subunits had intact repetitive and C-terminal domains (Figure S2), although the number of repeat units differed among the members (Table 1). Furthermore, eight cysteine residues were present in each of the 11 deduced proteins (Figure S2). *A3-1*, predicted to encode a m-type subunit with a putative N-terminal domain sequence MDTSCIPGLERPW, was located at *Glu-A3* (Table 1, Figures 2A and S2).

The 14 LMW-GS genes of Xiaoyan 54 were compared mainly to the seven *Glu-A3* alleles (*Glu-A3a* to *Glu-A3g*) reported by Zhang *et al.* [47], the four *Glu-B3* genes (*GluB3-1* to *GluB3-4*) defined by Wang *et al.* [46], or the six *Glu-D3* genes (*GluD3-1* to *GluD3-6*) identified by Zhao *et al.* [44], [45]. This was done because the three groups of genes (alleles) were all isolated from wheat genotypes with diverse genetic backgrounds, and were more likely to capture the molecular diversity of LMW-GS genes in the overall wheat gene pool. Among the four *Glu-A3* genes of Xiaoyan 54, nucleotide sequence comparisons showed that *A3-2* and *A3-3* both exhibited the highest identity (>99%) to *Glu-A3d*, whereas *A3-4* displayed the highest identity (94.3%) to *Glu-A3b* (Table S3). However, the identities of *A3-1* to the seven *Glu-A3* alleles were generally less than 60% (Table S3). Actually, *A3-1* was more than 97% identical to the two *Glu-A3* LMW-GS sequences represented by GenBank accessions AB062868 (from the *Glu-A3* locus of the bread wheat cv. Norin 61 [20]) and AJ293099 (from the *Glu-A3* locus of the durum wheat cv. Langdon), respectively.

Similar nucleotide sequence comparisons suggested that, among the three *Glu-B3* genes of Xiaoyan 54, *B3-1* was most similar to *GluB3-4* (94.3% identity), whereas both *B3-2* and *B3-3* were most similar to *GluB3-3* (>99% identities) (Table S3). Among the seven *Glu-D3* genes of Xiaoyan 54, *D3-1*, *D3-2*, *D3-3*, *D3-6* and *D3-7* displayed the highest nucleotide sequence identities to *GluD3-5* (99.9%), *GluD3-2* (99.4%), *GluD3-3* (99.8%), *GluD3-1* (99.7%) and *GluD3-4* (99.1%), respectively (Table S3). *D3-4* and *D3-5* both showed the highest nucleotide sequence identities (>97%) to *GluD3-6* (Table S3).

For the LMW-GS genes that shared higher than 90% nucleotide sequence identities in the above comparisons, their deduced protein products were generally of the same type (Table S3). Thus, *A3-1* and the two sequences represented by AB062868 and AJ293099 all specified m-type subunits (with an identical N-terminal domain sequence MDTSCIPGLERPW). *A3-2* and *Glu-A3d*, as well as *A3-4* and *Glu-A3b*, encoded i-type subunits. *B3-1* and *GluB3-4* coded m-type subunits, whereas *B3-2* and *GluB3-3* yielded s-type subunits. *D3-1*, *D3-2*, *D3-4*/*D3-5*, *D3-6* and *D3-7*, like their respective closest counterparts *GluD3-5*, *GluD3-2*, *GluD3-6*, *GluD3-1* and *GluD3-4*, all encoded m-type subunits, whereas *D3-3* and *GluD3-3* specified s-type subunits.

### Transcriptional profiles of LMW-GS genes during grain development

**Figure 3:**
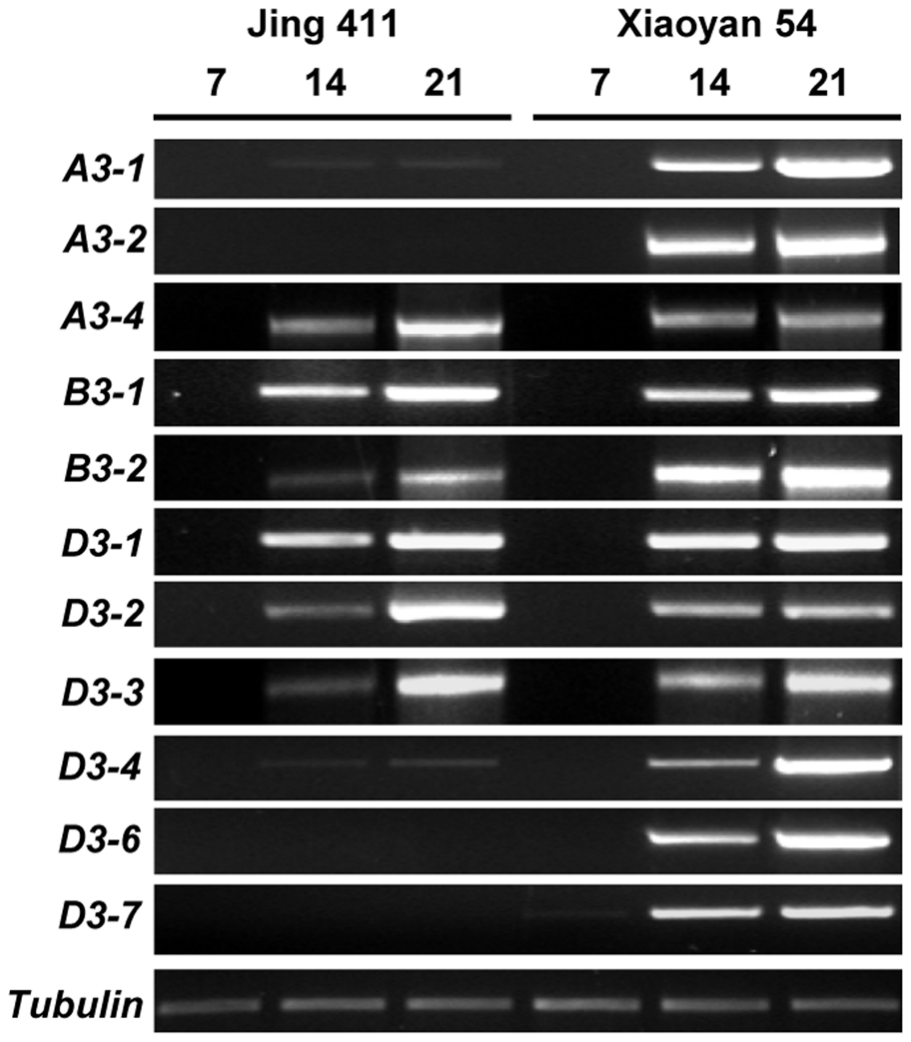
Transcriptional profiles of LMW-GS genes in developing grains of Jing 411 and Xiaoyan 54. Total RNA samples, extracted from developing grains at 7, 14 and 21 days post anthesis (DPA), were used for evaluating transcript levels of the LMW-GS gene members. The 11 LMW-GS genes were all highly transcribed in the developing grains of Xiaoyan 54 at 14 and 21 DPA. By contrast, in the developing grains of Jing 411, only 6 members (*A3-4*, *B3-1*, *B3-2*, *D3-1*, *D3-2*, *D3-3*) were highly transcribed at 14 and 21 DPA; two members (*A3-1*, *D3-4*) were weakly transcribed, and the transcripts of 3 members (*A3-2*, *D3-6*, *D3-7*) were undetectable by RT-PCR at the same time points. The amplification of wheat tubulin gene transcripts served as an internal control for normalizing the cDNA contents before PCR and checking the kinetics of thermamplification during PCR.

Further to the experiments described above, it was relevant and important to study the transcription profiles of LMW-GS genes in the developing seeds, and to compare the transcriptional patterns of these genes in wheat varieties differing in bread making quality. To achieve these goals, semiquantitative RT-PCR experiments were performed using gene-specific oligonucleotide primers (Table S1) designed for the 11 active LMW-GS genes of Xiaoyan 54. Figure 3 shows that the 11 members were all highly transcribed in the developing grains of Xiaoyan 54. By contrast, in the developing grains of Jing 411, the transcripts of *A3-2*, *D3-6* and *D3-7* were undetectable, and the relative transcript levels of *A3-1* and *D3-4* were both substantially lower than those in Xiaoyan 54, especially at 21 days post anthesis (DPA) (Figure 3). The data displayed in Figure 3 are representative of four independent RT-PCR experiments.

### Examination of LMW-GS gene sequences in Jing 411

The RT-PCR data described above indicated that there could be substantial differences between Xiaoyan 54 and Jing 411 in their LMW-GS genes. To investigate this possibility, genomic PCR experiments were conducted to amplify the nucleotide sequences of LMW-GS genes in Jing 411, using the primers derived from the 14 LMW-GS genes of Xiaoyan 54 (Table S1). Eleven LMW-GS gene sequences, which were alleles corresponding to *A3-1*, *A3-2*, *A3-4*, *B3-1*, *B3-2*, *B3-3*, *D3-1*, *D3-2*, *D3-3*, *D3-4* and *D3-5*, were amplified from Jing 411 (Accession numbers FJ907547, FJ907548, FJ907549, FJ907550, FJ907551, FJ907552, FJ755317, FJ755318, FJ755319, FJ755322, FJ755323). However, no PCR products were obtained from Jing 411 using the primers specific for *A3-3*, *D3-6* or *D3-7*. More detailed bioinformatic analysis of the LMW-GS gene sequences amplified from Jing 411 revealed that the *A3-1*, *A3-2* and *D3-4* alleles were pseudogenes due to the presence of premature stop codon or frame shift mutations in their coding regions. Like in Xiaoyan 54, *D3-5* was also a pseudogene in Jing 411, but *B3-3*, a pseudogene in Xiaoyan 54, possessed an intact ORF in Jing 411. Taken together, the above analysis suggested that the total number of inactive LMW-GS genes in Jing 411 was four (i.e., *A3-1*, *A3-2*, *D3-4* and *D3-5*), compared to three (i.e., *A3-3*, *B3-3* and *D3-5*) in Xiaoyan 54. The increased number of pseudogenes and the absence of *A3-3*, *D3-6* and *D3-7* alleles in Jing 411 were consistent with the findings of fewer transcribed LMW-GS genes in the developing grains of this variety in the foregoing RT-PCR assay (Figure 3). The amino acid sequence identities for the proteins deduced from the active LMW-GS genes of Xiaoyan 54 and Jing 411 were 91.51% for A3-4 alleles, 93.94% for B3-1 alleles, 92.44% for B3-2 alleles, 99.71% for D3-1 alleles, 100% for D3-2 alleles, and 92.43% for D3-3 alleles.

### Matching LWM-GS genes to their subunit products accumulated in the grains

**Figure 4:**
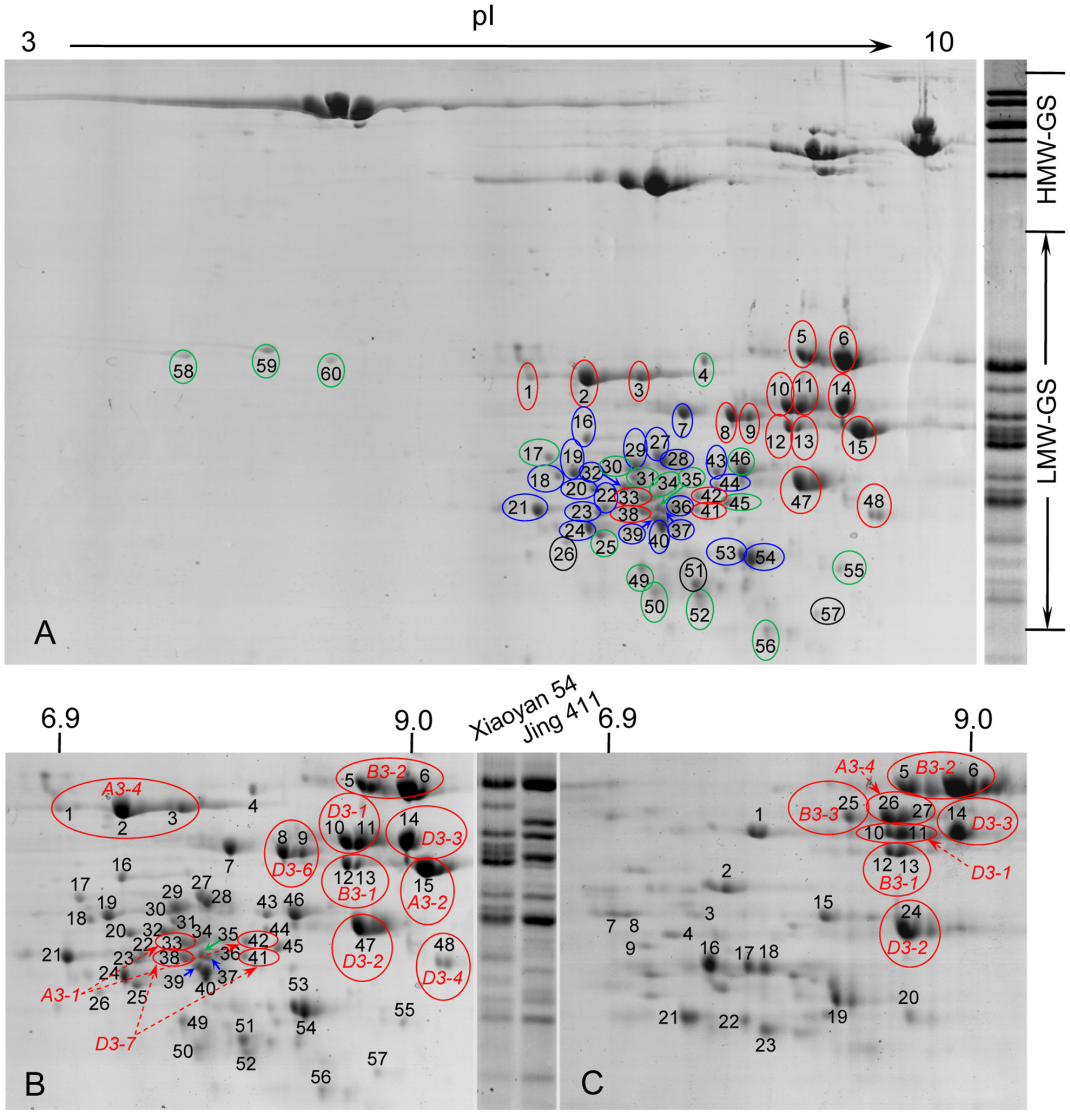
Identification of protein spots resolved by 2-DE of the glutenin samples. Glutenin fractions were prepared from mature grains, and used for 2-DE and subsequent MS analysis. For reason of space, the high molecular weight glutenin subunit protein spots are not shown. (A) Identification of 60 protein spots derived from the glutenin fraction of Xiaoyan 54. The LMW-GS and gliadin protein spots are circled in red and blue, respectively. Spots circled in black are known proteins with other molecular functions, whereas those in green are unknown proteins. The pI range in the 2-DE gel is shown at the top. Also shown (on the right side) is the one dimensional separation of the glutenin fraction of Xiaoyan 54. The data displayed are typical of four independent sets of 2-DE separation and MS analysis. (B, C) Comparisons of the LMW-GS protein species (circled in red) accumulated in the mature grains of Xiaoyan 54 (B) and Jing 411 (C). The names of the LMW-GS genes encoding the circled proteins are provided. For both varieties, the LMW-GS protein species were accompanied by some gliadin protein species and the proteins with other or unknown functions (represented by numbered but uncircled protein spots, Tables S4 and S6). The pI ranges in the 2-DE gels are displayed at the top. The one dimensional separation results of the glutenin fractions of the two varieties are also shown (labeled by Xiaoyan 54 and Jing 411, respectively). The data provided are representative of four independent sets of 2-DE separation and MS analysis.

The characterization of LMW-GS genes in the foregoing experiments, especially the finding of the differences in the spectrum of LMW-GS genes expressed in the two wheat varieties with differing bread making quality, made it necessary to identify the native protein products expressed from the active LMW-GS gene members. Towards this end, the glutenin fractions of the seed storage proteins of Xiaoyan 54 and Jing 411 were separated by two dimensional gel electrophoresis (2-DE), followed by analysis of the peptide mass fingerprints (PMFs) and mass spectra of excised protein spots using matrix-assisted laser desorption ionization-time of flight mass spectrometry (MALDI-TOF MS) and liquid chromatography tandem mass spectrometry (LC-MS/MS). For Xiaoyan 54, 60 major 2-DE spots in the region of LMW-GS proteins were marked (Figure 4A), excised, and analyzed. After bioinformatic analysis of PMFs and MS/MS spectra, 19 were found to be LMW-GS proteins (red circles), 21 were gliadins (blue circles), three were related to known proteins with other molecular functions (black circles), and 17 were unknown proteins (green circles) (Figure 4A and Table S4). More detailed comparisons were made between the peptide sequences obtained from the protein spots and the deduced amino acid sequences of the 11 active LMW-GS genes isolated in this work (Table S5). Based on these comparisons, the relationships between the analyzed protein spots and the proteins deduced from the cloned LMW-GS genes are summarized in Table 2 and Figure 4B. The phenomenon that two or three protein spots were matched to the deduced protein of a single LMW-GS gene (e.g., protein spots 1, 2 and 3 to *A3-4*) was observed previously, although the underlying reasons are still unclear [41], [61]. Among the 60 spots analyzed, none corresponded to the hypothetical polypeptides of *A3-3*, *B3-3* or *D3-5*, deduced by ignoring the premature stop codons (for *A3-3* and *D3-5*) or the partial coding sequence (for *B3-3*).

**Table 2:** Matching LMW-GS genes to the protein spots resolved by 2-DE through mass spectrometry analysis.

Gene	Bread wheat *Aegilops tauschii*			
	Xiaoyan 54	Jing 411	As91	Y207
*A3-1*	33, 42	–	NA c	NA
*A3-2*	15	–	NA	NA
*A3-3*	– a	--- b	NA	NA
*A3-4*	1, 2, 3	26, 27	NA	NA
*B3-1*	12, 13	12, 13	NA	NA
*B3-2*	5, 6	5, 6	NA	NA
*B3-3*	–	25	NA	NA
*D3-1*	10, 11	10, 11	1, 5, 6	1, 2
*D3-2*	47	24	8, 9 ,10	6
*D3-3*	14	14	7	5
*D3-4*	48	–	–	–
*D3-5*	–	–	–	–
*D3-6*	8, 9	---	2, 3, 12	3, 4
*D3-7*	38, 41	---	13	ND d

a No protein spots were identified for the 3 inactive members.

b No protein spots were found for the three members whose coding sequences were not amplified by genomic PCR.

c NA, not applicable.

d ND, not detected.

Of the 27 protein spots investigated for Jing 411, 11 (Figure 4C, circled in red) were characterized as LMW-GS proteins based on bioinformatic analysis of their PMFs and MS/MS spectra (Tables S6 and S7). The 11 spots were matched to seven active LMW-GS genes (Figure 4C, Tables 2, S6 and S7). Highly similar molecular mass and pI values were found between the protein spots derived from each of the five LMW-GS genes (*B3-1*, *B3-2*, *D3-1*, *D3-2* and *D3-3*) shared by Xiaoyan 54 and Jing 411 (Table 2, Figure 4B and 4C). The product of *A3-4* in Jing 411 (spots 26 and 27) had higher pI values than that in Xiaoyan 54 (spots 1, 2 and 3). Spot 25 in Jing 411 was matched to the deduced protein of *B3-3* (Figure 4C and Table 2), which was a pseudogene in Xiaoyan 54 (Table 1). Twelve spots (1, 2, 3, 4, 7, 8, 15, 16, 17, 18, 19 and 20) were gliadin proteins, three (21, 22 and 23) were known proteins with other molecular functions, and one (9) represented an unknown protein (Figure 4C, Tables S6 and S7). Among the 27 examined spots, none matched to the hypothetical polypeptides for *A3-1*, *A3-2*, *D3-4* or *D3-5*, translated with the premature stop codon or frame shift mutations ignored.

Collectively, these protein analysis data were consistent with the results of the foregoing RT-PCR assays, and showed that protein products of five LMW-GS genes (*A3-1*, *A3-2*, *D3-4, D3-6* and *D3-7*) accumulated in Xiaoyan 54 grains, were not present in the grains of Jing 411 (Figure 4 and Table 2). On the other hand, the protein product of *B3-3* was detected in the grains of Jing 411 but not those of Xiaoyan 54 (Figure 4 and Table 2).

### Analysis of LWM-GS genes and their products in *Ae. Tauschii*

The above experiments indicated the existence of five (in Jing 411) to seven (in Xiaoyan 54) LMW-GS genes at the *Glu-D3* locus of hexaploid wheat varieties. It was therefore interesting and necessary to investigate if the number of genes encoding typical LMW-GS proteins may also vary in different genotypes of *Ae. tauschii*. The LMW-GS genes and their native protein products in different *Ae. tauschii* genotypes (As91 and Y207) were analyzed. The genomic DNA samples of As91 and Y207 were each subject to PCR amplifications using several types of primers (Table S1), and the resultant products were cloned and sequenced. Based on nucleotide and amino acid sequence comparisons, seven distinct *Glu-D3* LMW-GS genes were identified for each genotype (Table 2) (Accession numbers from FJ755324 to FJ755337). The *D3-4* and *D3-5* alleles in As91 and Y207 were pseudogenes owing to the lack of intact ORF.

The LMW-GS fractions of As91 and Y207 were separated by 2-DE, followed by analysis using mass spectrometry as described above. Nineteen and 15 protein spots were detected in the region of LMW-GS proteins for As91 and Y207, respectively (Figure S3). For As91, 11 of the 19 analyzed spots were LMW-GS proteins, which matched separately to the deduced proteins of five LMW-GS genes (Figure S3A, Tables 2 and S8). Seven spots (4, 11, 14, 16, 17, 18 and 19) were gliadin proteins, whereas one (spot 15) resembled an avenin-like b precursor protein (Figure S3A, data not shown). For Y207, six of the 15 investigated spots were LMW-GS proteins, which corresponded separately to the deduced proteins of four LMW-GS genes (Figure S3B, Tables 2 and S9). Four spots (8, 9, 13 and 15) were identified to be gliadin proteins, one spot (14) was found to be an avenin-like b precursor protein, and the remaining 4 spots (7, 10, 11 and 12) represented unknown proteins (Figure S3B, data not shown). The product of *D3-7* was not detected in Y207 by 2-DE, probably due to its low expression level in this genotype.

### Effects of allelic *Glu-3* loci on Zeleny sedimentation value

The clear differences in the LMW-GS genes between Xiaoyan 54 and Jing 411 (as described above) provided a suitable system to investigate the effects of allelic *Glu-3* loci on bread-making quality. Our initial investigation detected major and consistent quantitative trait loci (QTLs) for Zeleny sedimentation value (ZSV) in the genomic regions containing the *Glu-A3* or *D3* loci (data not shown). We therefore performed a more detailed association analysis using 182 RILs derived from Xiaoyan 54 and Jing 411. ZSV was again chosen as the evaluation parameter based on our earlier QTL investigation. This choice was also supported by the published literature showing that ZSV correlated positively with gluten strength and bread volume [62]-[64], and that the genetic composition of *Glu-3* loci affected ZSV [65]-[68].

The ZSVs of the RILs and their parents were evaluated using grains produced in two growing seasons (2005-2006 and 2007-2008). The data from 2006 harvest were analyzed in more detail to investigate the genetic effects of allelic *Glu-3* loci on ZSV. The averaged ZSV of Xiaoyan 54 (40.00 ml) was significantly higher than that of Jing 411 (22.20 ml). The ZSVs of the 182 RILs varied from 9.22 to 61.45 ml, with a mean value of 32.4 ml. The ZSV means of the lines followed a normal distribution, and were thus suitable for association analysis. Eight main genotypes (*A3*_x_ *B3*_x_ *D3*_x_, *A3*_j_ *B3*_j_ *D3*_j_, *A3*_x_ *B3*_j_ *D3*_x_, *A3*_j_ *B3*_x_ *D3*_x_, *A3*_x_ *B3*_x_ *D3*_j_, *A3*_j_ *B3*_j_ *D3*_x_, *A3*_x_ *B3*_j_ *D3*_j_ and *A3*_j_ *B3*_x_ *D3*_j_, the subscripts x and j indicating allelic *Glu-3* loci from Xiaoyan 54 and Jing 411, respectively) were found among a total of 158 RILs (Table 3). Two genotypes (*A3*_x_ *B3*_x_ *D3*_x_ and *A3*_j_ *B3*_j_ *D3*_j_) resembled the Xiaoyan 54 or Jing 411 parents in their *Glu-3* complements. The *Glu-3* complements in the six recombinant genotypes (*A3*_x_ *B3*_j_ *D3*_x_, *A3*_j_ *B3*_x_ *D3*_x_, *A3*_x_ *B3*_x_ *D3*_j_, *A3*_j_ *B3*_j_ *D3*_x_, *A3*_x_ *B3*_j_ *D3*_j_ and *A3*_j_ *B3*_x_ *D3*_j_) were formed by inter-locus recombinations of the intact allelic *Glu-3* loci of Xiaoyan 54 and Jing 411. The number of active LMW-GS genes was calculated for each of the eight genotypes (Table 3).

**Table 3:** Statistical analysis of the Zeleny sedimentation values (means ± SD) of eight genotypes among 152 RIL lines derived from a cross between Xiaoyan 54 and Jing 411.

Genotype a	Numberof RILs	Number of active LMW-GS genes	Zeleny sedimentation value (ml)^ c^	P ≤ 0.05 b	P ≤ 0.01 b
*A3_x_B3_j_D3_x_*	21	12	39.73±10.42	a	a
***A3_x_B3_x_D3_x_***	12	11	37.49±3.70	ab	ab
*A3_j_B3_x_D3_x_*	19	9	34.42±6.24	abc	abc
*A3_x_B3_x_D3_j_*	9	8	33.54±7.15	bcd	abc
*A3_j_B3_j_D3_x_*	23	10	32.18±6.37	bcde	bc
*A3_x_B3_j_D3_j_*	15	9	31.37±5.07	cde	bc
*A3_j_B3_x_D3_j_*	24	6	28.16±6.88	de	c
***A3_j_B3_j_D3_j_***	35	7	27.66±9.04	e	c

a Parental genotypes are written in bold.

b Statistical analysis of the Zeleny sedimentation values (ZSVs) of the eight genotypes with ANOVA and Duncan LSR at two different confidence levels (P≤0.05 and 0.01). The ZSVs are labeled by different letters or letter combinations based on multiple statistical comparisons. No statistical significance exists between the ZSVs labeled by one or more identical letters.

The ZSVs (means ± SD) of the eight recombinant genotypes showed continuous variation (Table 3), consistent with previous findings showing that ZSV is controlled quantitatively by multiple chromosomal loci located on different wheat chromosomes [34], [65], [66]. Nevertheless, our statistical analysis showed that the averaged ZSV of *A3*_x_ *B3*_x_ *D3*_x_ was significantly higher than that of *A3*_j_ *B3*_j_ *D3*_j_. This demonstrated that in the genetic system analyzed here the differences in the composition of *Glu-3* loci had a significant effect on ZSV, which was in general agreement with the positive contributions of *Glu-3* loci to dough and bread-making qualities in published studies [13], [30], [32]-[34], [38], [56], [57], [59], [60], [64]. *A3*_j_ *B3*_j_ *D3*_x_, *A3*_x_ *B3*_j_ *D3*_j_ and *A3*_j_ *B3*_x_ *D3*_j_, each possessing a single *Glu-3* locus from Xiaoyan 54 (i.e., *D3*_x_, *A3*_x_ or *B3*_x_) in their *Glu-3* complements, did not differ from *A3*_j_ *B3*_j_ *D3*_j_ in ZSV (Table 3). This indicated that, at the single locus level, the *Glu-3* loci of Xiaoyan 54 did not differ significantly from those of Jing 411 in their effects on ZSV. By contrast, *A3*_x_ *B3*_j_ *D3*_x_, *A3*_j_ *B3*_x_ *D3*_x_ and *A3*_x_ *B3*_x_ *D3*_j_, each having two *Glu-3* loci from Xiaoyan 54 (i.e., *A3*_x_ and *D3*_x_, *B3*_x_ and *D3*_x_ or *A3*_x_ and *B3*_x_) in their *Glu-3* complements, exhibited the ZSVs significantly higher than that shown by *A3*_j_ *B3*_j_ *D3*_j_ (P≤0.05) (Table 3). The ZSV of *A3*_x_ *B3*_j_ *D3*_x_ remained significantly higher than that of *A3*_j_ *B3*_j_ *D3*_j_ at P≤0.01 (Table 3). Clearly, the combined effects of *A3*_x_ and *D3*_x_, *B3*_x_ and *D3*_x_ or *A3*_x_ and *B3*_x_ were significantly greater than those of *A3*_j_ and *D3*_j_, *B3*_j_ and *D3*_j_ or *A3*_j_ and *B3*_j_ in terms of generating relatively high ZSVs, with the *A3*_x_ and *D3*_x_ combination giving the strongest performance. Compared to the scenario described above, none of the six progeny genotypes yielded a ZSV that was significantly higher than that of *A3*_x_ *B3*_x_ *D3*_x_ (Table 3). Instead, the ZSVs of *A3_x_B3_j_D3_j_* and *A3_j_B3_x_D3_j_* were both significantly lower than that of *A3*_x_ *B3*_x_ *D3*_x_ (P≤0.05), and the ZSV of *A3_j_B3_x_D3_j_* remained notably lower than that of *A3*_x_ *B3*_x_ *D3*_x_ at P≤0.01 (Table 3). This confirmed that the combined effects of *B3*_j_ and *D3*_j_ or *A3*_j_ and *D3*_j_ were inferior to those of *B3*_x_ and *D3*_x_ or *A3*_x_ and *D3*_x_ in securing relatively high ZSVs, with the *A3*_j_ and *D3*_j_ combination resulting in a poorer performance.

Interestingly, the RILs having the *A3_x_B3_j_D3_x_* genotype in their *Glu-3* complements exhibited a considerably wider variation in ZSVs compared to the RILs having other *Glu-3* genotypes (Table 3). Although the averaged ZSV determined for *A3_x_B3_j_D3_x_* genotype did not differ significantly from that for *A3*_x_ *B3*_x_ *D3*_x_ (Table 3), there were four lines (among a total of 21 RILs) in the *A3_x_B3_j_D3_x_* genotype that consistently displayed the highest ZSVs (20-50% higher than the ZSV displayed by the superior parent Xiaoyan 54) in the tests conducted in two growing seasons. These data indicated that the *B3* locus of Jing 411 might interact positively with the *A3* and *D3* loci of Xiaoyan 54, leading to higher ZSVs in certain genetic backgrounds.

We also investigated the relationship between the number of active LMW-GS genes (i.e., the members possessing intact ORF) and the averaged ZSV among the eight genotypes. Regression analysis suggested a significant positive correlation between changes in the number of active LMW-GS genes and variations in ZSV among the eight genotypes (*r* = 0.903, p≤0.01, Figure S4). This positive correlation was well illustrated by the finding that the ZSVs of *A3_x_B3_j_D3_x_* and *A3_x_B3_x_D3_x_* (having 12 and 11 active LMW-GS genes, respectively) were consistently and significantly higher than those of *A3_j_B3_x_D3_j_* and *A3_j_B3_j_D3_j_* (possessing six and seven active LMW-GS genes, respectively) (Table 3). However, owing to the strong and continuous variation in ZSV, the genotypes with smaller differences in the number of active LMW-GS genes tended not to differ significantly from each other in this parameter. For example, *A3_j_B3_x_D3_x_*, *A3_x_B3_x_D3_j_*, *A3_j_B3_j_D3_x_* and *A3_x_B3_j_D3_j_* did not differ from each other in ZSV, although the number of active LMW-GS genes in the four genotypes varied from eight to ten (Table 3).

A highly significant correlation (*r* = 0.907, p≤0.01) was found between the averaged ZSVs of the eight main recombinant genotypes determined in 2005-2006 and those in 2007-2008, suggesting that the genetic effects of the different *Glu-3* genotypes on ZSV were relatively stable between the two years.

## Discussion

A number of studies have shown that the organization of orthologous *Glu-3* loci in wheat is highly complex [26], [27], [41], [42], [44]-[47]. Unlike previous investigations focused on single or a small number of BACs from individual *Glu-3* loci [26], [27], we used a genome wide approach, and identified and sequenced the BACs from all three *Glu-3* loci of a bread wheat variety. Fourteen unique LMW-GS genes were found in Xiaoyan 54, representing the highest number of LMW-GS genes characterized from a single bread wheat genotype so far. Furthermore, we carried out comparative analyses of the transcriptional and protein accumulation patterns of LMW-GS genes between Xiaoyan 54 and Jing 411, and performed an association study using the RIL population derived from a cross of the two varieties.

### Organization of LMW-GS genes at orthologous *Glu-3* loci

Firstly, we identified four and three LMW-GS genes for the *Glu-A3* (*A3-1* to *A3-4*) and *Glu-B3* (*B3-1* to *B3-3*) loci of Xiaoyan 54, respectively, by far the highest numbers of LMW-GS genes reported for the two loci from a single wheat genotype. Furthermore, we mapped *A3-1* to a location proximal to the centromere relative to *A3-2* to *A3-4*, and for the first time provided direct evidence for the presence, location and expression of a m-type subunit gene (*A3-1*) at *Glu-A3* in addition to those encoding i-type subunits. Previous studies indicated the existence of m-type subunit gene(s) at *Glu-A3*, and hypothesized the evolution of i-type members from m-type genes [26] (see also GenBank accessions AB062868 and AJ293099). The present work established a more solid basis for further investigation of this hypothesis in the future. Nucleotide sequence comparisons showed that the three i-type subunit genes isolated here exhibited high identities to only two (*Glu-A3b*, *Glu-A3d*) of the seven *Glu-A3* alleles reported for bread wheat varieties [47]. It is possible that the molecular diversity of *Glu-A3* LMW-GS genes is relatively high in the bread wheat gene pool, and different varieties may differ in the composition of *Glu-A3* LMW-GS genes. This may also explain the finding of two i-type subunit genes at the *Glu-A3* locus of Norin 61, but one active i-type subunit gene at the *Glu-A3* locus of Glenlea [41], [42]. Similarly, *B3-1*, *B3-2* and *B3-3* exhibited high nucleic sequence identities to only two (*GluB3-3*, *GluB3-4*) of the four *Glu-B3* LMW-GS genes defined previously [46], indicating that the number of *Glu-B3* LMW-GS genes in the whole bread wheat gene pool could be higher than that present in a given variety. A comparison of the *Glu-B3* LMW-GS genes between Xiaoyan 54 and Jing 411 (Table 2) indicated the presence of more s-type subunit than m-type subunit genes at this locus, consistent with the identification of three s-type, but only one m-type, subunit genes from a wide range of varietal backgrounds [46]. Two *Glu-B3* LMW-GS genes were reported for both Norin 61 and Glenlea [41], [42], but the total numbers of *Glu-B3* LMW-GS genes (including both active and inactive members) are still unknown for the two varieties.

Secondly, we identified and determined the relative genetic locations of seven LMW-GS genes (*D3-1* to *D3-7*) at *Glu-D3* in Xiaoyan 54. This number is identical to that described for the *Glu-D3* locus of three *Ae. tauschii* accessions (AUS18913, As91 and Y207, [43], this work) and Norin 61 [41]. Furthermore, the seven *Glu-D3* LMW-GS genes of Xiaoyan 54 each had one closely related counterpart (based on a nucleotide sequence identity above 97%) among the six *Glu-D3* LMW-GS genes isolated from diverse bread wheat varieties [44], [45]. In contrast, the number of detectable *Glu-D3* LMW-GS genes was five in Jing 411 (this work), and nine in Glenlea [42]. Collectively, these data strongly suggest that the number of *Glu-D3* LMW-GS genes could vary from five to nine, and that a large proportion of *Ae. tauschii* and bread wheat genotypes may contain seven LMW-GS genes at their *Glu-D3* locus. Our data (Table 2), as well as those published previously [41]–[45], all demonstrate that, within *Glu-D3*, the number of genes coding for m-type subunits is much greater than that encoding s-type subunits. Surprisingly, only m-type subunit genes were found at the *Glu-D3* locus in *Ae. tauschii* accession AUS18913 [43], indicating that the s-type subunit gene in this material might have been mutated.

Thirdly, the occurrence of one or more inactive LMW-GS gene members is common to all three orthologous *Glu-3* loci. For *Glu-A3*, the inactive members could originally encode i-type [26,27, this work] or m-type (i.e., the *A3-1* pseudogene in Jing 411, Table 2) subunits. For *Glu-B3*, the pseudogene members could mainly code for s-type subunits originally [26, this work]. For *Glu-D3*, pseudogenization appeared to affect mainly the members originally specifying m-type subunits [43, this work]. The s-type subunit gene at *Glu-D3* appeared to be expressed in diverse *Ae. tauschii* and bread wheat backgrounds [41,42,44,45, this work], except in *Ae. tauschii* accession AUS18913 [43]. The numbers of pseudogenes at allelic *Glu-A3*, *B3* or *D3* loci may vary substantially, which affects the total LMW-GS subunit species expressed in different bread wheat varieties. This is well illustrated by our comparative analysis of Xiaoyan 54 and Jing 411 (Table 2). There are more inactive LMW-GS gene members at the *Glu-A3* and *D3* loci of Jing 411 than in Xiaoyan 54, which is the main reason for fewer i- and m-type subunit species expressed in Jing 411.

### Recombination of LMW-GS genes and genetic distances of *Glu-3* loci

An important prerequisite for investigating the functions of the *Glu-3* loci through genetic analysis is to understand the recombination of LMW-GS genes and the relative genetic distances within the individual *Glu-3* loci. The identification of multiple LMW-GS genes within individual *Glu-3* loci allowed us to investigate these two aspects in Xiaoyan 54. The following points, summarized from the data of Xiaoyan 54, provide new insights into the recombination features of LMW-GS genes and genetic distances of the *Glu-A3*, *B3* and *D3* loci in bread wheat.

Firstly, for all three *Glu-3* loci, recombinations may occur unevenly between different LMW-GS genes. No recombination was detected among the three i-type subunit genes (*A3-2* to *A3-4*, Figure 2) within *Glu-A3*, despite the fact that *A3-2* and *A3-3* were separated by more than 70 kb and *A3-4* was not located in the BAC harboring *A3-2* and *A3-3* (Table 1). Within *Glu-B3*, recombination was not found between *B3-2* and *B3-3* (Figure 2). As the BAC clone containing *B3-2* and *B3-3* was not completely sequenced, it is currently not possible to deduce if physical distance between the two members plays a role in the lack of recombination between them. Within *Glu-D3*, recombination was not found for two pairs of LMW-GS genes (*D3-2* and *D3-3*, *D3-6* and *D3-7*) (Figure 2). In the case of *D3-2* and *D3-3*, the absence of recombinations is likely to be related to the very close physical distance (about 16 kb, Figure 1) between them.

Secondly, the genetic distance covered by LMW-GS genes differs substantially among the *Glu-A3*, *B3* and *D3* loci. In Xiaoyan 54, this value varied from 0.6 (*Glu-B3*) to 1.7 (*Glu-A3*) to 4.9 (*Glu-D3*) cM (Figure 2). The longest genetic distance was found for *Glu-D3*, correlating with the finding of more LMW-GS genes in this locus. Spielmeyer *et al*. [28] reported 2.7 cM for the three LMW-GS genes located on 1DS of *Ae. tauschii*. This distance is comparatively lower than the one obtained here, most likely due to the fact that fewer LMW-GS genes were mapped in their research.

Thirdly, based on the total genetic distance (4.9 cM) covered by LMW-GS genes and the minimum physical space (i.e., around 500 kb estimated from the total insert size of six BAC clones, Table 1) of *Glu-D3* in Xiaoyan 54, the average ratio of physical to genetic distance for this locus is probably higher than 100 kb per cM. This local recombination rate is considerably lower than that suggested for the *Glu-D3* locus of *Ae. tauschii*, which was about 20–50 kb/cM [28]. But our estimation is in line with recombination rates reported for some of the gene rich regions on wheat groups 1 and 5 chromosomes (118 kb/cM) [69], [70].

Concomitant to investigating the genetic features of LMW-GS genes, we also succeeded in placing polymorphic microsatellite markers in the vicinities of Xiaoyan 54 *Glu-3* loci. Because these microsatellites are generally used for constructing wheat chromosome linkage maps, their incorporations may facilitate the comparisons of the map positions of *Glu-3* LMW-GS genes of Xiaoyan 54 with those of other varieties. For example, in the composite map of common wheat 1B chromosome (http://wheat.pw.usda.gov/ggpages/map_summary.html), the genetic distance from the first *Glu-B3* marker to the microsatellite *Xgwm273.3* is 55 cM, which is comparable to the genetic distance covered from *B3-2*/*B3-3* to the same microsatellite shown in Figure 2B (64.9 cM). In the composite map of common wheat 1D chromosome (http://wheat.pw.usda.gov/ggpages/map_summary.html), the genetic distance from the only *Glu-D3* marker to the microsatellite *Xcfd61.1* is 44 cM, which is highly similar to the genetic distance from *D3-6*/*D3-7* to the same microsatellite marker (42.8 cM, Figure 2C). Importantly, we mapped and ordered six LMW-GS genes within the *Glu-D3* locus (compared to only one at the *Glu-D3* of the 1D composite map, see above), which provides a useful basis for investigating potential functional differences among the different *Glu-D3* LMW-GS genes in the future.

### Expression of LMW-GS genes in the grains

For more detailed understanding of the expression of LMW-GS genes in a given genotype, two key challenges are to determine the total numbers of active and inactive LMW-GS genes at individual *Glu-3* loci, and to match the active LMW-GS genes to their protein products accumulated in the seeds. In this work, we demonstrated that the combined use of gene cloning (through sequencing selected BAC clones and PCR amplification), expression profiling (via semiquantitative RT-PCR), and proteomics analysis (with 2-DE/MS analysis) represented an efficient strategy for dealing with the two challenges in bread wheat and for generating more systematic information linking active LMW-GS genes to their expressed products.

Among the 14 LMW-GS genes cloned from Xiaoyan 54, the 11 members with intact ORF were all highly transcribed in the developing grains and their proteins were present in the harvested seeds. Among the 11 LMW-GS genes isolated from Jing 411, the proteins translated from the seven LMW-GS genes with complete ORF were observed in the seeds, and evidence for high levels of transcription in the developing grains was obtained for six of the seven genes. Transcription of the *B3-3* allele was not evaluated in Jing 411 grains because it was originally found to be a pseudogene in Xiaoyan 54. Together, these data suggest that the LMW-GS genes with intact ORF are generally and highly expressed in developing grains and their products accumulate in mature wheat seeds, although exceptions do occur in certain *Ae. tauschii* accessions (see below). For either Xiaoyan 54 or Jing 411, the proteomics analysis did not find protein spots derived from the LMW-GS genes with interrupted ORF. This is in accordance with the very low transcript levels detected for the *A3-1* and *D3-4* alleles in Jing 411, the two genes possessing ORFs disrupted by premature stop codons. It is very likely that damaged ORF may also be responsible for the lack of expression of *A3-3* and *D3-5* in both Xiaoyan 54 and Jing 411, the absence of *B3-3* expression in Xiaoyan 54, and the lack of *A3-2* expression in Jing 411. No *D3-6* and *D3-7* transcripts were detected in the grains of Jing 411, which is in agreement with our failure to isolate the coding sequences of these two genes from this variety by genomic PCR amplifications. The two genes may not exist in Jing 411, or their coding regions may be damaged by transposon insertion. The disruption of LMW-GS ORF by transposon insertion was found previously for the *LMW.S4* gene in *Ae. tauschii* [43], and for the *B3-3* allele of Xiaoyan 54 in the present work.

The combination of gene cloning and proteomics analyses was also used successfully for assessing the LMW-GSs expressed in two different accessions of *Ae. tauschii* (As91, Y207). As in bread wheat, protein products were generally found for the LMW-GS genes with intact ORF, but not for those with disrupted ORF. One notable exception was that no protein product was detected by 2-DE/MS analysis for the *D3-7* allele in Y207, despite that it had an intact ORF. Further study is needed to investigate if the *D3-7* allele of Y207 is transcribed during grain development, or whether the protein translated from this allele is unstable in the endosperm cells. Among the seven *Glu-D3* LMW-GS genes in *Ae. tauschii* accession AUS18913, four had intact ORF, and were predicted to encode protein species with different molecular mass and pI values [43]. However, only three protein spots with the molecular mass and pI values of LMW-GSs were found in 2-DE analysis of polymeric gluten proteins, indicating that one of the four LMW-GS genes with intact ORF may not be expressed in the seeds. Together, our analysis and the one by Johal *et al.* [43] have yielded a more extensive understanding of the composition and expression of *Glu-D3* LMW-GS genes in *Ae. tauschii*, which is valuable for studying the structure and function of the homologous *Glu-D3* locus and gene members in bread wheat.

Protein spots representing gliadins were commonly found in our proteomics analyses of the glutenin fractions from either bread wheat or *Ae. tauschii* accessions. This is consistent with the observations made by previous investigators [21], [43]. There is now evidence for the incorporation of certain gliadins into the polymeric glutenin fraction although the potential effect of such gliadins on bread-making quality is not understood [21], [43]. Because of the existence of multiple genes coding for gliadins along with those encoding LMW-GS proteins in the chromosomal regions harboring *Glu-3* loci [26], an important task for the future will be to dissect the physical, genetic and functional relationships of the two types of seed storage proteins.

### Genetic mechanism involved in the function of *Glu-3* loci in bread-making quality

Although past breeding and QTL studies established that allelic LMW-GS alleles can differ significantly in their effects on bread-making quality, the molecular genetic mechanism responsible for the difference is still not well understood. In this work, the elucidation of the allelic variations of *Glu-3* loci between Xiaoyan 54 and Jing 411 and an understanding of the recombination characteristics of these genes enabled us to carry out an association analysis using the RILs derived from the two varieties. The knowledge gained provides new insights into the genetic mechanism behind the function of *Glu-3* loci, and may also have practical implications on the improvement of ZSV and bread-making quality.

Firstly, positive interactions between the orthologous *Glu-3* loci from the superior parent (Xiaoyan 54) may be important for the recombinant progenies to achieve relatively high ZSVs. However, the combinations of the strongest *Glu-3* alleles from the superior parent (for example, the *A3* and *D3* alleles of Xiaoyan 54) and certain alleles from the inferior parent (i.e., the *B3* allele of Jing 411) may yield the highest ZSV in certain genetic backgrounds. Secondly, the beneficial effects on ZSV, brought about either by the interaction among the *Glu-3* loci from the superior parent or via certain combinations of the *Glu-3* loci from both parents, are likely to be achieved through increasing the number of active LMW-GS genes contained in the *Glu-3* complement. In general, a higher number of active LMW-GS genes tends to lead to a higher ZSV. This may explain the presence of a number of RILs with very high ZSVs in the *A3_x_B3_j_D3_x_* genotype. It possessed the highest number of active LMW-GS genes (i.e., 12) owing to the combinations of the three *Glu-3* loci each of which contained more active LMW-GS genes than its allelic counterpart. However, the potential of *A3_x_B3_j_D3_x_* to give more elevated ZSV may be influenced by genetic backgrounds, which, in turn, may be responsible for the more extensive variation of ZSVs observed for the RILs with genotype *A3_x_B3_j_D3_x_*.

From the points discussed above, it appears that the total number of active LMW-GS genes contained in, and expressed from, *Glu-3* complements may largely determine the effect of LMW-GSs on ZSV and probably bread-making quality. Elite *Glu-3* alleles may generally be associated with the possession of higher numbers of active LMW-GS genes, and the combinations of elite alleles at all *Glu-3* loci (*A3*, *B3* and *D3*) may enhance the contribution of LMW-GSs to bread-making quality. Interestingly, the elite *Glu-3* locus allele, *LMW-2*, in durum wheat also harbors and expresses a higher number of active LMW-GS genes than the alternative allele [18], [21], [48], [50], [51], indicating similarity in the mechanism behind the function of LMW-GSs in end use quality in tetraploid and hexaploid wheat varieties.

In practical bread wheat improvement programs, the selection of recombinant progenies with the highest number of active LMW-GS genes in the *Glu-3* complement from breeding populations, combined with direct evaluation of their ZSVs, may increase the chance of obtaining lines with superior bread-making quality. In line with this suggestion, previous studies demonstrated that selection of higher sedimentation volume results in enrichment of the elite *LMW-2* locus in durum wheat breeding [71].

In summary, this work has substantially improved our understanding of the genomic organization and recombination of LMW-GS genes at orthologous *Glu-3* loci, the expression of LMW-GS genes in bread wheat and *Ae. tauschii*, and the genetic mechanism involved in the function of *Glu-3* loci in bread-making quality. The system characterized here may provide a useful model for further studies of the function and mechanism of LMW-GS genes, the physical structure of orthologous *Glu-3* loci, and the potential functional interaction between LMW-GSs and gliadins. The new insights and resources generated in this work may also accelerate the efficient use of these genes in further improvement of bread-making quality in wheat.

## Materials and Methods

### Plant materials and general molecular and bioinformatic methods

Winter-type bread wheat varieties Xiaoyan 54 and Jing 411 were used in this study. Xiaoyan 54 exhibited relatively high flour protein content (13.2%) and expressed five HMW-GS proteins (1Ax1, 1Bx14+1By15, 1Dx2+1Dy12). Jing 411 also expressed five HMW-GS proteins (1Ax2^*^, 1Bx7+1By8, 1Dx2+1Dy12) but showed a comparatively lower flour protein content (11.7%) [59], [60]. In baking tests, the loaf volume of Xiaoyan 54 was approximately 10% larger than that of Jing 411 [59], [60]. A recombinant inbred population containing 182 lines (F8 generation), developed from a cross between Xiaoyan 54 and Jing 411, was utilized for mapping LMW-GS genes and the subsequent association analysis (see below). Two *Ae. tauschii* accessions (As91 and Y207, obtained from the *Triticeae* Research Institute, Sichuan Agricultural University, China) were also used. Chinese Spring (CS) and the nulli-tetrasomic (NT) lines derived from CS (obtained from the National Bioresource Project, Japan) were employed for assigning positive BAC clones to individual group 1 chromosomes. The general molecular and bioinformatic methods used in this work are described in Methods S1.

### BAC library construction and screening and BAC sequencing

The main protocol for constructing the BAC library of Xiaoyan 54 was from Zhang *et al.* [72] and http://hbz.tamu.edu. The resultant library was screened by PCR using several pairs of primers specific for LMW-GS sequence (Table S1). Full details for BAC library construction, library screening and analysis of positive clones are given in Methods S1.

### Development of molecular markers, chromosomal location of BAC clones, and genetic mapping of LMW-GS genes

The molecular markers (Table S2) were mainly derived from LMW-GS genes or BAC end sequences. Specifically, the markers for tagging *A3-1*, *A3-2*/*A3-3*/*A3-4*, *B3-2*/*B3-3*, *D3-1*, *D3-4*, *D3-6*, or *D3-7* were developed from LMW-GS gene sequences, whereas those for *B3-1* or *D3-2*/*D3-3* were from microsatellite sequences (located adjacent to *B3-1* and *D3-2*, respectively). The marker for *D3-5* was developed from the reverse end sequence of the BAC clone D479-7-6. Details for using these markers to assign the positive BAC clones to group 1 chromosomes, or to map LMW-GS genes, are summarized in Table S2.

For mapping the LMW-GS genes of Xiaoyan 54 to wheat chromosomes, 182 recombinant inbred lines (RILs) derived from Xiaoyan 54× Jing 411 cross and the polymorphic markers listed Table S2 were used. Further details on mapping the LMW-GS genes and 23 previously published microsatellite markers along group 1 chromosomes are described in Methods S1.

### Evaluation of LMW-GS gene transcript level by semi-quantitative RT-PCR assay

The seedlings of Xiaoyan 54 and Jing 411 were vernalized at 4°C for four weeks, followed by growth in the greenhouse with a 16-h light/8-h dark photoperiod. Temperatures in the greenhouse were set at 23°C (day) and 14°C (night) before anthesis, and at 27°C (day) and 16°C (night) after flowering. The developing grains were harvested from the two varieties at 7, 14 and 21 days post anthesis, respectively, and were immediately used for total RNA extraction using Trizol reagent and the accompanying protocol (Invitrogen, CA, USA). Full details on RNA transcription, cDNA synthesis and semi-quantitative PCR are provided in Methods S1.

### Isolation of the coding sequences of LMW-GS genes from Jing 411 and *Ae. tauschii* accessions As91 and Y207

Primer pairs 1 to 17 (Table S1) were used to isolate LMW-GS gene sequences from Jing 411 by genomic PCR amplification. Primer pairs 2 to 7 and 13 to 17 (Table S1) were deployed for cloning the LMW-GS genes in *Ae. tauschii* accessions As91 and Y207. The resultant PCR products were sequenced commercially (Sun Bio-chem Technology, Beijing, China). Construction of LMW-GS gene nucleotide sequences was conducted as descried above.

### Glutenin extraction and 2-DE

Glutenin extraction was performed according to the method described by Singh *et al.* [73] with some modifications. Full details for preparing glutenin samples and their separation by 2-DE are described in Methods S1.

### Mass spectrometry analysis

Selected protein spots were carefully excised from gels. After destaining, the gel pieces were dehydrated with acetonitrile and dried. Digestion was performed in chymotrypsin reaction buffer containing 60 ng chymotrypsin (Sigma–Aldrich, MO, USA) at 30°C for 6 h. Finally, 8 µL trifluoroacetic acid extraction buffer [5% (v/v)] was added to stop the digestion. The peptides in the digested gel pieces were extracted, and the resulting supernatants were concentrated by freeze-drying. Further details on the analysis of peptide samples by MALDI-TOF-MS and LC-MS/MS are given in Methods S1.

### Evaluation of ZSV and association analysis

The RIL population and the two parents (Xiaoyan 54 and Jing 411) were grown for two crop seasons (2005–2006, 2007–2008) in the experimental farm of the Institute of Genetics and Developmental Biology, Chinese Academy of Sciences, Beijing. Standard winter wheat cultivation practices, including three irrigations and two applications of pesticides to control aphid infestation and powdery mildew, were followed. No major abiotic stresses (drought, hot and dry winds) were encountered. For each RIL and the parents, a sample of 500 g of grains (15.5% moisture) was milled using a Brabender Junior Mill (Brabender OHG, Duisburg, Germany) fitted with a 70GG sieve. Zeleny sedimentation values were measured for each line with three replicates according to Axford *et al.* [74].

The genetic compositions of *Glu-3* loci in the 182 RILs were determined using eight polymorphic markers (Table S2), leading to identification of eight main recombinant genotypes (*A3*_x_ *B3*_x_ *D3*_x_, *A3*_j_ *B3*_j_ *D3*_j_, *A3*_x_ *B3*_j_ *D3*_x_, *A3*_j_ *B3*_x_ *D3*_x_, *A3*_x_ *B3*_x_ *D3*_j_, *A3*_j_ *B3*_j_ *D3*_x_, *A3*_x_ *B3*_j_ *D3*_j_ and *A3*_j_ *B3*_x_ *D3*_j_) among the RILs. Several rare *Glu-3* genotypes (in a total of 24 RILs) resulting from intra-locus recombinations were also found, but were not included in the association analysis owing to the very low number of RILs per genotype. Based on the analysis of the allelic *Glu-3* loci of Xiaoyan 54 and Jing 411, the number of active LMW-GS genes was summarized for each of the eight recombinant progeny genotypes. The averaged ZSVs determined for the eight main recombinant genotypes during the two seasons were significantly correlated (*r* = 0.907, p≤0.01). For statistical analysis, t-tests were performed using the T-TEST procedure of SAS 10.0 (SAS Institute Inc., North Carolina, USA).

## Supporting Information

Methods S1 (0.07 MB DOC)

Figure S1 Southern blot hybridization analysis of positive BAC clones using a probe specific for LMW-GS genes.
(0.03 MB PDF)

Figure S2 Alignment of the amino acid sequences deduced from active LMW-GS genes of Xiaoyan 54.
(0.02 MB PDF)

Figure S3 Identification of protein spots resolved by 2-DE of glutenin samples from As91 and Y207.
(0.04 MB PDF)

Figure S4 Significant and positive correlation between number of active LMW-GS genes and mean ZSV.
(0.02 MB PDF)

Table S1 Oligonucleotide primers used for LMW-GS gene cloning and expression profiling experiments.
(0.02 MB PDF)

Table S2 Oligonucleotide primers used in BAC clone chromosomal localization and LMW-GS gene genetic mapping experiments.
(0.02 MB PDF)

Table S3 Nucleotide sequence identities of the 14 LMW-GS genes of Xiaoyan 54 to previously reported Glu-A3, B3 or D3 LMW-GS alleles/genes [44]-[46], [47].
(0.03 MB PDF)

Table S4 Characterization of protein spots resolved by 2-DE of the glutenin fraction of Xiaoyan 54 with MALDI-TOF-MS and LC-MS/MS analyses.
(0.04 MB PDF)

Table S5 Matching LMW-GS protein spots resolved by 2-DE to the proteins predicted from the cloned active LMW-GS genes using the mass spectragraphs generated by LC-MS/MS analysis in Xiaoyan 54.
(0.05 MB PDF)

Table S6 Characterization of the protein spots resolved by 2-DE of the glutenin fraction of Jing 411 with MALDI-TOF-MS and LC-MS/MS analyses.
(0.03 MB PDF)

Table S7 Matching LMW-GS protein spots resolved by 2-DE to proteins predicted from the cloned active LMW-GS genes using mass spectragraphs generated by LC-MS/MS analysis in Jing 411.
(0.05 MB PDF)

Table S8 Matching LMW-GS protein spots resolved by 2-DE to proteins predicted from cloned active LMW-GS genes using the mass spectragraphs generated by LC-MS/MS analysis in Ae. tauschii accession As91.
(0.05 MB PDF)

Table S9 Matching LMW-GS protein spots resolved by 2-DE to proteins predicted from cloned and active LMW-GS genes using mass spectra generated by LC-MS/MS analysis in Ae. tauschii accession Y207.
(0.03 MB PDF)
