# Supplementary material for: New Insights into the Organization, Recombination, Expression and Functional Mechanism of Low Molecular Weight Glutenin Subunit Genes in Bread Wheat
Source: PLoS One. 2010 Oct 21;5(10):e13548. doi: 10.1371/journal.pone.0013548 (PMC2958824; doi:10.1371/journal.pone.0013548)
Supplement: Methods S1 — (0.07 MB DOC) [file pone.0013548.s001.doc]

**Methods S1**

**General molecular and bioinformatic methods**

General methods for DNA, RNA and protein manipulations, cDNA synthesis, DNA cloning, Southern blot hybridization, and optimization of PCR conditions were adopted from Sambrook and Russell [1]. High-fidelity Taq DNA polymerases Advantage® 2 polymerase Mix (Clontech, CA, USA) and LA-Taq (TaKaRa, Dalian, China) were used for all PCR amplifications. The nucleotide sequences of LMW-GS genes were each constructed with the sequencing information from at least three positive clones derived from independent PCR cloning experiments. Nucleotide sequence comparisons were carried out using BLAST (Basic Local Alignment Search Tool) algorithms [2]. FGENESH (http://www. softberry.com/nucleo.html) and GENESCAN (http://genemark.mit.edu/GENESCAN.htm) were used for gene prediction during the annotation of BAC sequences.

**BAC library construction, library screening and analysis of positive clones**

Seeds of Xiaoyan 54 were germinated and grown in a greenhouse for three weeks. The leaves were harvested and high molecular weight (HMW) nuclear DNA was extracted following the protocol described by [3]. Subsequently, the HMW DNA was partially digested with the restriction enzymes *Hind*III or *Bam*HI, and large DNA fragments (100 to 250 kb) were purified and ligated into the BAC vectors pIndigo BAC-5 (*Hind*III-Cloning Ready) or pIndigo BAC-5 (*Bam*HI- Cloning Ready (Epicentre Biotechnologies, Madison, USA). The ligation products were introduced into *Escherichia coli* strain DH10B by electroporation and grown overnight on LB agar plates containing 12.5 mg/L chloramphenicol. Positive BAC clones were picked and stored in 384-titre plates (5 clones per well) at -80℃. Seven pairs of PCR primers (Table S1) were used for screening the BAC library. LMW-I, LMW-S and LMW-M primer pairs were specific for i-type, s-type and m-type LMW-GS sequences, respectively, whereas LMW-Z1 and LMW-Z2 recognized all three types of LMW-GS genes. The methods for PCR screening and obtaining BAC end sequences were described previously [4]. The assembly of BAC contigs from positive clones was based on *Hind*III, *Not*I, *Sal*I, and *Not*I + *Sal*I restriction enzyme digestion patterns and comparisons of BAC end sequences. Representative BAC clones were sequenced commercially (BGI Life Technology Company, Beijing, China). The resultant BAC sequence data were examined for not only LMW-GS genes but also several known markers and genes (i.e., *SFR159*, *WHS179*, *Pm3* analog, *LrK10*) previously found to reside in the vicinities of *Glu-3* loci [5-7]. For A1056-11-5 and D1220-5-2 that were not successfully sequenced, the presence or absence of *SFR159*, *WHS179*, *Pm3* analog and *LrK10* sequences in their inserts was checked by PCR amplification using specific primers (Table S1).

**Mapping LMW-GS genes and 23 microsatellite markers along group 1 chromosomes**

The 14 LMW-GS genes and a set of 23 previously published microsatellite markers (*Xbarc8, Xbarc119.2, Xbarc128.1, Xbarc128.3, Xbarc152.1, Xbarc152.2, Xcfa2153, Xcfa2158.1, Xcfd56.2, Xcfd58, Xcfd61.1*, *Xcfd61.2, Xgwm273.2, Xgwm273.3, Xswes98, Xswes11*, *Xswes131, Xswes215, Xwmc221.1, Xwmc329.1, Xwmc329.2, Xwmc336* and *Xwmc432*) were mapped. The *Xgwm* markers were described by [8], whereas the *Xswes* series was from [9]. The *Xbarc*, *Xcfa*, *Xcfd* and *Xwmc* markers were described in http://www.wheat.pw.usda.gov. The markers were amplified by PCR using genomic DNA samples extracted from the parents and RILs as templates. For the microsatellite based markers, the protocols for PCR amplification and product separation by denaturing urea-polyacrylamide gel electrophoresis were adopted from publications [8-12]. For the markers developed from LMW-GS genes, the methods described by Martins-Lopes *et al.* [13] were followed for PCR amplification and product separation via single-strand conformation polymorphism gels. Segregating marker bands were scored as either A (identical to the one in Xiaoyan 54) or B (identical to that in Jing 411). The software Mapmaker/Exp ver. 3.0 [14] was used to construct linkage maps with minimum threshold LOD scores of 5.0 and maximum recombination fractions of 0.5. The Kosambi map function [15] was employed to convert the recombination frequencies to marker distances in centiMorgan (cM), and the order of markers was established using three point analysis and published positions of the mapped microsatellite markers. The linkage map was generated by MapDraw [16].

**RNA transcription, cDNA synthesis and semi-quantitative PCR**

Total RNA was dissolved in DEPC-treated water, followed by the removal of potential genomic contamination using RNase-free DNAase (Promega, Madison, USA). Reverse transcription was conducted using M-MLV reverse transcriptase (Promega, Madison, USA) using 2 μg total RNA from each sample. The cDNA contents in the different reverse transcription mixtures were standardized by amplifying wheat *tubulin* gene transcripts, followed by semi-quantitative PCR assays. Specific primers were designed for nine of the 11 active LMW-GS genes (i.e., those possessing intact ORF) of Xiaoyan 54 (Table S1). A single primer pair was used to amplify the transcripts of another two active members (*B3-2* and *D3-3*), yielding fragments with different sizes (800 bp for *B3-2*, and 684 bp for *D3-3*), and allowing separate evaluations of the transcript levels of the two genes. The PCR products were examined by ethidium bromide-DNA gel electrophoresis and documented using a CCD camera. The semi-quantitative assay was repeated four times with total RNA samples extracted from separate batches of developing grains (collected at the same time points described above), and revealed highly similar transcriptional patterns.

**Preparing glutenin samples for separation by 2-DE**

Gliadins in the endosperm half of a single wheat kernel were extracted (60 min) in 50% isoproponal at 65℃. After repeating this extraction three times, glutenins were extracted from solution [50% isoproponal, 0.08M Tris-HCl, pH 8.0, and 1% (w/v) DTT] at 65℃ lasting 1 hour. After centrifugation, the supernatant was precipitated with 4 volumes of cold acetone at -20℃ overnight. Subsequently, the proteins were collected by centrifugation in a pre-cooled rotor at 15000 *g* and 4℃ for 15 min. The protein pellet was washed three times with cooled acetone containing 0.1 % (w/v) DTT, followed by freeze-drying. The pellet was then dissolved in isoelectric focusing (IEF) sample extraction solution [containing 8 M urea, 50 mM DTT, 4% (w/v) CHAPS (3-[(3-Cholamidopropyl)dimethylammonio]-1-propanesulfonate), and 0.5% (v/v) IPG (Immobilized pH gradient) buffer (pI 3-10)]. Finally, the protein samples were quantified using a 2D Quant Kit (GE Healthcare, Buckinghamshire, UK) according to the manufacturer’s instructions.

IEF was performed using 24 cm immobilized pH gradient strips (GE Healthcare, Buckinghamshire, UK), with a linear pH gradient from 3 to 10. Focusing was achieved using the Ettan IPGphorII IEF system. For strip rehydration, a volume equivalent to 500 μg of each protein extract was added to the rehydration solution (8 M urea, 20 mM DTT, 2% (w/v) CHAPS, 0.5% (v/v) IPG buffer (pI 3-10), and 0.0002% v/v bromophenol blue), bringing the final volume to 450 µL. After active rehydration, IEF was performed at 200 V for 1.5 h, 500 V for 1.5 h, 1000 V for 2 h, Gradient 8000 V for 3 h, and 8000 V for 8 h. After IEF, strips were equilibrated for 15 min in equilibration buffer (6 M urea, 0.375 M Tris-HCl (pH 8.8), 2% (w/v) SDS, 30% (v/v) glycerol) containing 2% (w/v) DTT, followed by another 15 min in fresh equilibration buffer containing 2.5% (w/v) iodoacetamide. Separation in the second dimension was conducted at 15℃ using SDS PAGE [12.5% (w/v) acrylamide and 0.33% (w/v) bisacrylamide]. After the electrophoresis, the gels were stained with colloidal Coomassie brilliant blue G250 according to Candiano *et al.* [17], and destained with distilled water. Molecular mass markers were loaded onto the second dimension. The destained gels were scanned using a UMAX PowerLook 1120 color scanner. The collected images were analyzed using ImageMaster^TM^ 2D Platinum software (Version 5.0, GE Healthcare, Buckinghamshire, UK).

**Analysis of peptide samples by MALDI-TOF-MS and LC-MS/MS**

The peptide extract (1 µL) from each chymotryptic digestion was crystallized in 1 µL CHCA (α-Cyano-4-hydroxycinnamic acid, Sigma–Aldrich, MO, USA) matrix solution on the matrix-assisted laser desorption/ionization–time of flight (MALDI-TOF) carrier plate. The acquisition of the MALDI spectra was performed with a Bruker Autoflex mass spectrometer (Bruker Daltonics, MA, USA). Positively charged ions in the m/z range 900-3500 were analyzed. For obtaining accurate peptide sequence information, liquid chromatography tandem MS (LC-MS/MS) analysis was conducted with a LCQ deca XP plus ion trap mass spectrometer (Thermo Finnigan, MA, USA). The chymotryptic peptides from each digested sample were diluted with 15 µL of 0.5% (v/v) formic acid for LC-MS/MS, with the resultant spectra collected according to the manufacturer’s instructions.

The peptide mass fingerprints generated by MALDI-TOF-MS were searched against the NCBInr (other green plants) database using the program Biotools 2.1 (Bruker Daltonics, MA, USA) and the Mascot program available on the internet. The criteria for positive identification by MALDI-TOF spectra included five or more matching peptides (with 0.15 to 0.5 Da peptide mass tolerance), 1 missed cleavage, and at least 15% sequence coverage. The LC-MS/MS spectra were analyzed with Bioworks 3.1 software, using the protein sequences available in NCBI (before 2009-5-20) and deduced amino acid sequences of the LMW-GS genes cloned in this work.

**References**

1. Sambrook J, Russell DW (2001) Molecular cloning: a laboratory manual. Cold Spring Harbor Laboratory Press, Cold Spring Harbor, NY.

2. Altschul S, Madden TL, Schaeffer AA, Zhang J, Zhang Z, et al. (1997) Gapped BLAST and PSI-BLAST: a new generation of protein database search programs. Nucleic Acids Res 25: 3389-3402.

3. Zhang HB, Choi S, Woo SS, Li Z, Wing RA (1996) Construction and characterization of two rice bacterial artificial chromosome libraries from the parents of a permanent recombinant inbred mapping population. Mol Breed 2: 11-24.

4. Marek LF, Mudge J, Darnielle L, Grant D, Hanson N, et al. (2001) Soybean genomic survey: BAC-end sequences near RFLP and SSR markers. Genome 44: 572-581.

5. Spielmeyer W, Moullet O, Laroche A, Lagudah ES (2000) Highly recombinogenic regions at storage protein loci from chromosome 1DS of *Aegilops tauschii*, the D genome donor of wheat. Genetics 155: 361-367.

6. Wicker T, Yahiaoui N, Guyot R, Schlagenhauf E, Liu ZD, et al. (2003) Rapid genome divergence at orthologous low molecular weight glutenin loci of the A and A^m^ genomes of wheat. Plant Cell 15: 1186-1197.

7. Yahiaoui N, Srichumpa P, Dudler R, Keller B (2004) Genome analysis at different ploidy levels allows cloning of the powdery mildew resistance gene *Pm3b* from hexaploid wheat. Plant J 37: 528-538.

8. Röder MS，Korzun V, Wendehake K, Plaschke J, Tixier MH, et al. (1998) A microsatellite map of wheat. Genetics 149: 2007-2023.

9. Chen HM, Li LZ, Wei XY, Li SS, Lei TD, et al. (2005) Development, chromosome location and genetic mapping of EST-SSR markers in wheat. Chinese Sci Bull 50: 2328-2336.

10. Somers DJ, Isaac P, Edwards K (2004) A high-density wheat microsatellite consensus map for bread wheat (*Triticum aestivum* L.). Theor Appl Genet 109: 1105-1114.

11. Singh K, Ghai M, Garg M, Chhuneja P, Kaur P, et al. (2007) An integrated molecular linkage map of diploid wheat based on a *Triticum boeoticum* x *T. monococcum* RIL population. Theor Appl Genet 115: 301-312.

12. Zhang W, Chao S, Manthey F, Chicaiza O, Brevis JC, et al. (2008) QTL analysis of pasta quality using a composite microsatellite and SNP map of durum wheat. Theor Appl Genet 117: 1361-1377.

13. Martins-Lopes P, Zhang H, Koebner R (2001) Detection of single nucleotide mutations in wheat using single strand conformation polymorphism gels. Plant Mol Biol Rep 19: 159-162.

14. Lander ES, Green P, Abrahamson J, Barlow A, Day MJ, et al. (1987) Mapmaker: an interactive computer package for constructing primary genetic linkage maps of experimental and natural populations. Genetics 121: 174-181.

15. Kosambi DD (1944) The estimation of map distance from recombination values. Ann Eugen 12: 172-175.

16. Liu RH, Meng JL (2003) MapDraw: a microsoft Excel macro for drawing genetic linkage maps based on given genetic linkage data. Hereditas 25: 317-321.

17. Candiano G, Bruschi M, Musante L, Santucci L, Ghiggeri GM, et al. (2004) Blue silver: a very sensitive colloidal Coomassie G-250 staining for proteome analysis. Electrophoresis 25: 1327-1333.
