# Supplementary material for: New Insights into the Organization, Recombination, Expression and Functional Mechanism of Low Molecular Weight Glutenin Subunit Genes in Bread Wheat
Source: PLoS One. 2010 Oct 21;5(10):e13548. doi: 10.1371/journal.pone.0013548 (PMC2958824; doi:10.1371/journal.pone.0013548)
Supplement: Figure S1 — Southern blot hybridization analysis of positive BAC clones using a probe specific for LMW-GS genes. (0.03 MB PDF) [file pone.0013548.s002.pdf]

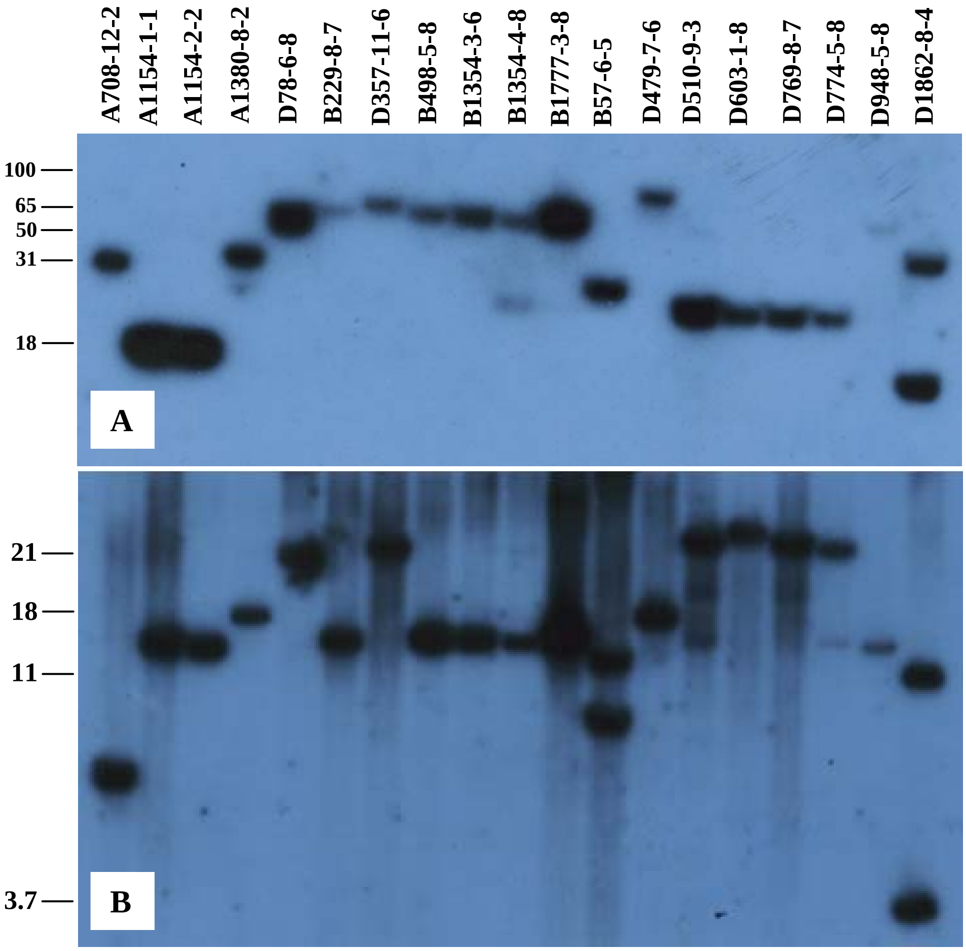

Figure S1. Southern blot hybridization analysis of 19 positive BAC clones using a radioactive probe specific for LMW-GS gene coding sequence. (*A*) Hybridizing band pattern obtained after digesting BAC DNA samples with the restriction enzyme *Not*I. (*B*) Hybridizing band pattern scored by digesting BAC DNA samples with the restriction enzymes *Not*I and *Sal*I. The DNA size markers (kb) are shown on the left side of the graph. The data displayed are typical of three separate hybridization experiments.
