# Supplementary material for: New Insights into the Organization, Recombination, Expression and Functional Mechanism of Low Molecular Weight Glutenin Subunit Genes in Bread Wheat
Source: PLoS One. 2010 Oct 21;5(10):e13548. doi: 10.1371/journal.pone.0013548 (PMC2958824; doi:10.1371/journal.pone.0013548)
Supplement: Figure S2 — Alignment of the amino acid sequences deduced from active LMW-GS genes of Xiaoyan 54. (0.02 MB PDF) [file pone.0013548.s003.pdf]

```
                   |<--  Sig  -->|<-N-ter->|<----------------------------------------- Rep ----------------------------------------
A3-1.pro           MKTFLIFALLAVVATSAIAQMDTSCIPGLERPWQQQPLPPQQT----------------------FPQQ---------PPFSQQQQ--QQQQQ----------------PFP--QQPSFS 69
D3-7.pro           MKTFLIFALIAVVATSAIAQMETSCISGLERPWQQQPLPPQQS----------------------FSQQ---------PPFSQQ------QQQ----------------PLP--QQPSFS 65
D3-4.pro           MKTFLIFALLAVVATSAIAQMETSCIPGLERPWQQQPLQQKET----------------------FPQQ---------PPSSQQ-------QQ----------------PFP--QQPPFL 64
D3-6.pro           MKTFLIFALLAIAATSAIAQMETSRVPGLEKPWQQQPLPPQQQPPCSQQQ-------------QPFPQQQQPIIILQQSPFSQQQQPVLPQQQ----------------PVIILQQPPFS 91
B3-1.pro           MKTFLIFALLAVAATSAIAQMETSHIPSLEKPLQQQPLPLQQILWYQQQQP-------IQQQPQPFPQQ---------PPCSQQ------QQP----------------PLSQQQQPPFS 82
D3-1.pro           MKTFLIFALLAVAATSAIAQMETSHIPGLEKPSQQQPLPLQQILWYHQQQP-------IQQQPQPFPQQ---------PPCSQQ------QQP----------------PLSQQQQPPFS 82
B3-2.pro           MKTFLIFALLAVAATSAIAQMENSHIPGLERPSQQQPLPPQQTLSHHHQQQP------IQQQPHQFPQQQPCSQQQQQPPLSQQQQPPFSQQQQPPFSQQQQPVLPQQPSFSQQQLPPFS 114
D3-3.pro           MKTFLIFALLAVAATSAIAQIENSHIPGLEKPSQQQPLPLQQTLSHHQQQQP------VQQQPQPFPQQQPCSQQQQ-PPLSQQQQPPFSQQQ-PPFSQQQQ------PSFSQQQQPPFS 106
D3-2.pro           MKTFLVFALLAVAATSAIAQMETRCIPGLERPWQQQPLPPQQT----------------------FPQQ----------PLFSQQQQLFPQQ----------------PSFSQQQ-PPFW 71
A3-2.pro           MKTFLVFALLALAAASAVAQ----------ISQQQQPPLFSQQQQPPFSQQQQPPFSQQQQSPFSQQQQPPFSQQQQPPFSQQ-PPISQQQQPQFLQQQQQ-------PPFSQQQQPPFS 102
A3-4.pro           MKTFLVFALLALAAARAVAQ----------ISQQQQQPPFSQQQQPPFSQQQQPPFSQQQQSPFSQQQEQQQQPPFLQQQQPPFSQQ-PPISQQQQPPFSQEQQ----PPFSQQQQPPFS 105

                   -------------------------------------------------------------------------------------------------------------------------
A3-1.pro           QQQ-------PPFSQQQPILPQG----------------PPFSQ---------------QTQPVLPQQ----------------SPFSQQ-------------------QQ------LIL 110
D3-7.pro           QQQ-------PPFSQQQPILSQQ----------------PPFSQ---------------QQQPVLPQQ----------------SPFSQQ-------------------QQ------LVL 106
D3-4.pro           QQQ-------PSFSQQ-----------------------PLFSQ---------------KQQPVLPQQ----------------PAFSQQ-------------------QQ------TVL 98
D3-6.pro           QQQQPVLPQQPPFSQQQQQQQQQ----------------PPFSQ---------------QQQPVLPQQ----------------PPFSQQ-------------------QQ------PPF 140
B3-1.pro           QQQ-------PPFSQQQQPILPQQ---------------PPFSQQQQQ--------FPQQQQPLLPQQ----------------PPFSQQQPPFSQQ------------QQ------PPF 139
D3-1.pro           QQQ-------PPFSQQELPILPQQ---------------PPFSQQQQPQFSQQQQPFPQQQQPLLLQQ----------------PPFSQQRPPFSQQQ-----------QQPVLPQQPPF 153
B3-2.pro           QQQQ------PPFSQQQQPVLPQQPSFSQQQLPPFSQQLPPFSQ---------------QQQPVLPQQ----------------PPFSQQQLPPFSQQLPPFSQQ----QQ------PVL 187
D3-3.pro           QQQ-------PPFSQQQQPVIPQQPSFSQQQLPPFSQQQPPFSQ---------------QQQPVLPQQ----------------PPFSQQ-------------------QQ------PIL 163
D3-2.pro           QQQ-------PPFSQQQ-PILPQQ---------------PPFSQ---------------QQQLVLPQQ----------------PPFSQQ-------------------QQ------PVL 112
A3-2.pro           QQQQ------PPYSQQQQPPFSQQ-------------QQPPFSQ---------------QQQPPFSQQQQQQQ-----------PPFTQQ-------------------QQPPFSQQPPI 158
A3-4.pro           QQQQ------PPYSQQQQPPFSQQ-------------QQPPFSQ---------------QQQPPFSQQQQQQQQQQQQPFTQQQPPFSQQPPISQQQQQQQQQQQPFTQQQPPFSQQPPI 191
                   ---------------------------------------------->|<----------------------------- C-ter ------------------------------------
A3-1.pro           P---------------PQQQQQ-LPQQ--------------QISIVQPSILQQLNPCKVFLQQQCSPVVMPQRLARSQMWQQSSCHVMQQQCCQQLSQIPEQSRYDAIRAITYPIILQEQ 200
D3-7.pro           P---------------PQQQQQQLVQQ--------------QIPIVQPSVLQQLNPCKVFLQQQCSPVAMPQRLARSQMWQQSSCHVMQQQCCQQLQQIPEQSRYEAIRAI----ILQEQ 193
D3-4.pro           PQQP-----------AFPQQQHQQLLQQ-------------QIPIVHPSILQQLNPCKVFLQQQCSPVAMPQHLARSQMWQQSSCNVMQQQCCQQLPRIPEQSRYEAIRAIIFSIILQEQ 194
D3-6.pro           SQQQQP----SSQQPPFPQQHQQFPQQ--------------QIPVVQPSVLQQLNPCKVFLQQQCSHVAMSQRLARSQMWQQSSCHVMQQQCCQQLPQIPEQSRSEAIRAIVYSIILQEQ 242
B3-1.pro           SQQQQQ-PILLQQPPFSQHQQPVLPQQ--------------QIPSVQPSILQQLNPCKVFLQQQCSPVAMPQSLARSQMLWQSSCHVMQQQCCRQLPQIPEQSRYDAIRAIIYSIVLQEQ 244
D3-1.pro           SQQQQQQPILPQQPPFSQHQQPVLPQQ--------------QIPYVQPSILQQLNPCKVFLQQQCSPVAMPQSLARSQMLWQSSCHVMQQQCCQQLPRIPEQSRYDAIRAIIYSIVLQEQ 259
B3-2.pro           P-QQPPFS---------QQQQQPILPQQPPFSQQQQPVLLQQQIPFVHPSILQQLNPCKVFLQQQCSPVAMPQSLARSQMLQQSSCHVMQQQCCQQLPQIPQQSRYEAIRAIVYSIILQEQ 298
D3-3.pro           P-QQPPFS---------QQQQQPVLPQQ--------------QIPFVHPSILQQLNPCKVFLQQQCSPVAMPQSLARSQMLQQSSCHVMQQQCCQQLPQIPQQSRYEAIRAIIYSIILQEQ 260
D3-2.pro           PPQQSPFP---------QQQQHQQLVQQ--------------QIPVVQPSILQQLNPCKLFLQQQCSPVAMPQRLARSQMLQQSSCHVMQQQCCQQLPQIPQQSRYEAIRAIIYSIILQEQ 210
A3-2.pro           SQQQQP--------PFSQQQQPPFSQQ-------------QQIPVIHPSVLQQLNPCKVFLQQQCIPVAMQRCLARSQMLQQSICHVMQQQCCQQLRQIPEQSRHESIRAIVYSIILQQQ 257
A3-4.pro           SQQQQP--------PFSQQQQTPFSQQ-------------QQIPVIHPSVLQQLNPCKVFLQQQCIPVAMQRCLARSQMLQQSICHVMQQQCCQQLRQIPEQSRHESIRAIVYSIILQQQ 290
                   ------------------------------------------------------------------------------------------------------------------------>|
A3-1.pro           Q---------QGFVQAQQQQPQQSGQGVSQSQQQSQQQ--LGQCSFQQPQ--QQLGQQPQQQQ--VQQGTFLQPHQIAHLEVMTSIALRTLPTMCSVNVPLYSSTTSVPFGVGTGVGAY  304
D3-7.pro           Q---------QGFVQPQQQQPQQSGQGVSQSQQQSQQQ--LGQCSFQQPQ--QQLGQQPQQQQQQVLQGTFLQPHQIAHLEAVTSIALRTLPTMCSVNVPLYSATTSVPFGVGTGVGAY  299
D3-4.pro           Q---------QGFVQPQQQQPQQSVQGVYQPQQQSQQQ--LGQCSFQQPQ--QQLGQQPQQQQ--VQKGTFLQPHQIARLEVMTSIALRTLPTMCSVNVPLYSSITSAPLGVGSRVGAY  298
D3-6.pro           Q---------QGFVQPQQQQPQQSGQGVSQHQQQSQQQQQLGQCSFQQPQQLQQLGQQPQQQQ--IPQGIFLQPHQISQLEVMTSIALRTLPTMCGVNVPLYSSTTIMPFSIGTGVGGY  350
B3-1.pro           QH-------GQGLNQPQQQQPQQSVQGVSQPQQQ-QKQ--LGQCSFQQPQQ-QQLGQWPQQQQ--VPQGTLLQPHQIAQLEVMTSIALRTLPTMCSVNVPVYGTTTIVPFGVGTRVGAY  350
D3-1.pro           QH-------GQGFNQPQQQQPQQSVQGVSQPQQQ-QKQ--LGQCSFQRPQQ-QQLGQWPQQQQ--VPQGTLLQPHQIAQLELMTSIALRTLPMMCSVNVPVYGTTTSVPFGVGTQVGAY  365
B3-2.pro           QQ-------VQGSIQTQQQQPQQLGQCVSQPQQQSQQQ----------------LGQQPQQQQ--LAHGTFLQPHQIAQLEVMTSIALRTLPTMCNVNVPLYRTTTRVPFGVGTGVGGY  392
D3-3.pro           QQ-------VQGSIQSQQQQPQQLGQCVSQPQQQSQQQ----------------LGQQPQQQQ--LAQGTFLQPHQIAQLEVMTSIALRTLPTMCRVNVPLYRTTTSVPFGVGAGVGAY  354
D3-2.pro           QQ-------VQGSIQSQQQQPQQLGQCVSQPQQQSQQQ----------------LGQQPQQQQ--LAQGTFLQPHQIAQLEVMTSIALRILPTMCSVNVPLYRTTTSVPFDVGTGVGAY  304
A3-2.pro           QQQQQQQQQRQSIIQYQQQQPQQLGQCVSQPQQQLQQQ----------------LGQQPQQQQ--LTHGAFLQPHQIAQLEVMNSIALRNLPRMCSVNVPLYETTTSVPLGVGIGVGVY  358
A3-4.pro           QQQQQQQQ-GQSIIQYQQQQPQQLGQCVSQPQQQLQQQ----------------LGQQPQQQQ--LAHGTFLQPHQIAQLEVMTSIALHNLPMMCSVNVPLYETTTSVPLGIGIGIGVGVY  390
```

Figure S2. Multiple alignment of the amino acid sequences deduced from the 11 active LMW-GS genes in the bread wheat variety Xiaoyan 54. Among the 11 deduced proteins, two (A3-2, A3-4), two (B3-2, D3-3) and seven (A3-1, B3-1, D3-1, D3-2, D3-4, D3-6, D3-7) were highly similar to typical i-, s-, and m-type subunits, respectively. The nine s- and m-type subunits all possessed a signal peptide (Sig, removed upon maturation), a N-terminal (N-ter) domain, a repetitive (Rep) domain, and a C-terminal (C-ter) domain in their primary structure. The two i-type subunits generally resembled the s- and m-type proteins in the primary structure except that they did not have an intact N-terminal domain. Eight cysteine residues (boxed) were found in each of the 11 deduced subunits.
