# Supplementary material for: New Insights into the Organization, Recombination, Expression and Functional Mechanism of Low Molecular Weight Glutenin Subunit Genes in Bread Wheat
Source: PLoS One. 2010 Oct 21;5(10):e13548. doi: 10.1371/journal.pone.0013548 (PMC2958824; doi:10.1371/journal.pone.0013548)
Supplement: Figure S3 — Identification of protein spots resolved by 2-DE of glutenin samples from As91 and Y207. (0.04 MB PDF) [file pone.0013548.s004.pdf]

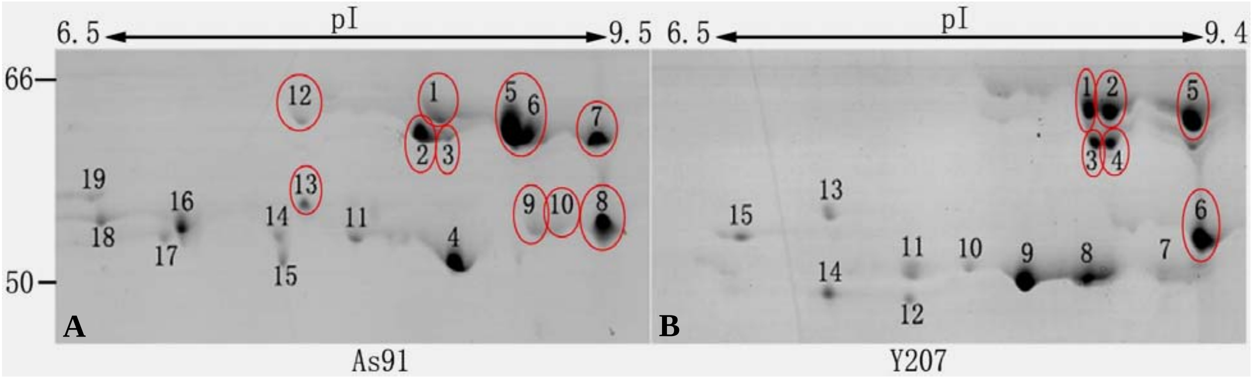

Figure S3. Identification of the protein spots resolved by two dimensional electrophoresis (2-DE) of the glutenin samples from two *Ae. tauschii* accessions (As91, Y207) by mass spectrometry (MS) analysis. Glutenin fractions prepared from the mature grains were used for 2-DE and MS experiments. The high molecular weight glutenin subunit protein spots are not shown. Nineteen and 15 protein spots were identified for As91 (*A*) and Y207 (*B*), respectively, with LMW-GS protein spots circled in red. For both accessions, the glutenin fraction was found to contain some gliadin protein species and the proteins with other or unknown functions (represented by the numbered but uncircled spots, Supplemental Tables 8 and 9). The pI ranges in the 2-DE gels are displayed on the top. The data shown are representative of three independent sets of 2-DE separation and MS analysis.
