# Supplementary material for: New Insights into the Organization, Recombination, Expression and Functional Mechanism of Low Molecular Weight Glutenin Subunit Genes in Bread Wheat
Source: PLoS One. 2010 Oct 21;5(10):e13548. doi: 10.1371/journal.pone.0013548 (PMC2958824; doi:10.1371/journal.pone.0013548)
Supplement: Figure S4 — Significant and positive correlation between number of active LMW-GS genes and mean ZSV. (0.02 MB PDF) [file pone.0013548.s005.pdf]

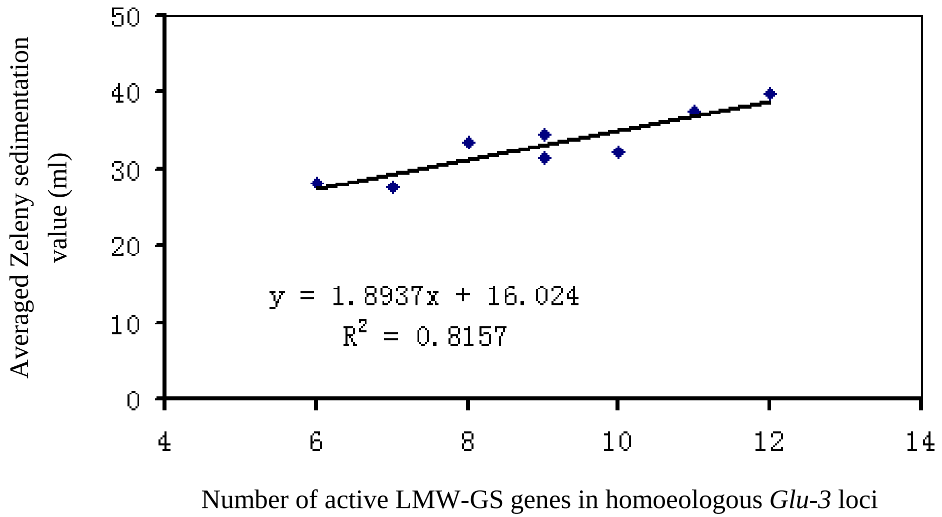

Figure S4. Significant and positive correlation (r = 0.903) between the number of active LMW-GS genes contained in the homoeologous *Glu-3* loci and the averaged Zeleny sedimentation value among the eight main recombinant genotypes identified in the RIL population of Xiaoyan 54 × Jing 411.
