# Supplementary material for: New Insights into the Organization, Recombination, Expression and Functional Mechanism of Low Molecular Weight Glutenin Subunit Genes in Bread Wheat
Source: PLoS One. 2010 Oct 21;5(10):e13548. doi: 10.1371/journal.pone.0013548 (PMC2958824; doi:10.1371/journal.pone.0013548)
Supplement: Table S1 — Oligonucleotide primers used for LMW-GS gene cloning and expression profiling experiments. (0.02 MB PDF) [file pone.0013548.s006.pdf]

Table S1. Oligonucleotide primers used for LMW-GS gene cloning and expression profiling experiments in this work[a]

Primer pair	Name	Sequence (5′-3′)	Use
1	LMW-If<br>LMW-Ir	GC(A/G)GGATCCATGAG(G/T)<br>T(T/C)AGAATCCTCAAACTCCGAT	Degenerate primers for i-type LMW-GS genes, used for BAC library screening
2	LMW-Sf<br>LMW-Sr	GG (A/C) ATTGGATCCATGGAGA<br>TCAGGATCCTCAAACTC(C/G)G(G/C)	Degenerate primers for s-type LMW-GS genes, used for BAC library screening
3	LMW-Mf<br>LMW-Mr	GCCATTGGATCCATGGAGACTAG<br>TCAGGATCCTCAAACTC(C/G)	Degenerate primers for m-type LMW-GS genes, used for BAC library screening
4	LMW-Z1f<br>LMW-Z1r	ATGAAGACCTTCCTC(G/A)TCTTTG<br>CAGTAG(G/A)CACCAACTC(C/G)G	Degenerate primers for LMW-GS genes, used for BAC library screening
5	LMW-Z2f<br>LMW-Z2r	AC(A/C)ACCATTTTC(A/G)CAACAA<br>(C/T)AGGA(C/T)GAT(G/C)GAGTAG	Degenerate primers for LMW-GS genes, used for BAC library screening
6	G1056f<br>G1056r	CCTGGTTTGGAGAGACCATGGC<br>TGTTCTTGTAGGATGATGGGGTAGGT	Specific for *A3-1*, used for BAC library screening
7	G1220f<br>G1220r	CTCATCGCCGTTGTGGCGACAAG<br>TGCCAACGCCGAATGGCACACTG	Specific for *D3-7*, used for BAC library screening
8	A3-1f<br>A3-1r	ATTGCGCAGATGGATACTAGCTG<br>GCTGCAAAAAGGTACCCTGTT	Specific for *A3-1*, used in semiquantitative RT-PCR assay
9	A3-2f<br>A3-2r	GCCGTTGCGCAAATTTCAC<br>CTGCAAAAAGGCACCATGTGT	Specific for *A3-2*, used in semiquantitative RT-PCR assay
10	A3-4f<br>A3-4r	GCCGTTGCGCAAATTTCAC<br>CTGCAAAAAGGTACCATGTGC	Specific for *A3-4*, used in semiquantitative RT-PCR assay
11	B3-1f<br>B3-1r	CCCTAGCTTGGAGAAACCATT<br>GTTGGTTCAAACCCTGACCA	Specific for *B3-1*, used in semiquantitative RT-PCR assay
12	B3-2/D3-3f<br>B3-2/D3-3r	(G/A)CAACAAACATTATCGCACCA<br>TTGGATGGAACCCTGAACCTG	Specific for *B3-2* and *D3-3*, used in semiquantitative RT-PCR assay [b]
13	D3-1f<br>D3-1r	CCATTCTCGCAGCAAGAACT<br>GTTGGTTGAAACCCTGACCA	Specific for *D3-1*, used in semiquantitative RT-PCR assay
14	D3-2f<br>D3-2r	GCGCAGATGGAGACTAGATGC<br>CAAAAAGGTACCCTGTGCCAA	Specific for *D3-2*, used in semiquantitative RT-PCR assay
15	D3-4f<br>D3-4r	GCAACAACCATTACAACAAAAAGAGAC<br>CAGTAGGCACCAACTCGGCT	Specific for *D3-4*, used in semiquantitative RT-PCR assay
16	D3-6f<br>D3-6r	CACAAATGGAGACTAGCCGCG<br>TGCCAATGCTGAATGGCATAA	Specific for *D3-6*, used in semiquantitative RT-PCR assay
17	D3-7f<br>D3-7r	ATTGCACAGATGGAGACTAGCTG<br>GCTGCAAAAAGGTACCCTGTA	Specific for *D3-7*, used in semiquantitative RT-PCR assay
18	Tubulinf<br>Tubulinr	ACCGCCAGCTCTTCCACCCT<br>TCACTGGGGCATAGGAGGAA	Specific for wheat *tubulin* gene transcripts, used in semiquantitative RT-PCR assay

[a] The names labeled by “f” are forward primers, whereas those by “r” are reverse primers. Primer pairs 1 to 17 were used for cloning the LMW-GS genes in Jing 411. Primer pairs 2 to 7 and 13 to 17 were deployed for cloning the LMW-GS genes in two *Ae. tauschii* accessions (As91 and Y207).

[b] The amplified fragments of *B3-2* and *D3-3* were 800 and 684 bp, respectively, which permitted separate evaluation of the transcript levels of the two genes in semiquantitative RT-PCR assay.
