# Supplementary material for: New Insights into the Organization, Recombination, Expression and Functional Mechanism of Low Molecular Weight Glutenin Subunit Genes in Bread Wheat
Source: PLoS One. 2010 Oct 21;5(10):e13548. doi: 10.1371/journal.pone.0013548 (PMC2958824; doi:10.1371/journal.pone.0013548)
Supplement: Table S2 — Oligonucleotide primers used in BAC clone chromosomal localization and LMW-GS gene genetic mapping experiments. (0.02 MB PDF) [file pone.0013548.s007.pdf]

Table S2. Oligonucleotide primers used in BAC clone chromosomal localization and LMW-GS gene genetic mapping experiments in this work[a]

Name	Sequence (5′-3′)	Use
229f	ACTAATCCCATTTGCGTTGC	Assigning five BAC clones (B229-8-7,
229r	TCGCAGGCACACCATAAGTA	B498-5-8, B1354-3-6, B1354-4-8,
		B1777-3-8) to 1BS
57f	GGCTCTCCATTGAAGTGTT	Assigning the BAC clone B57-6-5 to 1BS
57r	GGAGCGACGTTATAAGTATCAG	
357f	TTATGGTACCACCAACAG	Assigning three BAC clones (D78-6-8,
357r	AACCCTGACCATGTTGTTGTTC	D357-11-6, D570-9-3) to 1DS
1862f	GAAAAGAGGTGGTTCCTG	Assigning the BAC clone D1862-8-4 to
1862r	GACTAAACAACGGTGACCCAAT	1DS
1126f	CAAGATCATCACAGGCACAAT	Assigning the BAC clone D1126-1-3 to
1126r	TTATCAGTAGCCACCAACTCCAGT	1DS
LMW-If	GC(A/G)GGATCCATGAG(G/T) GCCGTTGCGCAAATT	Assigning five BAC clones (A708-12-2,
LMW-Ir	T(T/C)AGAATCCTCAAACTCCGATGCCAA(T/C)GCCTAA	A1154-1-1, A1154-1-2, A1380-8-2) to 1AS
A3-1f	CCTGGTTTGGAGAGACCATGGC	Assigning the BAC clone A1056-11-5 to
A3-1r	TGTTCTTGTAGGATGATGGGGTAGGT	1AS, and genetic mapping of *A3-1*
A3-2/3/4f	GCCGTTGCGCAAATTTCAC	Genetic mapping of the *A3-2*, *A3-3* and
A3-2/3/4r	CATTTGTGACCTAGCAAGACATC	*A3-4* cluster
B3-1f	GAGACAATCCATTTGGTATAGAA	Genetic mapping of *B3-1*
B3-1r	GTCAGTCAGCAGGGTGTATT	
B3-2/3f	RCAACAAACATTATCGCACCA	Genetic mapping of the *B3-2* and *B3-3*
B3-2/3r	TTGGATGGAACCCTGAACCTG	cluster
D3-1f	AACCACCATTATCGCAACAA	Genetic mapping of *D3-1*
D3-1r	GTAGACACCTTGAACCGACT	
D3-2/3f	CTGCTGGAGGAATAGCTTGC	Genetic mapping of the *D3-2* and *D3-3*
D3-2/3r	ACCACAGCAACCACCATTTT	cluster
D3-4f	ACATCTGCGACCTAGCAAGATG	Assigning the BAC clone D510-5-7 to
D3-4r	CAAAAAGAGACATTTCCACAAC	1DS, and genetic mapping of *D3-4*
D3-5f	CTGGTCTGATTTTCTCCACTA	Assigning the BAC clone D479-7-6 to
D3-5r	ACACCGACAAGATATGAACC	1DS, and genetic mapping of *D3-5*
D3-6f	ACAAATGGAGACTAGCCGCGT	Genetic mapping of *D3-6*
D3-6r	TGCCAATGCTGAATGGCATAA	
D3-7f	CTCATCGCCGTTGTGGCGACAAG	Assigning the BAC clone D1220-5-2 to
D3-7r	TGCCAACGCCGAATGGCACACTG	1DS, and genetic mapping of *D3-7*

[a] The names labeled by “f” are forward primers, whereas those by “r” are reverse primers.
