# Supplementary material for: New Insights into the Organization, Recombination, Expression and Functional Mechanism of Low Molecular Weight Glutenin Subunit Genes in Bread Wheat
Source: PLoS One. 2010 Oct 21;5(10):e13548. doi: 10.1371/journal.pone.0013548 (PMC2958824; doi:10.1371/journal.pone.0013548)
Supplement: Table S3 — Nucleotide sequence identities of the 14 LMW-GS genes of Xiaoyan 54 to previously reported Glu-A3, B3 or D3 LMW-GS alleles/genes [44]-[46], [47]. (0.03 MB PDF) [file pone.0013548.s008.pdf]

Table S3. Nucleotide sequence identities of the 14 LMW-GS genes of Xiaoyan 54 to previously reported *Glu-A3*, *B3* or *D3* LMW-GS alleles/genes [44-47] [a]

Gene (subunit type)	*Glu-A3a* (i)	*Glu-A3b* (i)	*Glu-A3c* (i)	*Glu-A3d* (i)	*Glu-A3e* (i)	*Glu-A3f* (i)	*Glu-A3g* (i)	*GluB3-1* (s)	*GluB3-2* (s)	*GluB3-3* (s)	*GluB3-4* (m)	*GluD3-1* (m)	*GluD3-2* (m)	*GluD3-3* (s)	*GluD3-4* (m)	*GluD3-5* (m)	*GluD3-6* (m)
A3-1 (m)	57.2	55.7	56.8	60.1	55.9	57.2	56.1	—	—	—	—	—	—	—	—	—	—
A3-2 (i)	89.7	90.4	89.8	99.2	88.7	92.7	88.9	—	—	—	—	—	—	—	—	—	—
A3-3 (i) [b]	89.8	90.2	89.9	99.4	88.6	92.4	88.7	—	—	—	—	—	—	—	—	—	—
A3-4 (i)	92.1	94.3	92.1	88.9	92.5	85.4	92.6	—	—	—	—	—	—	—	—	—	—
B3-1 (m)	—	—	—	—	—	—	—	91.5	88.0	90.3	94.3	—	—	—	—	—	—
B3-2 (s)	—	—	—	—	—	—	—	90.7	90.9	99.5	92.8	—	—	—	—	—	—
B3-3 (s) [c]	—	—	—	—	—	—	—	92.3	90.8	99.6	93.4	—	—	—	—	—	—
D3-1 (m)	—	—	—	—	—	—	—	—	—	—	—	89.3	81.3	91.91	76.3	99.9	76.4
D3-2 (m)	—	—	—	—	—	—	—	—	—	—	—	91.2	99.4	90.0	91.1	72.7	90.0
D3-3 (s)	—	—	—	—	—	—	—	—	—	—	—	88.5	82.5	99.8	75.6	83.8	75.4
D3-4 (m)	—	—	—	—	—	—	—	—	—	—	—	89.1	92.1	85.2	95.5	66.0	99.8
D3-5 (m) [d]	—	—	—	—	—	—	—	—	—	—	—	91.5	92.4	84.9	94.9	64.4	97.1
D3-6 (m)	—	—	—	—	—	—	—	—	—	—	—	99.7	84.2	89.3	83.1	79.2	79.6
D3-7 (m)	—	—	—	—	—	—	—	—	—	—	—	91.5	91.9	84.3	99.1	64.2	94.3

[a] [44]: Zhao XL, Xia XC, He ZH, Gale KT, Lei ZS, Appels R, Ma WJ. 2006. Characterization of three low-molecular-weight *Glu-D3* subunit genes in common wheat. *Theor Appl Genet* 113: 1247-1259. [45]: Zhao XL, Xia XC, He ZH, Lei ZS, Appels R, Yang Y, Sun QX, Ma WJ. 2007. Novel DNA variations to characterize low molecular weight glutenin *Glu-D3* genes and develop STS markers in common wheat. *Theor Appl Genet* 114: 451-460. [46]: Wang LH, Zhao XL, He ZH, Ma W, Appels R, Peña RJ, Xia XC. 2009. Characterization of low-molecular-weight glutenin subunit *Glu-B3* genes and development of STS markers in common wheat (*Triticum aestivum* L.). *Theor Appl Genet* 118: 525-539. [47]: Zhang W, Gianibelli MC, Rampling L, Gale KR. 2004. Characterization and marker development for low molecular weight glutenin genes from *Glu-A3* alleles of bread wheat. *Theor Appl Genet* 108: 1409-1419.

[b] This is an i-type subunit pseudogene. The fragment used for nucleotide sequence comparison was 1074 bp.

[c] This is a s-type subunit pseudogene. The fragment used for nucleotide sequence comparison was 390 bp.

[d] This is a m-type subunit pseudogene. The fragment used for nucleotide sequence comparison was 903 bp.
