# Supplementary material for: New Insights into the Organization, Recombination, Expression and Functional Mechanism of Low Molecular Weight Glutenin Subunit Genes in Bread Wheat
Source: PLoS One. 2010 Oct 21;5(10):e13548. doi: 10.1371/journal.pone.0013548 (PMC2958824; doi:10.1371/journal.pone.0013548)
Supplement: Table S4 — Characterization of protein spots resolved by 2-DE of the glutenin fraction of Xiaoyan 54 with MALDI-TOF-MS and LC-MS/MS analyses. (0.04 MB PDF) [file pone.0013548.s009.pdf]

Table S4. Characterization of the protein spots resolved by 2-DE separation of the glutenin fraction of Xiaoyan 54 with MALDI-TOF-MS and LC-MS/MS analysis[a]

	MALDI-TOF-MS					LC-MS/MS			
Spot	Protein	Coverage (%) [b]	Theoretical mass (kD)	Theoretical pI	Peptide mass tolerance (Da)	Mass spectrum [d]	MH^+ [e]	Charge	XC [f]
1	**LMW-GS**	23.7	31.7	10.3	0.25				
2	**LMW-GS**	35	31.7	10.3	0.25				
3	**LMW-GS**	39.8	31.7	10.3	0.48				
4	Unknown [c]	--	--	--	--				
5	**LMW-GS**	33.3	29.8	10.3	0.25				
6	**LMW-GS**	30.3	39.2	10.1	0.5				
7	gamma-gliadin	--	--	--	--	L.EAIRSL.V	688.8	1	1.22
						L.AQIPQQL.Q	797.92	1	1.32
						F.ASIVAGIGGQ.-	872.99	1	1.74
						L.VSSLW.S	591.68	1	1.51
8	**LMW-GS**	21.2	28.6	9.9	0.25				
9	**LMW-GS**	38.3	34.3	9.8	0.22				
10	**LMW-GS**	25	39.9	9.8	0.15				
11	**LMW-GS**	39.5	39.9	9.8	0.2				
12	**LMW-GS**	24.8	40.3	9.9	0.2				
13	**LMW-GS**	32.3	39.9	9.8	0.18				
14	**LMW-GS**	25.2	39.3	9.9	0.25				
15	**LMW-GS**	31.2	41.4	10.7	0.31				
16	gamma-gliadin	--	--	--	--	L.AQQQIPVVQPSIL.Q	1421.67	1	1.17
17	Unknown	--	--	--	--				
18	alpha-gliadin	42.6	31	7.9	0.3				

19	alpha-gliadin	40.8	34	9.8	0.3				
20	alpha-gliadin	47.2	31	6.7	0.2				
21	alpha-gliadin	--	--	--	--	L.GIIQPQQPAQL.E	1193.38	2	2.39
						Y.ANIDAGIGGQ.-	915.97	1	1.88
						L.AQIPQQL.Q	797.92	1	1.42
22	alpha-gliadin	32.8	32.3	9	0.15				
23	alpha-gliadin	51.8	32.5	7.8	0.2				
24	alpha-gliadin	41.9	31	6.7	0.25				
25	Unknown	--	--	--	--				
26	ribulose-1,5-bisphosphate carboxylase and oxygenase, large subunit (EMBL accession number AAQ75640)	32	50.1	6.1	0.2				
27	alpha-gliadin	--	--	--	--	Y.IPPHC#STTIAPF.G	1341.53	2	1.89
						Y.QLLQQL.C	742.89	1	1.87
						L.QQQLIPC#RDVVL.Q	1469.71	2	1.69
						F.GIFGTN.-	608.6	1	1.22
28	alpha-gliadin	--	--	--	--	L.HQQRQQPSSQVSF.Q	1557.65	2	4.04
						Y.QLLQQL.C	742.89	1	1.67
						Y.IPPHC#STTIAPF.G	1341.53	2	1.55
						F.GIFGTN.-	608.67	1	1.43
29	alpha-gliadin	43.8	34.1	8.9	0.2				
30	Unknown	--	--	--	--				
31	Unknown	--	--	--	--				
32	alpha-gliadin	49.1	32.3	9	0.3				

33	**LMW-GS**	28.1	32.3	9.2	0.2
34	Unknown	--	--	--	--
35	Unknown	--	--	--	--
36	alpha-gliadin	39.4	32.7	9	0.15
37	alpha-gliadin	55.9	31.8	6.7	0.2
38	**LMW-GS**	26.8	33.1	9.7	0.15
39	alpha-gliadin	58.7	31.6	9	0.3
40	alpha-gliadin	34.6	31.6	9	0.15
41	**LMW-GS**	44.6	34.6	9.7	0.15
42	**LMW-GS**	18.1	32.3	9.2	0.15
43	alpha-gliadin	41.1	34.6	9.5	0.2
44	alpha-gliadin	26.2	34.1	8.9	0.2
45	Unknown	--	--	--	--
46	Unknown	--	--	--	--
47	**LMW-GS**	47	33.1	10	0.25
48	**LMW-GS**	46.3	34.3	10.4	0.3
49	Unknown	--	--	--	--
50	Unknown	--	--	--	--
51	NAM protein (GenBank accession number ABE93368)	38	40.7	8.9	0.3
52	Unknown	--	--	--	--
53	gamma-gliadin	21.1	33	10.1	0.15
54	gamma-gliadin	36.5	14.7	12.1	0.35
55	Unknown	--	--	--	--
56	Unknown	--	--	--	--

57	Tropinone reductase I (EMBL accession number CAC34420)	30.7	28.9	7.6	0.2			
58	Unknown	--	--	--	--			
59	Unknown	--	--	--	--			
60	Unknown	--	--	--	--			

[a] In-gel digestion of protein spots for MALDI-TOF and LC-MS/MS analyses was conducted with chymotrypsin. The parameters for database searching with Biotools 2.1 were: one missed cleavage and 0.15 to 0.5 Da peptide mass tolerance. The identification of several gliadin spots was only possible with LC-MS/MS.

[b] Percentage of predicted protein sequence covered by matched peptides.

[c] High quality mass spectrometry data was obtained, but no hit was found after searching the NCBI database.

[d] The “#” symbol in the peptide denotes the cysteine residue with carbamidomethyl modification.

[e] MH^+ , the m/z of protonated molecular ion of the corresponding peptide.

[f] Cross-correlation value computed from cross-correlating the experimental MS/MS spectrum vs candidate peptides in the database (significant score: ≥1 for single-charged ions, ≥1.5 for doubly-charged ions).
