# Supplementary material for: New Insights into the Organization, Recombination, Expression and Functional Mechanism of Low Molecular Weight Glutenin Subunit Genes in Bread Wheat
Source: PLoS One. 2010 Oct 21;5(10):e13548. doi: 10.1371/journal.pone.0013548 (PMC2958824; doi:10.1371/journal.pone.0013548)
Supplement: Table S5 — Matching LMW-GS protein spots resolved by 2-DE to the proteins predicted from the cloned active LMW-GS genes using the mass spectragraphs generated by LC-MS/MS analysis in Xiaoyan 54. (0.05 MB PDF) [file pone.0013548.s010.pdf]

Table S5. Matching LMW-GS protein spots resolved by 2-DE to the proteins predicted from the cloned active LMW-GS genes using the mass spectra generated by LC-MS/MS analysis in Xiaoyan 54

Spot	Mass spectrum [a]	MH^{+} [b]	Charge	XC [c]	Matching gene	Predicted LMW-GS protein sequence [d]
1	F.SQQQQIPVIHPSVL.Q	1574.81	2	2.24	*A3-4*	MKTFLVFALLALAAASAVAQISQQQQQPPFSQQQQPPF**SQQQ**
2	Y.QQQQPQQL.G	998.08	1	1.30		**QPPFSQQQQSPF**SQQQEQQQQPPFLQQQQPPFSQQPPISQQQ
	L.GQC#VSQPQQQL.Q	1273.37	2	2.17		QPPFSQEQQPPF**SQQQQPPFSQQQQPPYSQQQQPPFSQQQQ**
	L.QQQLGQQPQQQQL.A	1552.68	2	3.90		**PPF**SQQQQPPFSQQQQQQQQQQQPFTQQQPPFSQQPPISQQ
	F.SQQQQPPFSQQQQPPY.S	1917.03	2	2.61		QQQQQQQQQQPFTQQQPPFSQQPPISQQQQPPFSQQQQTPF**S**
	L.GQC#VSQPQQQLQQQL.G	1770.92	2	2.83		**QQQQIPVIHPSVL**QQLNPCKVFLQQQCIPVAMQRCLARSQM
	F.SQQQQPPFSQQQQSPF.S	1890.99	2	2.81		LQQSICHVMQQQCCQQLRQIPEQSRHESIRAIVYSIILQQQQQ
	F.SQQQQPPFSQQQQPPF.S	1901.03	2	2.50		QQQQQQGQSIIQY**QQQQPQQLGQCVSQPQQQLQQQLGQQ**
	F.SQQQQIPVIHPSVL.Q	1574.81	2	3.39		**PQQQQL**AHGTFLQPHQIAQLEVMTSIALHNLPMMCSVNVPL
3	L.QQQLGQQPQQQQL.A	1552.68	2	2.30		YETTTSVPLGVGIGVGVY
	F.SQQQQPPFSQQQQPPF.S	1901.03	2	1.79		
	F.SQQQQIPVIHPSVL.Q	1574.81	2	3.57		
5	L.GQC#VSQPQQQSQQQL.G	1744.84	2	2.69	*B3-2*	MKTFLIFALLAVAATSAIAQMENSHIPGLERPSQQQPLPPQQTL
	L.QQLNPC#KVF.L	1134.30	2	2.14		SHHHQQQPIQQQPHQFPQQQPCSQQQQQPPLSQQQQPPFSQQ
	F.GVGTGVGGY.-	766.82	1	1.67		QQPPFSQQQQPVLPQQPSFSQQQLPPFSQQQQPPFSQQQQPVL
	Y.RTTTRVPF.G	978.13	2	1.66		PQQPSFSQQQLPPFSQQLPPFSQQQQPVLPQQPPFSQQQLPPFS
	Y.EAIRAIVY.S	935.10	1	1.62		QQLPPFSQQQQPVLPQQPPFSQQQQQPILPQQPPFSQQQQPVL
	L.ARSQML.Q	705.85	1	1.51		L**QQQIPFVHPSILQQLNPCKVF**LQQQCSPVAMPQSL**ARSQM**
	F.VHPSIL.Q	665.80	1	1.32		**L**QQSSCHVMQQQCCQQLPQIPQQSRY**EAIRAIVY**SIILQEQQQ
	L.GQQPQQQQL.A	1055.13	1	1.22		VQGSIQTQQQQPQQL**GQCVSQPQQQSQQQLGQQPQQQQL**
	L.QQQIPF.V	760.86	1	1.21		**AHGTF**LQPHQIAQLEVMTSIALRTLPTMCNVNVPLY**RTTTRV**

6	Y.EAIRAIVY.S	935.10	1	1.92		**PFGVGTGVGGY**
	L.QQLNPC#KVF.L	1134.30	2	1.71		
	F.GVGTGVGGY.-	766.82	1	1.54		
	L.ARSQML.Q	705.85	1	1.23		
	F.VHPSIL.Q	665.80	1	1.21		
	L.AHGTF.L	532.57	1	1.11		
	L.QQQIPF.V	760.86	1	1.07		
8	F.SIGTGVGGY.-	810.88	1	1.40	*D3-6*	MKTFLIFALLAIAATSAIAQMETSRVPGLEKPWQQQPLPPQQQ
	F.SQQQQPVLPQQQPVIIL.Q	1945.25	2	3.27		PPCSQQQQPFPQQQQPIIILQQSPF**SQQQQPVLPQQQPVIIL**Q
	Y.SIILQEQQQGF.V	1291.44	2	1.81		QPPFSQQQQPVLPQQPPFSQQQQQQQQQPPFSQQQQPVLPQ
9	F.SIGTGVGGY.-	810.88	1	1.40		QPPFSQQQQPPFSQQQQPSSQQPPFPQQHQQFPQQQIPVVQPS
	Y.SIILQEQQQGF.V	1291.44	2	1.95		VLQQLNPCKVFLQQQCSHVAMSQRLARSQMWQQSSCHVMQ
	L.QQLGQQPQQQQIPQGIF.L	1967.18	2	1.51		QQCCQQLPQIPEQSRSEAIRAIVY**SIILQEQQQGF**VQPQQQQP
	F.SQQQQPVLPQQQPVIIL.Q	1945.25	2	3.36		QQSGQGVSQHQQQSQQQQQLGQCSFQQPQQL**QQLGQQPQ**
						**QQQIPQGIF**LQPHQISQLEVMTSIALRTLPTMCGVNVPLYSST
						TIMPF**SIGTGVGGY**
10	F.NQPQQQQPQQSVQGVSQPQQQQKQL.G	2889.09	3	4.66	*D3-1*	MKTFLIFALLAVAATSAIAQMETSHIPGLEKPSQQQPLPL**QQIL**
	Y.SIVLQEQQHGQGF.N	1471.60	2	2.80		**W**YHQQQPIQQQPQPFPQQPPCSQQQQPPLSQQQQPPFSQQQP
	F.SQHQQPVLPQQQIPY.V	1791.99	2	2.32		PFSQQELPILPQQPPFSQQQQPQFSQQQQPFPQQQQPL**LLQQP**
	L.QQLNPC#KVF.L	1134.30	2	2.12		**PFSQQRPPF**SQQQQQPVLPQQPPFSQQQQQQPILPQQPPF**SQH**
	L.LLQQPPF.S	843.01	1	1.64		**QQPVLPQQQIPYVQPSILQQLNPCKVF**LQQQCSPVAMPQSL
	Y.DAIRAIIY.S	935.10	1	1.51		ARSQMLWQSSCHVMQQQCCQQLPRIPEQSRY**DAIRAIIYSIV**
	F.GVGTQVGAY.-	851.93	1	1.15		**LQEQQHGQGFNQPQQQQPQQSVQGVSQPQQQQKQL**GQC
11	F.NQPQQQQPQQSVQGVSQPQQQQKQL.G	2889.09	3	3.38		SFQRPQQQQLGQWPQQQQVPQGTLLQPHQIAQLELMTSIALR
	F.SQHQQPVLPQQQIPY.V	1791.99	2	2.75		TLPMMCSVNVPVYGTTTSVPF**GVGTQVGAY**
	Y.SIVLQEQQHGQGF.N	1471.60	2	2.43		

	L.QQLNPC#KVF.L	1134.30	2	2.38		
	L.LLQQPPF.S	843.01	1	2.27		
	F.QRPQQQQL.G	1026.13	2	1.76		
	Y.DAIRAIIY.S	935.10	1	1.60		
	F.SQQRPPF.S	859.95	1	1.57		
	Y.VQPSIL.Q	656.79	1	1.17		
	L.QQILW.Y	687.81	1	1.13		
	L.LQQPPF.S	729.85	1	1.13		
	F.GVGTQVGAY.-	851.93	1	1.15		
12	L.NQPQQQQPQQSVQGVSQPQQQQKQL.G	2889.09	3	4.07	*B3-1*	MKTFLIFALLAVAATSAIAQMETSHIPSLEKPL**QQQPLPLQQIL**
	F.GVGTRVGAY.-	879.98	2	2.83		**W**YQQQQPIQQQPQPFPQQPPCSQQQQPPLSQQQQPPFSQQQP
	Y.SIVLQEQQHGQGL.N	1437.58	2	2.61		PFSQQQQPILPQQPPFSQQQQQFPQQQQPLLPQQPPFSQQQPPF
	L.QQLNPC#KVF.L	1134.30	2	2.42		SQQQQQPPFSQQQQQPILLQQPPFSQHQQPVLPQQQIPSVQPSI
	L.RTLPTM*C#SVNVPVY.G	1653.92	2	1.60		L**QQLNPCKVF**LQQQCSPVAMPQSLARSQMLWQSSCHVMQQ
	Y.DAIRAIIY.S	935.10	1	1.49		QCCRQLPQIPEQSRY**DAIRAIIYSIVLQEQQHGQGLNQPQQQ**
	L.QQILW.Y	687.81	1	1.38		**QPQQSVQGVSQPQQQQKQL**GQCSFQQPQQQQLGQWPQQQ
	L.QQQPLPL.Q	823.96	1	1.24		QVPQGTLLQPHQIAQLEVMTSIAL**RTLPTMCSVNVPVYGTT**
	Y.GTTTIVPF.G	835.97	1	1.11		**TIVPFGVGTRVGAY**
13	L.NQPQQQQPQQSVQGVSQPQQQQKQL.G	2889.09	3	4.07		
	Y.SIVLQEQQHGQGL.N	1437.58	2	2.96		
	L.QQLNPC#KVF.L	1134.30	2	2.61		
	Y.DAIRAIIY.S	935.10	1	1.61		
	L.QQQPLPL.Q	823.96	1	1.53		
	F.GVGTRVGAY.-	879.98	1	1.25		
	L.QQILW.Y	687.81	1	1.25		
	Y.GTTTIVPF.G	835.97	1	1.11		

14					*D3-3*	MKTFLIFALLAVAATSAIAQIENSHIPGLEKPSQQQPLPLQQTLS
	L.QQLNPC#KVF.L	1134.30	2	2.66		HHQQQQPVQQQPQPFPQQQPCSQQQQPPLSQQQQPPFSQQQP
	F.LQPHQIAQL.E	1048.22	2	2.46		PFSQQQQPSFSQQQQPPFSQQQPPFSQQQQPVIPQQPSFSQQQL
	Y.EAIRAIIY.S	949.13	2	1.82		PPFSQQQPPFSQQQQPVLPQQPPFSQQQQPILPQQPPFSQQQQ
	F.GVGAGVGAY.-	750.82	1	1.49		QPVLPQQQIPF**VHPSILQQLNPCKVF**LQQQCSPVAMPQSL**AR**
	L.ARSQML.Q	705.85	1	1.47		**SQML**QQSSCHVMQQQCCQQLPQIPQQSRY**EAIRAIIY**SIILQE
	Y.RTTTSVPF.D	909.02	1	1.33		QQQVQGSIQSQQQQPQQLGQCVSQPQQQSQQQLGQQPQQQQ
	F.VHPSIL.Q	665.80	1	1.25		LAQGTF**LQPHQIAQL**EVMTSIALRTLPTMCRVNVPLY**RTTTS**
						**VPFGVGAGVGAY**
15	L.QQLNPC#KVF.L	1134.30	2	2.45	*A3-2*	MKTFLVFALLALAAASAVAQISQQQQPPLF**SQQQQPPFSQQQ**
	L.GVGIGVGVY.-	820.96	1	1.32		**QPPF**SQQQQSPFSQQQQPPFSQQQQPPFSQQPPISQQQQPQFL
	L.SQQQQPPFSQQQQPPF.S	1901.03	2	2.12		QQQQQPPFSQQQQPPFSQQQQPPYSQQQQPPFSQQQQPPFSQ
	F.SQQQQIPVIHPSVL.Q	1574.81	2	2.67		QQQPPFSQQQQQQQPPFTQQQQPPFSQQPPISQQQQPPFSQQQ
						QPPF**SQQQQIPVIHPSVLQQLNPCKVF**LQQQCIPVAMQRCL
						ARSQMLQQSICHVMQQQCCQQLRQIPEQSRHESIRAIVYSIIL
						QQQQQQQQQQQQRQSIIQYQQQQPQQLGQCVSQPQQQLQQ
						QLGQQPQQQQLTHGAFLQPHQIAQLEVMNSIALRNLPRMCSV
						NVPLYETTTSVPL**GVGIGVGVY**
33	L.QQLNPC#KVF.L	1134.30	2	2.77	*A3-1*	MKTFLIFALLAVVATSAIAQMDTSCIPGLERPWQQQPLPPQQT
	L.SQIPEQSRY.D	1108.19	2	1.87		FPQQPPFSQQQQQQQQQPFPQQPSFSQQQPPFSQQQPILPQGPP
	L.RTLPTMC#SVNVPLY.S	1651.95	2	1.57		FSQQTQPVLPQQSPFSQQQQLILPPQQQQQLPQQQISIVQPSIL
	F.QQPQQQL.G	869.95	1	1.39		**QQLNPCKVF**LQQQCSPVVMPQRLARSQMWQQSSCHVMQQ
	F.GVGTGVGAY.-	780.85	1	1.05		QCCQQL**SQIPEQSRY**DAIRAITYPIILQEQQQGFVQAQQQQPQ
42	L.QQLNPC#KVF.L	1134.30	2	2.07		QSGQGVSQSQQQSQQQLGQCSF**QQPQQQL**GQQPQQQQVQQ
	F.GVGTGVGAY.-	780.85	1	1.41		GTFLQPHQIAHLEVMTSIAL**RTLPTMCSVNVPLY**SSTTSVPF**G**

						**VGTGVGAY**
38	L.QQLNPC#KVF.L	1134.30	2	2.57	*D3-7*	MKTFLIFALLAVVATSAIAQMETSCISGLERPWQQQPLPPQQSF
	L.VLPPQQQQQQL.V	1307.48	2	1.83		SQQPPFSQQQQQPLPQQPSFSQQQPPFSQQQPILSQQPPFSQQQ
	L.QQIPEQSRY.E	1149.24	2	1.52		QPVLPQQSPFSQQQQP**VLPPQQQQQQL**VQQQIPIVQPSVL**QQ**
	F.GVGTGVGAY.-	780.85	1	1.38		**LNPCKVF**LQQQCSPVAMPQRLARSQMWQQSSCHVMQQQCC
41	L.QQIPEQSRY.E	1149.24	2	1.52		QQL**QQIPEQSRY**EAIRAIILQEQQQGFVQPQQQQPQQSGQGV
						SQSQQQSQQQLGQCSFQQPQQQLGQQPQQQQQQVQQGTFLQ
						PHQIAHLEAVTSIALRTLPTMCSVNVPLYSATTSVPF**GVGTGV**
						**GAY**
47	L.GQC#VSQPQQQSQQQLGQQPQQQQL.A	2780.94	3	3.35	*D3-2*	MKTFLVFALLAVAATSAIAQMETRCIPGLERPW**QQQPLPPQQ**
	L.GQC#VSQPQQQSQQQL.G	1744.84	2	2.95		**TFPQQPL**FSQQQQLFPQQPSF**SQQQPPFWQQQPPF**SQQQPIL
	F.SQQQPPFWQQQPPF.S	1743.90	2	2.91		PQQPPFSQQQQLVLPQQPPFSQQQQPVLPPQQSPFPQQQQHQ
	W.QQQPLPPQQTFPQQPL.F	1876.11	2	2.47		QL**VQQQIPVVQPSILQQLNPCKLF**LQQQCSPVAMPQRL**ARS**
	L.QQLNPC#KLF.L	1148.33	2	2.05		**QML**QQSSCHVMQQQCCQQLPQIPQQSRY**EAIRAIIY**SIILQEQ
	Y.EAIRAIIY.S	949.13	2	2.02		QQVQGSIQSQQQQPQQL**GQCVSQPQQQSQQQLGQQPQQQ**
	L.GQC#VSQPQQQSQQQLGQQPQQQQL.A	2780.94	2	1.87		**QL**AQGTFLQPHQIAQLEVMTSIALRILPTMCSVNVPLY**RTTTS**
	W.QQQPPF.S	744.82	1	1.50		**VPFDVGTGVGAY**
	Y.EAIRAIIY.S	949.13	1	1.44		
	L.VQQQIPVVQPSIL.Q	1449.72	1	1.29		
	L.ARSQML.Q	705.85	1	1.27		
	Y.RTTTSVPF.D	909.02	1	1.18		
	F.DVGTGVGAY.-	838.89	1	1.06		

48	F.VQPQQQQPQQSVQGVY.Q	1842.99	2	3.22	*D3-4*	MKTFLIFALLAVVATSAIAQMETSCIPGLERPWQQQPLQQKET FPQQPPSSQQQQPFPQQPPFLQQQPSFSQQPL**FSQKQQPVLPQ QPAF**SQQQQTVLPQQPAFPQQQHQQLLQQQIPIVHPSIL**QQLN PCKVF**LQQQCSPVAMPQHLARSQMWQQSSCNVMQQQCCQ QLPRIPEQSRY**EAIRAIIF**SIILQEQQQGF**VQPQQQQPQQSVQ GVY**QPQQQSQQQLGQCSFQQPQQQLGQQPQQQQVQKGTFL QPHQIARLEVMTSIALRTLPTMCSVNVPLYSSITSAPLGVGSRV GAY
	L.FSQKQQPVLPQQPAF.S	1743.99	2	2.85		
	L.QQLNPC#KVF.L	1134.30	2	2.40		
	F.SQKQQPVLPQQPAF.S	1596.81	2	1.94		
	Y.EAIRAIIF.S	933.13	1	1.81		

[a] The “#” and “*” symbols in the peptides denote the cysteine residue with carbamidomethyl and the methionine residue with oxidation modifications, respectively.

[b] MH^+ , the m/z of protonated molecular ion of the corresponding peptide.

[c] Cross-correlation value computed from cross-correlating the experimental MS/MS spectrum vs candidate peptides in the database (significant score: ≥1 for single-charged ions, ≥1.5 for doubly-charged ions, ≥2.5 for triply-charged ions).

[d] The peptides written in blue in the predicted protein represent those identified by LC-MS/MS from the corresponding excised protein spot.
