# Supplementary material for: New Insights into the Organization, Recombination, Expression and Functional Mechanism of Low Molecular Weight Glutenin Subunit Genes in Bread Wheat
Source: PLoS One. 2010 Oct 21;5(10):e13548. doi: 10.1371/journal.pone.0013548 (PMC2958824; doi:10.1371/journal.pone.0013548)
Supplement: Table S6 — Characterization of the protein spots resolved by 2-DE of the glutenin fraction of Jing 411 with MALDI-TOF-MS and LC-MS/MS analyses. (0.03 MB PDF) [file pone.0013548.s011.pdf]

**Table S6.** Characterization of the protein spots resolved by 2-DE separation of the glutenin fraction of Jing 411 with MALDI-TOF-MS and LC-MS/MS analyses[a]

| | MALDI-TOF-MS | | | | | LC-MS/MS | | | |
|---|---|---|---|---|---|---|---|---|---|
| Spot | Protein | Coverage (%) [b] | Theoretical mass (kD) | Theoretical pI | Peptide mass tolerance (Da) | Mass spectrum [d] | $MH^{+}$ [e] | Charge | XC [f] |
| 1 | gamma-gliadin | -- | -- | -- | -- | L.LQQC#KPASL.V | 1045.21 | 2 | 2.08 |
| | | | | | | L.EAIRSL.V | 688.80 | 1 | 1.18 |
| | | | | | | F.ASIVAGIGGQ.- | 872.99 | 1 | 1.96 |
| | | | | | | L.AQIPQQL.Q | 797.92 | 1 | 1.36 |
| | | | | | | L.VSSLW.S | 591.68 | 1 | 1.27 |
| 2 | gamma-gliadin | -- | -- | -- | -- | Y.LQQQM*NPC#KNY.L | 1440.54 | 2 | 2.83 |
| | | | | | | Y.LQQQMNPC#KNY.L | 1424.60 | 2 | 3.06 |
| | | | | | | F.PQPQQPF.S | 841.93 | 1 | 1.15 |
| | | | | | | L.EVIRSL.V | 716.85 | 1 | 1.65 |
| | | | | | | F.VPPEC#STTKAPF.A | 1334.49 | 2 | 2.59 |
| | | | | | | F.ASIVADIGGQ.- | 931.03 | 1 | 2.26 |
| 3 | alpha-gliadin | -- | -- | -- | -- | F.PSQQPY.L | 719.77 | 1 | 1.13 |
| | | | | | | L.QQQQQQQL.Q | 1029.09 | 2 | 1.65 |
| | | | | | | L.QQQRQQPSSQVSF.Q | 1548.64 | 2 | 2.12 |
| | | | | | | L.C#C#QQLL.Q | 821.93 | 1 | 1.06 |
| | | | | | | F.QQPQQQYPSSQVSF.Q | 1652.75 | 2 | 2.80 |
| | | | | | | Y.IPPHC#STTIAPF.G | 1341.53 | 2 | 2.23 |
| 4 | alpha-gliadin | 45.1 | 32.3 | 9 | 0.2 | | | | |
| 5 | **LMW-GS** | 28.6 | 33.9 | 10.2 | 0.2 | | | | |

| | | | | | | | | | |
|---|---|---|---|---|---|---|---|---|---|
| 6 | **LMW-GS** | 22.6 | 29.8 | 10.3 | 0.08 | | | | |
| 7 | alpha-gliadin | 35.1 | 31 | 7.9 | 0.2 | | | | |
| 8 | alpha-gliadin | 26.8 | 31 | 7.9 | 0.3 | | | | |
| 9 | Unknown [c] | -- | -- | -- | -- | | | | |
| 10 | **LMW-GS** | 27.3 | 39.9 | 9.8 | 0.15 | | | | |
| 11 | **LMW-GS** | 27.4 | 39.9 | 9.8 | 0.2 | | | | |
| 12 | **LMW-GS** | 30.3 | 39.4 | 10 | 0.15 | | | | |
| 13 | **LMW-GS** | 30.6 | 39.4 | 10 | 0.18 | | | | |
| 14 | **LMW-GS** | 31.6 | 33.4 | 10.9 | 0.2 | | | | |
| 15 | gamma-gliadin | -- | -- | -- | -- | L.EAIRSL.V | 688.80 | 1 | 1.65 |
| | | | | | | L.NPC#KNIL.L | 859.00 | 1 | 1.43 |
| | | | | | | L.QTLPSM*C#NVY.V | 1229.32 | 1 | 1.02 |
| | | | | | | F.IQPSLQQQL.N | 1055.21 | 1 | 1.50 |
| | | | | | | L.AQIPQQL.Q | 797.92 | 1 | 1.52 |
| | | | | | | F.ASIVAGIGGQ.- | 872.99 | 1 | 2.05 |
| | | | | | | L.NPC#KNILL.Q | 972.16 | 1 | 1.56 |
| | | | | | | L.VSSLW.S | 591.68 | 1 | 1.00 |
| | | | | | | L.SQQQQVGQGSLVQGQGIIQPQQPAQL.E | 2747.02 | 2 | 5.07 |
| 16 | alpha-gliadin | 45.7 | 31.6 | 9 | 0.3 | | | | |
| 17 | alpha-gliadin | 45.4 | 31.6 | 9 | 0.3 | | | | |
| 18 | alpha-gliadin | 37.1 | 30.3 | 6.2 | 0.4 | | | | |
| 19 | gamma-gliadin | 36.5 | 14.7 | 12.1 | 0.3 | | | | |
| 20 | gamma-gliadin | 22.8 | 33 | 10.1 | 0.15 | | | | |

| | | | | | |
|---|---|---|---|---|---|
| 21 | putative avenin-like b precursor (EMBL accession number CAJ32659) | 37 | 33.4 | 9.4 | 0.3 |
| 22 | ATP6-1 (RefSeq accession number YP_398396) | 30.2 | 19.7 | 4.7 | 0.15 |
| 23 | putative avenin-like b precursor (EMBL accession number CAJ32655) | 36.5 | 33.8 | 9.8 | 0.2 |
| 24 | **LMW-GS** | 37.9 | 33.1 | 10 | 0.3 |
| 25 | **LMW-GS** | 26.6 | 34.3 | 10.6 | 0.15 |
| 26 | **LMW-GS** | 25.9 | 33.9 | 10.4 | 0.15 |
| 27 | **LMW-GS** | 25.7 | 34.4 | 11.3 | 0.15 |

[a] In-gel digestion of protein spots for MALDI-TOF and LC-MS/MS analyses was conducted with chymotrypsin. The parameters for database searching with Biotools 2.1 were: one missed cleavage and 0.15 to 0.5 Da peptide mass tolerance. The identification of several gliadin spots was only possible with LC-MS/MS.

[b] Percentage of predicted protein sequence covered by matched peptides.

[c] High quality mass spectrometry data was obtained, but no hit was found after searching the NCBI database.

[d] The “#” and “*” symbols in the peptides denote the cysteine residue with carbamidomethyl and the methionine residue with oxidation modifications, respectively.

[e] $MH^+$ , the m/z of protonated molecular ion of the corresponding peptide.

[f] Cross-correlation value computed from cross-correlating the experimental MS/MS spectrum vs candidate peptides in the database (significant score: ≥1 for single-charged ions, ≥1.5 for doubly-charged ions).
