# Supplementary material for: New Insights into the Organization, Recombination, Expression and Functional Mechanism of Low Molecular Weight Glutenin Subunit Genes in Bread Wheat
Source: PLoS One. 2010 Oct 21;5(10):e13548. doi: 10.1371/journal.pone.0013548 (PMC2958824; doi:10.1371/journal.pone.0013548)
Supplement: Table S7 — Matching LMW-GS protein spots resolved by 2-DE to proteins predicted from the cloned active LMW-GS genes using mass spectragraphs generated by LC-MS/MS analysis in Jing 411. (0.05 MB PDF) [file pone.0013548.s012.pdf]

Table S7. Matching LMW-GS protein spots resolved by 2-DE to the proteins predicted from the cloned active LMW-GS genes using the mass spectra generated by LC-MS/MS analysis in Jing 411

Spot	Mass spectrum [a]	MH^{+} [b]	Charge	XC [c]	Matching gene	Predicted LMW-GS protein sequence [d]
5 and 6	L.GQQPQQQQL.A	1055.13	2	2.33	*B3-2*	QQTLSHHHHQQPIQQQPQQFPQQQPCSQQQQQPPLSQ
	L.GQQPQQQQL.A	1055.13	1	1.41		QQQPPFSQQQQPPF**SQQQQPVLPQQPSF**SQQQLPPFS
	L.GQC#VSQPQQQSQQQL.G	1744.84	2	2.85		QQQQPPFSQQQQPVLPQQPSFSQQQLPPF**SQQLPPF**SQ
	L.GQC#VSQPQQQSQQQLGQQPQQQQL.A	2780.94	3	3.18		QQQPVLPQQPPFSQQQLPPF**SQQLPPF**SQQQQPVLPQQ
	F.GVGTGVGGY.-	766.82	1	1.38		PPFSQQQQQPILPQQPPF**SQQQQPVLLQQQIPFVHPSI**
	F.VHPSIL.Q	665.80	1	1.04		**LQQLNPCKVF**LQQQCSPVAMPQSLARSQMLQQSSCH
	F.LQPHQIAQL.E	1048.22	2	2.31		VMQQQCCQQLPQIPQQSRY**EAIRAIVYSIIL**QEQQQVQ
	Y.EAIRAIVY.S	935.10	2	1.89		GSIQTQQQQPQQL**GQCVSQPQQQSQQQLGQQPQQQ**
	Y.EAIRAIVY.S	935.10	1	1.49		**QL**AQGTF**LQPHQIAQL**EVMTSIALRTLPTMCNVNVSL
	F.SQQQQPVLL.Q	1041.18	2	1.59		YRTTTRVPF**GVGTGVGGY**
	F.SQQQQPVLL.Q	1041.18	1	1.81		
	L.QQLNPC#KVF.L	1134.30	2	2.04		
	W.SIIL.P	445.58	1	1.12		
	F.SQQQQPVLPQQPSF.S	1612.77	2	2.28		
	F.SQQQQPVLPQQPPF.S	1622.81	2	2.80		
	F.SQQQQQPILPQQPPF.S	1764.96	2	2.59		
	L.GQQPQQQQL.A	1055.13	2	2.33		
	L.GQQPQQQQL.A	1055.13	1	1.41		
	L.GQC#VSQPQQQSQQQL.G	1744.84	2	2.85		
	F.GVGTGVGGY.-	766.82	1	1.36		
	L.GQC#VSQPQQQSQQQLGQQPQQQQL.A	2780.94	3	3.18		
	F.GVGTGVGGY.-	766.82	1	1.89		

	F.VHPSIL.Q	665.80	1	1.26		
	Y.EAIRAIVY.S	935.10	1	1.64		
	L.QQQIPF.V	760.86	1	1.19		
	F.SQQQQPVLL.Q	1041.18	1	1.83		
	F.SQQQQPVLPQQPSF.S	1612.77	2	2.82		
	F.SQQLPPF.S	816.92	1	1.34		
	F.SQQQQPVLPQQPPF.S	1622.81	2	2.57		
	F.SQQQQQPILPQQPPF.S	1764.96	2	3.23		
10 and 11	F.PQQQQPL.L	838.93	1	1.22	*D3-1*	MKTFLIFALLAVAATSAIAQMETSHIPGLEKPSQQQPLP
	F.QRPQQQQL.G	1026.13	2	1.86		LQQILWY**HQQQPIQQQPQPF**PQQPPCSQQQQPPLSQQ
	L.NQPQQQQPQQSVQGVSQPQQQQKQL.G	2889.09	3	5.97		QQPPFSQQQPPFSQQELPILPQQPPFSQQQQPQF**SQQQ**
	L.NQPQQQQPQQSVQGVSQPQQQQKQL.G	2889.09	2	3.36		**QPFPQQQQPL**L**LQQPPF**SQQRPPFSQQQQPVLPQQP
	F.GVGTQVGAY.-	851.93	1	1.53		PFSQQQQQQPILPQQPPFSQHQQPVLPQQQIPY**VQPSIL**
	Y.HQQQPIQQQPQPF.P	1604.75	2	3.34		**QQLNPCKVF**LQQQCSPVAMPQSLARSQMLWQSSCHV
	W.YHQQQPIQQQPQPF.P	1767.93	2	2.81		MQQQCCQQLPRIPEQSRY**DAIRAIIYSIVL**QEQQHGQG
	F.SQQQQPFPQQQQPL.L	1682.82	2	2.71		F**NQPQQQQPQQSVQGVSQPQQQQKQL**GQCSF**QRP**
	F.LQPHQIAQL.E	1048.22	2	2.85		**QQQQLGQWPQQQQVPQGTLLQPHQIAQL**ELMTSIA
	L.LQQPPF.S	729.85	1	1.30		L**RTLPMMCSVNVPVY**GTTTSVPF**GVGTQVGAY**
	L.GQWPQQQQVPQGTL.L	1595.74	2	2.93		
	L.RTLPM*M*C#SVNVPVY.G	1699.89	2	1.66		
	L.QQLNPC#KVF.L	1134.30	2	2.41		
	L.QQLNPC#KVF.L	1134.30	1	1.24		
	Y.SIVLQEQQHGQGF.N	1471.60	2	3.87		
	Y.DAIRAIIY.S	935.10	2	1.86		
	Y.DAIRAIIY.S	935.10	1	1.58		
	L.RTLPM*MC#SVNVPVY.G	1683.95	2	1.52		

	Y.VQPSIL.Q	656.79	1	1.46		
12 and 13	F.QQPQQQQL.G	998.08	2	2.03	*B3-1*	MKTFLIFALLAVAATSAIAQMETSHIPSLEKPL**QQQPLP**
	F.GVGTRVGAY.-	879.98	2	2.74		**LQQILW**YQQQQPIQQQPQPFPQQPPCSQQQQPPLSQQ
	F.GVGTRVGAY.-	879.98	1	1.61		QQPPFSQQQPPFLQQQQPV**LPQQPPF**SQQQQQFPQQQ
	F.QQPQQQQL.G	998.08	1	2.09		QPLLPQQPPFSQQQPPFSQQQQQPPFSQQQQQPILPQQP
	L.NQPQQQQPQQSVQGVSQPQQQQKQL.G	2889.09	3	6.14		PF**SQHQQPVLPQQQIPSVQPSIL**QQLNPCKVFLQQQC
	L.NQPQQQQPQQSVQGVSQPQQQQKQL.G	2889.09	2	3.80		SPVAMPQSLARSQMLWQSSCHVMQQQCCRQLPQIPEQ
	Y.SIVLQEQQHGQGL.N	1437.58	2	3.20		SRY**DAIRAIIYSIVLQEQQHGQGLNQPQQQQPQQSV**
	L.LPQQPPF.S	826.96	1	1.04		**QGVSQPQQQQKQL**GQCSF**QQPQQQQLGQWPQQQ**
	L.GQWPQQQQVPQGTL.L	1595.74	2	3.17		**QVPQGTL**LQPHQIAQLEVMTSIAL**RTLPTMCSVNVPV**
	L.RTLPTM*C#SVNVPVY.G	1653.86	2	1.66		**YGTTTIVPFGVGTRVGAY**
	L.QQQPLPL.Q	823.96	1	1.48		
	Y.DAIRAIIY.S	935.10	1	1.58		
	L.RTLPTMC#SVNVPVY.G	1637.92	2	2.09		
	L.QQILW.Y	687.81	1	1.31		
	Y.GTTTIVPF.G	835.97	1	1.40		
	F.SQHQQPVLPQQQIPSVQPSIL.Q	2353.66	2	4.04		
14	L.AQGTF.L	523.56	1	1.31	*D3-3*	MKTFLIFALLAVAATSAIAQIENSHIPGLEKPSQQQPLPL
	L.GQQPQQQQL.A	1055.13	2	2.26		QQTLSHHQQQQPVQQQPQPFPQQQPCSQQQQPPLSQQ
	L.GQQPQQQQL.A	1055.13	1	1.59		QQPPF**SQQQPPF**SQQQQPSFSQQQQPPFSQQQPPFSQQ
	L.ARSQML.Q	705.85	1	1.52		QQPVIPQQPSFSQQQLPPFSQQQPPFSQQQQPVLPQQPP
	L.GQC#VSQPQQQSQQQL.G	1744.84	2	3.24		FSQQQQPILPQQPPF**SQQQQQPVLPQQQIPFVHPSILQ**
	F.GVGAGVGAY.-	750.82	1	1.94		**QLNPCKVF**LQQQCSPVAMPQSL**ARSQML**QQSSCHVM
	F.SQQQPPF.W	831.90	1	1.99		QQQCCQQLPQIPQQSRY**EAIRAIIYSIIL**QEQQQVQGSI
	Y.RTTTSVPF.D	909.02	1	1.39		QSQQQQPQQL**GQCVSQPQQQSQQQLGQQPQQQQL**
	F.LQPHQIAQL.E	1048.22	2	2.51		**AQGTFLQPHQIAQL**EVMTSIALRILPTMCRVNVPL**YR**

	L.YRTTTSVPF.D	1072.20	1	1.22		**TTTSVPFGVGAGVGAY**
	F.VHPSIL.Q	665.80	1	1.33		
	L.QQLNPC#KVF.L	1134.30	2	2.42		
	W.SIIL.P	445.58	1	1.12		
	Y.EAIRAIIY.S	949.13	1	1.43		
	Y.EAIRAIIY.S	949.13	2	1.51		
	F.SQQQQQPVLPQQQIPF.V	1895.11	2	2.28		
24	F.SQQQQL.F	731.78	1	1.41	*D3-2*	MKTFLVFALLAVAATSAIAQMETRCIPGLERPWQQQPL
	F.PQQQQHQQL.V	1135.22	2	2.52		**PPQQTFPQQPL**F**SQQQQL**FPQQPSF**SQQQPPFW**QQQ
	L.AQGTF.L	523.56	1	1.18		PPFSQQQPILPQQPPFSQQQQL**VLPQQPPF**SQQQQPVL
	L.GQQPQQQQL.A	1055.13	2	2.35		PPQQSPF**PQQQQHQQLVQQQIPVVQPSILQQLNPCK**
	L.GQQPQQQQL.A	1055.13	1	1.43		**L**FLQQQCSPVAMPQRL**ARSQML**QQSSCHVMQQQCCQ
	L.ARSQML.Q	705.85	1	1.51		QLPQIPQQSRY**EAIRAIIY**SIILQEQQQVQGSIQSQQQQP
	L.GQC#VSQPQQQSQQQL.G	1744.84	2	3.56		QQL**GQCVSQPQQQSQQQLGQQPQQQQLAQGTF**LQ
	L.QQLNPC#KL.F	1001.15	1	1.05		PHQIAQLEVMTSIAL**RILPTMCSVNVPLYRTTTSVPFD**
	F.DVGTGVGAY.-	838.89	1	1.71		**VGTGVGAY**
	L.QQLNPC#KL.F	1001.15	2	1.55		
	L.YRTTTSVPF.D	1072.20	1	1.34		
	L.VLPQQPPF.S	926.09	1	1.16		
	Y.EAIRAIIY.S	949.13	2	1.66		
	Y.EAIRAIIY.S	949.13	1	1.78		
	L.PPQQTFPQQPL.F	1281.44	1	1.91		
	L.RILPTM*C#SVNVPL.Y	1516.76	2	2.17		
	F.SQQQPPFW.Q	1018.11	1	1.55		
	L.VQQQIPVVQPSIL.Q	1449.72	2	2.51		
	L.VQQQIPVVQPSIL.Q	1449.72	1	2.70		

	L.RILPTMC#SVNVPL.Y	1500.82	2	2.22		
25	L.AQGTF.L	523.56	1	1.51	*B3-3*	IPF**VHPSILQQLNPCKVF**LQQQCSPVAMPQSLARSQM
	L.GQQPQQQQL.A	1055.13	2	2.65		LQQSSCHVMQQQCCQQLPQIPQQSRY**EAIRAIIYSIIL**Q
	L.GQQPQQQQL.A	1055.13	1	1.31		EQQQVQGSIQSQQQQPQQL**GQCVSQPQQQSQQQLG**
	L.GQC#VSQPQQQSQQQL.G	1744.84	2	2.86		**QQPQQQQLAQGTF**LQPHQIAQLEVMTSIALRTLPTM
	F.GVGTGVGGY.-	766.82	1	1.59		CRVNVPLYRTTTSVPFG
	L.GQC#VSQPQQQSQQQLGQQPQQQQL.A	2780.94	3	3.81		
	F.VHPSIL.Q	665.80	1	1.33		
	Y.EAIRAIVY.S	935.10	2	1.87		
	Y.EAIRAIVY.S	935.10	1	1.38		
	L.QQQIPF.V	760.86	1	1.01		
	F.SQQQQPVLL.Q	1041.18	1	1.09		
	L.QQLNPC#KVF.L	1134.30	2	2.26		
	W.SIIL.P	445.58	1	1.00		
	F.SQQQQPVLPQQPSF.S	1612.77	2	1.58		
	F.SQQQQPVLPQQPPF.S	1622.81	2	1.87		
	F.SQQQQQPILPQQPPF.S	1764.96	2	2.43		
26 and 27	Y.QQQQPQQL.G	998.08	2	2.03	*A3-4*	MKTFLVFALLALAAASAVAQISQQQQQPPFSQQQQPPF
	Y.QQQQPQQL.G	998.08	1	1.56		**SQQQQSPF**SQQQQQPPFLQQQQPPFSQQPPISQQQQPP
	L.GQC#VSQPQQQL.Q	1273.37	2	2.46		FSQQQQPQFSQQQQPPYSQQQQPPYSQQQQPPFSQQQ
	L.QQQLGQQPQQQQL.A	1552.68	2	3.57		QPPFSQQQQPPFSQQQQQPPFTQQQQPSF**SQQPPISQQ**
	F.SQQQQSPF.S	949.99	1	1.14		**QQQQQQQQQPF**TQQQQPPFSQQPPISQQQQPPFSQQQ
	F.SQQPPISQQQQQQQQQQPF.T	2410.55	2	3.89		QPPFSQQQQIPVIHPSVL**QQLNPCKVF**LQQQCIPVAMQ
	L.GQC#VSQPQQQLQQQL.G	1770.92	2	3.21		RCLARSQMLQQSICHVMQQQCCQQLRQIPEQSRHESIR
	L.QQLNPC#KVF.L	1134.30	2	2.05		AIIY**SIILQQQQQQQQQQQQGQSIIQYQQQQPQQLG**
	L.RTLPTM*C#SVNVPL.Y	1504.71	2	2.52		**QCVSQPQQQLQQQLGQQPQQQQL**AHGTFLQPHQIA

	L.GVGIGVGVY.-	820.96	1	1.39		QLEVMTSIAL**RTLPTMCSVNVPL**YETTTSVPL**GVGIG VGVY**
	Y.SIILQQQQQQQQQQQQGQSIIQY.Q	2773.01	2	4.36		
	F.SQQQQIPVIHPSVL.Q	1574.81	2	3.30		

[a] The “#” and “*” symbols in the peptides denote the cysteine residue with carbamidomethyl and the methionine residue with oxidation modifications, respectively.

[b] MH^+ , the m/z of protonated molecular ion of the corresponding peptide.

[c] Cross-correlation value computed from cross-correlating the experimental MS/MS spectrum vs candidate peptides in the database (significant score: ≥1 for single-charged ions, ≥1.5 for doubly-charged ions, ≥2.5 for triply-charged ions).

[d] The peptides written in blue in the predicted protein represent those identified by LC-MS/MS from the corresponding excised protein spot.
