# Supplementary material for: New Insights into the Organization, Recombination, Expression and Functional Mechanism of Low Molecular Weight Glutenin Subunit Genes in Bread Wheat
Source: PLoS One. 2010 Oct 21;5(10):e13548. doi: 10.1371/journal.pone.0013548 (PMC2958824; doi:10.1371/journal.pone.0013548)
Supplement: Table S8 — Matching LMW-GS protein spots resolved by 2-DE to proteins predicted from cloned active LMW-GS genes using the mass spectragraphs generated by LC-MS/MS analysis in Ae. tauschii accession As91. (0.05 MB PDF) [file pone.0013548.s013.pdf]

Table S8. Matching LMW-GS protein spots resolved by 2-DE to the proteins predicted from the cloned active LMW-GS genes using the mass spectra generated by LC-MS/MS analysis in the *Ae. tauschii* accession As91

Spot	Mass spectrum [a]	MH^{+} [b]	Charge	XC [c]	Matching gene	Predicted LMW-GS protein sequence [d]
1	F.SQQRPPF.S	859.95	2	1.60	*D3-1*	METSHIPGLEKPSQQQPLPL**QQILWYHQQQPIQQQPQPF**PQ
	F.GVGTQVGAY.-	851.93	1	1.30		QPPCSQQQQPPLSQQQQPPFSQQQPPFSQQELPILPQQPPFSQ
	W.YHQQQPIQQQPQPF.P	1767.93	2	3.93		QQQPQF**SQQQQPFPQQQQPL**LLQQPPF**SQQRPPF**SQQQQQ
	F.SQQQQPFPQQQQPL.L	1682.82	2	3.12		PVLPQQPPFSQQQQQQPLLLQQPPF**SQHQQPVLPQQQIPYV**
	Y.SIVLQEQQHGQGF.N	1471.60	2	3.57		**QPSILQQLNPCKVFL**QQQCSPVAMPQSLARSQMLWQSSCH
	Y.HQQQPIQQQPQPF.P	1604.75	2	2.19		VMQQQCCQQLPRIPEQSRY**DAIRAIIYSIVLQEQQHGQGF**N
	F.SQHQQPVLPQQQIPY.V	1791.99	2	2.65		QPQQQQPQQSVQGVSQPQQQQKQLGQCSFQRPQQQQLGQ
	Y.VQPSIL.Q	656.79	1	1.43		WPQQQQVPQGTL**LQPHQIAQL**ELMTSIALRTLPMMCSVNV
	L.QQLNPCKVF.L	1134.30	2	2.64		PVYGTTTSVPF**GVGTQVGAY**
	F.SQQQQQQPILPQQPPF.S	1893.09	2	2.06		
5	F.GVGTQVGAY.-	851.93	1	1.63		
	W.YHQQQPIQQQPQPF.P	1767.93	2	3.79		
	F.SQQQQPFPQQQQPL.L	1682.82	2	3.32		
	Y.HQQQPIQQQPQPF.P	1604.75	2	2.86		
	Y.SIVLQEQQHGQGF.N	1471.60	2	3.91		
	F.SQHQQPVLPQQQIPY.V	1791.99	2	3.50		
	L.QQLNPCKVF.L	1134.30	2	2.89		
	Y.DAIRAIIY.S	935.10	1	1.65		
	Y.VQPSIL.Q	656.79	1	1.68		
	L.QQILW.Y	687.81	1	1.34		
6	F.GVGTQVGAY.-	851.93	1	1.20		

	Y.HQQQPIQQQPQPF.P	1604.75	2	3.47		
	F.SQQQQPFPQQQQPL.L	1682.82	2	3.42		
	L.LQPHQIAQL.E	1048.22	2	2.53		
	L.LQPHQIAQL.E	1048.22	1	1.71		
	Y.SIVLQEQQHGQGF.N	1471.60	1	2.97		
	Y.SIVLQEQQHGQGF.N	1471.60	2	3.52		
	L.QQLNPCKVF.L	1134.30	1	1.14		
	Y.VQPSIL.Q	656.79	1	1.45		
	L.QQLNPCKVF.L	1134.30	2	2.48		
	Y.DAIRAIIY.S	935.10	2	1.91		
	Y.DAIRAIIY.S	935.10	1	1.66		
	L.QQILW.Y	687.81	1	1.47		
2	L.QQSPF.S	606.65	1	1.12	*D3-6*	MKTFLIFALLAIAATSAIAQMETSRVPGLEKPWQQQPLPPQQ
	F.SIGTGVGGY.-	810.88	1	1.90		QPPCSQQQQPF**PQQQQPIIILQQSPFSQQQQPVLPQQQPVI**
	F.LQPHQISQL.E	1064.22	2	2.13		**IL**QQPPF**SQQQQPVLPQQPPF**SQQQQQQQQQQPPFSQQQQ
	L.QQLNPCKVF.L	1134.30	2	2.32		PVLPQQPPFSQQQPPFSQQQQPSSQQPPFPQQHQQFPQQQIPV
	L.QQLNPCKVF.L	1134.30	1	1.13		VQPSVL**QQLNPCKVF**LQQQCSHVAMSQRLARSQMWQQSS
	Y.SIILQEQQQGF.V	1291.44	1	3.21		CHVMQQQCCQQLPQIPEQSRSEAIRAIVY**SIILQEQQQGF**V
	Y.SIILQEQQQGF.V	1291.44	2	4.69		QPQQQQPQQSGQGVSQHQQQSQQQQQLGQCSFQQPQQ**LQ**
	F.SQQQQPVLPQQPPF.S	1622.81	2	2.48		**QLGQQPQQQQIPQGIFLQPHQISQL**EVMTSIAL**RTLPTMC**
	F.PQQQQPIIIL.Q	1178.41	1	2.74		**GVNVPL**YSSTTSMPF**SIGTGVGGY**
	F.SQQQQPVLPQQQPVIIL.Q	1945.25	2	4.14		
	L.QQLGQQPQQQQIPQGIF.L	1967.18	2	1.94		
3	F.SIGTGVGGY.-	810.88	1	1.52		
	Y.SIILQEQQQGF.V	1291.44	2	3.97		
	F.SQQQQPVLPQQPPF.S	1622.81	2	2.76		

	Y.SIILQEQQQGF.V	1291.44	2	3.61		
	Y.SIILQEQQQGF.V	1291.44	1	2.11		
	L.QQLGQQPQQQQIPQGIF.L	1967.18	2	2.66		
	F.SQQQQPVLPQQQPVIIL.Q	1945.25	2	4.17		
12	F.SIGTGVGGY.-	810.88	1	1.86		
	F.LQPHQISQL.E	1064.22	2	1.67		
	F.LQPHQISQL.E	1064.22	1	1.52		
	F.PQQQQPIIIL.Q	1178.41	1	2.84		
	Y.SIILQEQQQGF.V	1291.44	2	4.37		
	Y.SIILQEQQQGF.V	1291.44	1	3.20		
	L.RTLPTM*CGVNVPL.Y	1474.75	2	2.47		
	F.SQQQQPVLPQQPPF.S	1622.81	2	2.38		
	F.SQQQQPVLPQQQPVIIL.Q	1945.25	2	4.13		
	L.QQLGQQPQQQQIPQGIF.L	1967.18	2	3.18		
7	F.GVGAGVGAY.-	750.82	1	1.72	*D3-3*	MKTFLIFALLAVAATSAIAQMENSHIPGLEKPSQQQPLPLQQT
	F.SQQQPPF.S	831.90	1	1.33		LSHHQQQQPVQQQPQPLPQQQPCSQQQQPPLSQQQQPPFSQ
	F.VHPSIL.Q	665.80	1	1.53		QQPPFSQQQQPSFSQQQQPPF**SQQQPPF**SQQQQPVLPQQPSF
	Y.RTTTSVPF.D	909.02	1	1.44		SQQQLPPFSQQQPPFSQQQQPVLPQQPPF**SQQQQPILPQQPP**
	L.LQPHQIAQL.E	1048.22	1	1.58		**FSQQQQQPVLPQQQIPFVHPSILQQLNPCKVF**LQQQCSPV
	L.YRTTTSVPF.D	1072.20	1	1.32		AMPQSLARSQMLQQSSCHVMQQQCCQQLPQIPQQSRY**EAI**
	L.QQLNPCKVF.L	1134.30	2	2.45		**RAIIY**SIILQEQQQVQGSIQTQQQQPQQLGQCVSQPQQQSQQ
	L.QQLNPCKVF.L	1134.30	1	1.85		QLGQQPQQQQLAQGTF**LQPHQIAQL**EVMTSIALRTLPTMCR
	F.SQQQQPVIPQQPSF.S	1612.77	1	3.10		VNVPLY**RTTTSVPFGVGAGVGAY**
	Y.EAIRAIIY.S	949.13	2	2.24		
	Y.EAIRAIIY.S	949.13	1	2.30		
	F.SQQQQPILPQQPPF.S	1636.83	2	3.18		

	F.SQQQQQPVLPQQQIPF.V	1895.11	1	3.38		
	L.SQQQQPPFSQQQPPF.S	1772.90	2	2.03		
	F.SQQQQQPVLPQQQIPF.V	1895.11	2	4.34		
8	W.QQQPPF.S	744.82	1	1.83	*D3-2*	MKTFLVFALLAVAATSAIAQMETRCIPGLERPWQQQPLPPQQ
	F.GVGTGVGAY.-	780.85	1	1.49		TFPQQPLFSQQQQLYPQQPSF**SQQQPPFWQQQPPFSQQQPI**
	Y.RTTTSVPF.D	909.02	1	1.35		**LPQQPPF**SQQQQL**VLPQQPPF**SQQQQPVLPPQHHLFHTTTT
	L.VLPQQPPF.S	926.09	1	1.65		HQQL**VQQQIPVVQPSILQQLNPCKVF**LQQQCSPVAMPQRL
	Y.EAIRAIIY.S	949.13	1	2.04		ARSQMLQQSSCHVMQQQCCQQLPQIPQQSRY**EAIRAIIY**SII
	Y.EAIRAIIY.S	949.13	2	2.14		LQEQQQVQGSIQSQQQQPQQLGQCVSQPQQQSQQQLGQQP
	F.SQQQPPFW.Q	1018.11	1	1.59		QQQQLAQGTF**LQPHQIAQL**EVMTSIALRILPTMCSVNVPLY
	L.YRTTTSVPF.D	1072.20	2	1.68		**RTTTSVPFGVGTGVGAY**
	L.QQLNPCKVF.L	1134.30	1	1.07		
	L.QQLNPCKVF.L	1134.30	2	2.63		
	L.VQQQIPVVQPSIL.Q	1449.72	1	3.51		
	L.VQQQIPVVQPSIL.Q	1449.72	2	3.73		
	F.SQQQPILPQQPPF.S	1508.70	1	2.97		
9	F.GVGTGVGAY.-	780.85	1	1.02		
	Y.RTTTSVPF.D	909.02	1	1.25		
	L.VLPQQPPF.S	926.09	1	1.06		
	Y.EAIRAIIY.S	949.13	1	1.65		
	L.LQPHQIAQL.E	1048.22	1	1.69		
	L.QQLNPCKVF.L	1134.30	2	2.76		
	L.VQQQIPVVQPSIL.Q	1449.72	2	3.05		
	L.VQQQIPVVQPSIL.Q	1449.72	1	3.43		
	F.SQQQPILPQQPPF.S	1508.70	1	2.95		
10	W.QQQPPF.S	744.82	1	1.36		

	Y.EAIRAIIY.S	949.13	1	1.68		
	L.LQPHQIAQL.E	1048.22	2	1.69		
	L.VQQQIPVVQPSIL.Q	1449.72	2	3.03		
	L.VQQQIPVVQPSIL.Q	1449.72	1	3.02		
13	F.GVGTGVGAY.-	780.85	1	1.62	*D3-7*	MKTFLVFALIAVVVTSAIAQMETSSISGLERPW**QQQPLPPQQ**<br>**SF**SQQPPFSQQQQQQPLPQQPSF**SQQQPPF**SQQQPIL**SQQPP**<br>**F**SQHQQPVLPQQSPFSQQQQLVLPPQQQQQL**VQQQIPIVQP**<br>**SVLQQLNPCKVF**LQQQCSPVAMPQRLARSQMWQQSSCHV<br>MQQRCCQQLQQIPEQSRY**EAIRAIIYSIILQEQQQGF**VQPQ<br>QQQPQQSGQGVSQSQQQSQQQLGQCSFQQPQQQLGQQPQQ<br>QQQQQQQVLQGTFLQPHQIAHLEVVLSIALRTLPTMCSVNV<br>PLYSATTSVPF**GVGTGVGAY**
	F.SQQQPPF.S	831.90	1	1.77		
	W.QQQPLPPQQSF.S	1298.43	1	2.04		
	F.SQQPPF.S	703.77	1	1.21		
	L.QQLNPCKVF.L	1134.30	1	1.54		
	L.QQLNPCKVF.L	1134.30	2	2.62		
	Y.EAIRAIIY.S	949.13	1	1.76		
	Y.SIILQEQQQGF.V	1291.44	2	4.81		
	Y.SIILQEQQQGF.V	1291.44	1	3.45		
	L.VQQQIPIVQPSVL.Q	1449.72	2	3.07		
	L.VQQQIPIVQPSVL.Q	1449.72	1	3.41		

[a] The "*" symbol in the peptide denotes the methionine residue with oxidation modification.

[b] MH^+ , the m/z of protonated molecular ion of the corresponding peptide.

[c] Cross-correlation value computed from cross-correlating the experimental MS/MS spectrum vs candidate peptides in the database (significant score: ≥1 for single-charged ions, ≥1.5 for doubly-charged ions).

[d] The peptides written in blue in the predicted protein represent those identified by LC-MS/MS from the corresponding excised protein spot.
