# Supplementary material for: New Insights into the Organization, Recombination, Expression and Functional Mechanism of Low Molecular Weight Glutenin Subunit Genes in Bread Wheat
Source: PLoS One. 2010 Oct 21;5(10):e13548. doi: 10.1371/journal.pone.0013548 (PMC2958824; doi:10.1371/journal.pone.0013548)
Supplement: Table S9 — Matching LMW-GS protein spots resolved by 2-DE to proteins predicted from cloned and active LMW-GS genes using mass spectra generated by LC-MS/MS analysis in Ae. tauschii accession Y207. (0.03 MB PDF) [file pone.0013548.s014.pdf]

Table S9. Matching LMW-GS protein spots resolved by 2-DE to the proteins predicted from the cloned and active LMW-GS genes using the mass spectra generated by LC-MS/MS analysis in the *Ae. tauschii* accession Y207

Spot	Mass spectrum [a]	MH^{+} [b]	Charge	XC [c]	Matching gene	Predicted LMW-GS protein sequence [d]
1	Y.SIVLQEQQHGQGF.N	1471.60	2	2.23	*D3-1*	METSHIPGLEKPSQQQPLPLQQILW**YHQQQPIQQQPQPF**PQQPPCSQ
	Y.VQPSIL.Q	656.79	1	1.54		QQQPPLSQQQQPPFSQQQPPFSQQELPILPQQPPFSQQQQPQFSQQQQ
	F.GVGTQVGAY.-	851.93	1	1.18		PFPQQQQPLLLQQPPF**SQQRPPF**SQQQQQPVLPQQPPFSQQQQQQPI
	L.GQCSF.Q	598.62	1	1.04		LPQQPPFSRHQQPVLPQQQIPY**VQPSIL**QQLNPCKVFLQQQCSPVAM
2	W.YHQQQPIQQQPQPF.P	1767.93	2	3.30		PQSLARSQMLWQSSCHVMQQQCCQQLPRIPEQSRYDAIRAIIY**SIVL**
	Y.HQQQPIQQQPQPF.P	1604.75	2	3.21		**QEQQHGQGF**NQPQQQQPQQSVQGVSQPQQQQKQL**GQCSFQRPQ**
	Y.SIVLQEQQHGQGF.N	1471.60	2	2.44		**QQQL**GQWPQQQQVPQGTLLQPHQIAQLELMTSIALRTLPMMCSVN
	F.SQQRPPF.S	859.95	2	1.55		VPVYGTTTSVPF**GVGTQVGAY**
	F.QRPQQQQL.G	1026.13	2	1.50		
	F.GVGTQVGAY.-	851.93	1	1.42		
	L.GQCSF.Q	598.62	1	1.12		
	Y.VQPSIL.Q	656.79	1	1.11		
3	Y.SIILQEQQQGF.V	1291.44	2	3.67	*D3-6*	MKTFLIFALLAIAATSAIAQMETSRVPGLEKPWQQQPLPPQQQPPCSQ
	F.SQQQQPVLPQQPPF.S	1622.81	2	2.88		QQQPF**PQQQQPIIILQQSPFSQQQQPVLPQQQPVIIL**QQPPF**SQQQ**
	F.PQQQQPIIIL.Q	1178.41	1	2.01		**QPVLPQQPPF**SQQQQPPFSQQQQPVLPQQPPFSQQQQPPFSQQQQPS
	F.QQPQQL.Q	741.82	1	1.03		SQQPPFPQQHQQFPQQQIPVVQPSVLQQLNPCKVFLQQQCSHVAMS
	L.EVM*TSIAL.R	880.04	1	1.00		QRLARSQMWQQSSCHVMQQQCCQQLPQIPEQSRYEAIRAIVY**SIILQ**
4	Y.SIILQEQQQGF.V	1291.44	2	3.78		**EQQQGF**VQPQQQQPQQSGQGVSQHQQQSQQQQQLGQCSF**QQPQQ**
	F.SQQQQPVLPQQQPVIIL.Q	1945.25	2	3.26		**L**QQLGQQPQQQQIPQGIF**LQPHQISQLEVMTSIALRTLPTMCGVN**
	F.SQQQQPVLPQQPPF.S	1622.81	2	2.86		**VPL**YSSTTSMPFSIGTGVGGY
	F.PQQQQPIIIL.Q	1178.41	1	2.34		

	F.LQPHQISQL.E	1064.22	2	1.92		
	L.RTLPTM*CGVNVPL.Y	1474.75	2	1.62		
	L.QQSPF.S	606.65	1	1.15		
5	F.SQQQQPILPQQPPF.S	1636.83	2	3.82	*D3-3*	MKTFLIFALLAVAATSAIAQMENSHIPGLEKPSQQQPLPLQQTLSHQQ
	F.SQQQQQPVLPQQQIPF.V	1895.11	2	3.66		QQQPVQQQPQPFPQQQPCSQQQQPPL**SQQQQPPFSQQQPPFSQQQ**
	F.SQQQQPVLPQQPPF.S	1622.81	2	3.57		**QPSFSQQQQPPFSQQQPPF**SQQQQPVLPQQPSFSQQQLPPFSQQQPP
	F.SQQQQPVIPQQPSF.S	1612.77	2	3.31		F**SQQQQPVLPQQPPFSQQQQPILPQQPPFSQQQQQPVLPQQQIPF**
	L.GQQPQQQQL.T	1055.13	2	3.26		**VHPSILQQLNPCKVF**LQQQCSPVAMPQSLARSQMLQQSSCHVMQQ
	L.SQQQQPPFSQQQPPF.S	1772.90	2	3.09		QCCQQLPQIPQQSRY**EAIRAIIY**SIILQEQQQVQGSIQTQQQQPQQL**G**
	L.GQCVSQPQQQSQQQL.G	1744.84	2	3.04		**QCVSQPQQQSQQQL**GQQPQQQQL**AQGTF**LQPHQIAQLEVMTSIAL
	F.SQQQPPFSQQQQPSF.S	1762.86	2	2.84		RTLPTMCRVNVPLYRTTTSVPF**GVGAGVGAY**
	L.QQLNPCKVF.L	1134.30	2	2.59		
	F.SQQQPPF.S	831.90	1	2.06		
	F.GVGAGVGAY.-	750.82	1	1.95		
	L.SQQQQPPF.S	960.03	1	1.80		
	Y.EAIRAIIY.S	949.13	2	1.75		
	F.VHPSIL.Q	665.80	1	1.47		
	L.AQGTF.L	523.56	1	1.37		
6	F.GVGTGVGAY.-	780.85	1	1.35	*D3-2*	VFALLAVAATSAIAQMETRCIPGLERPWRQQPLPPQQTFPQQPLFSQQ
	L.EVMTSIAL.R	864.04	1	1.09		QQLFPQQPSFSQQQPPFWQQQPPFSQQQPILPQQPPFSQQQQL**VLPQ**
	L.VLPQQPPF.S	926.09	1	1.25		**QPPF**SQQQQPVLPPQQSPF**PQQQQQHQQLVQQQIPVVQPSILQQL**
	Y.EAIRAIIY.S	949.13	1	1.47		**NPCKVF**LQQQCSPVAMPQRLARSQMLQQSSCHVMQQQCCQQLPQI
	L.GQQPQQQQL.T	1055.13	2	2.84		PQQSRY**EAIRAIIY**SIILQEQQQVQGSIQSQQQQPPQL**GQCVSQPQQ**
	L.GQQPQQQQL.T	1055.13	1	1.25		**QSQQQLGQQPQQQQL**AQGTFLQPHQIAQL**EVMTSIAL**RILPTMCS
	L.QQLNPCKVF.L	1134.30	2	2.34		VNVPLYRTTTSVPF**GVGTGVGAY**
	F.PQQQQQHQQL.V	1263.35	2	2.42		

	L.VQQQIPVVQPSIL.Q	1449.72	1	2.28		
	L.VQQQIPVVQPSIL.Q	1449.72	2	3.14		
	L.GQCVSQPQQQSQQQL.G	1744.84	2	3.78		

[a] The “*” symbol in the peptides denotes the methionine residue with oxidation modification.

[b] MH^+ , the m/z of protonated molecular ion of the corresponding peptide.

[c] Cross-correlation value computed from cross-correlating the experimental MS/MS spectrum vs candidate peptides in the database (significant score: ≥1 for single-charged ions, ≥1.5 for doubly-charged ions).

[d] The peptides written in blue in the predicted protein represent those identified by LC-MS/MS from the corresponding excised protein spot.
